# Penalized G-estimation for effect modifier selection in a structural nested mean model for repeated outcomes

**DOI:** 10.1093/biomtc/ujae165

**Published:** 2025-01-07

**Authors:** Ajmery Jaman, Guanbo Wang, Ashkan Ertefaie, Michèle Bally, Renée Lévesque, Robert W. Platt, Mireille E. Schnitzer

**Affiliations:** 1 Department of Epidemiology, Biostatistics and Occupational Health, McGill University, Montreal, QC H3A 1G1, Canada; 2 CAUSALab, Department of Epidemiology, Harvard T.H. Chan School of Public Health, Boston, MA 02115, United States; 3 Department of Biostatistics, Epidemiology and Informatics, University of Pennsylvania, Philadelphia, PA 19104, United States; 4 Department of Pharmacy, Centre Hospital of University of Montreal, Montreal, QC H2X 0C1, Canada; 5 Faculty of Pharmacy, University of Montreal, Montreal, QC H3C 3J7, Canada; 6 Department of Medicine, University of Montreal, Montreal, QC H3T 1J4, Canada

**Keywords:** double robustness, effect modifier selection, G-estimation, hemodiafiltration, longitudinal observational data, penalization

## Abstract

Effect modification occurs when the impact of the treatment on an outcome varies based on the levels of other covariates known as effect modifiers. Modeling these effect differences is important for etiological goals and for purposes of optimizing treatment. Structural nested mean models (SNMMs) are useful causal models for estimating the potentially heterogeneous effect of a time-varying exposure on the mean of an outcome in the presence of time-varying confounding. A data-adaptive selection approach is necessary if the effect modifiers are unknown *a pri ori* and need to be identified. Although variable selection techniques are available for estimating the conditional average treatment effects using marginal structural models or for developing optimal dynamic treatment regimens, all of these methods consider a single end-of-follow-up outcome. In the context of an SNMM for repeated outcomes, we propose a doubly robust penalized G-estimator for the causal effect of a time-varying exposure with a simultaneous selection of effect modifiers and prove the oracle property of our estimator. We conduct a simulation study for the evaluation of its performance in finite samples and verification of its double-robustness property. Our work is motivated by the study of hemodiafiltration for treating patients with end-stage renal disease at the Centre Hospitalier de l’Université de Montréal. We apply the proposed method to investigate the effect heterogeneity of dialysis facility on the repeated session-specific hemodiafiltration outcomes.

## 1 INTRODUCTION

When a statistical goal is to estimate the causal effect of a treatment or an exposure on an outcome, effect modification or heterogeneity in the treatment effect may be of interest. Effect modification occurs when the effect of a treatment on an outcome differs according to the values of some other variables, usually pre-treatment covariates; such variables are called effect modifiers (EMs; VanderWeele and Robins, 2007). Modeling these effect differences is important for etiological goals and for purposes of optimizing treatment. Analyzing effect modification has gained attention in recent years because of the popularization of precision medicine (Ashley, 2015) and dynamic treatment regimes (Murphy, 2003; Chakraborty and Moodie, 2013; Chakraborty and Murphy, 2014; Wallace and Moodie, 2015), which deal with personalizing treatments by incorporating patient-level information to optimize expected outcomes. Structural nested mean models (SNMMs) are useful causal models for estimating the potentially heterogeneous effect of a time-varying exposure on the mean of an outcome in the presence of time-varying confounding (Robins, 1989, 1997; Robins et al., 2007).

Our methodological development is motivated by an application in nephrology regarding patients with end-stage renal disease, which is the final, permanent stage of chronic kidney disease. Patients with end-stage renal failure must undergo a kidney transplant or regular dialysis to survive for more than a few weeks because the kidneys no longer function properly. Hemodiafiltration (HDF) is a dialysis technique that is used for cleaning the blood from waste and excess fluid. HDF combines 2 processes (Ronco and Cruz, 2007): diffusion (where solute molecules passively move from the blood to the dialysis fluid) and convection (where larger molecules are cleared from the blood). Hemodiafiltration is routinely used for patients with end-stage renal disease treated at the University of Montreal Hospital Centre (CHUM) outpatient dialysis clinic and its affiliated ambulatory dialysis center (CED). With hemodiafiltration, a large volume of plasma water is ultrafiltered, and this requires the administration of a substitution fluid back to the patient in order to maintain fluid balance. Convection volume is calculated as the sum of the substitution volume and the ultrafiltration volume (Marcelli et al., 2015). The effectiveness of hemodiafiltration is indicated by the convection volume attained during each session. The CONvective TRAnsport STudy (Grooteman et al., 2012) randomized controlled trial and a meta-analysis of individual patient-level data from randomized controlled trials showed that hemodiafiltration, compared to hemodialysis, reduced the risk of all-cause mortality by ~22% and of cardiovascular disease mortality by 31%, over a median follow-up of 2.5 years in the hemodiafiltration treatment subgroup achieving high convection volumes (Peters et al., 2016). These results suggest that one should aim for a convection volume of at least 24 L per session (Chapdelaine et al., 2015). On average, convection volumes recorded at the outpatient dialysis clinic located at the CHUM are lower than those at CED. This has triggered an interest in investigating if there is any effect of the dialysis facility (CHUM versus CED) on the convection volume or for which patients such an effect exists. Available data from hospital records include sociodemographic information, diagnoses, medications, blood test results, and dialysis treatment parameters of all dialysis sessions per patient in the study time frame (March 1, 2017 to December 1, 2021). Convection volume outcomes were measured at the end of each successful dialysis session, enabling us to investigate the average effect of the dialysis facility on the session-specific mean convection volume. Since it is currently unknown which measured variables could modify the effect of the dialysis facility on the hemodiafiltration outcome, it is important to develop a data-adaptive approach to selecting EMs in this context.

Variable selection techniques are available in estimation of the average causal effect (Shortreed and Ertefaie, 2017; Koch et al., 2018; Tang et al., 2023), the conditional average treatment effects using marginal structural models (Bahamyirou et al., 2022), and in developing optimal dynamic treatment regimens (Gunter et al., 2011; Shi et al., 2018; Wallace et al., 2019; Bian et al., 2021, 2023; Jones et al., 2022; Moodie et al., 2023). All of these methods were largely developed for dynamic or non-dynamic treatment interventions, some in longitudinal settings, where the outcome is measured at a single point in time. Under a traditional semiparametric regression approach (targeting prediction), Johnson et al. (2008) proposed penalized estimating equations for variable selection with a univariate outcome, whereas Wang et al. (2012) extended this work to high-dimensional longitudinal data proposing penalized generalized estimating equations. Boruvka et al. (2018) discussed assessments of causal effect moderation (modification) in mobile health interventions, where treatment, response, and potential moderators are all time-varying. A recent study proposed sequential knockoffs for variable selection in the Markov decision process framework to address reinforcement learning problems (Ma et al., 2023). However, to the best of our knowledge, no method exists that conducts simultaneous EM selection and causal effect estimation with longitudinal data and repeated outcomes. Motivated by our application, we seek to develop a doubly robust estimator for the causal effect of an exposure with simultaneous selection of EMs in the SNMM for repeated outcomes. Our estimator facilitates sparse modeling, which is important for developing a tractable model when covariate dimensionality is high and improves precision in estimation by eliminating spurious EMs.

This paper is organized as follows. In Section 2, we introduce the notation, describe the model and assumptions, present methodological details, and provide theoretical results for the asymptotic properties of the proposed estimator. We present a simulation study to evaluate the performance of our estimator in finite samples in Section 3 and describe the application of the proposed method to the hemodiafiltration data in Section 4. Finally, we provide a discussion in Section 5.

## 2 METHODOLOGY

### 2.1 Notation and model with assumptions

Suppose we have data from n patients, and all patients have measurements from J sequential hemodiafiltration sessions. At each session, the information of the outcome, the treatment received, and pre-session covariates are recorded. For patient i at session j, denote the observed continuous outcome by $Y_{ij}$, the (binary) treatment received by $A_{ij}$, and the vector of covariates by $L_{ij},\forall i=1,\ldots ,n,j=1,\ldots ,J$. Let $H_{ij}$ represent the histories of covariates ${\bar{L}}_{ij}=\left\{L_{i1},\ldots L_{ij}\right\}$, past exposures ${\bar{A}}_{i(j-1)}=\left\{A_{i1},\ldots ,A_{i(j-1)}\right\}$, and past outcomes ${\bar{Y}}_{i(j-1)}=\left\{Y_{i1},\ldots ,Y_{i(j-1)}\right\}$. Throughout this paper, we use the potential outcomes framework (Robins, 1989). Define $Y_{ij}\left({\bar{a}}_{j}\right)$ as the counterfactual outcome that would have been observed at occasion j for patient i if the treatment history ${\bar{A}}_{ij}=\left\{A_{i1},\ldots ,A_{ij}\right\}$ were set counterfactually to ${\bar{a}}_{j}=\left\{a_{1},\ldots ,a_{j}\right\}$. To model the proximal (short-term) effects of the exposure, a linear SNMM can be defined as (Robins, 1989; Vansteelandt and Joffe, 2014)

(1) $$E\left\{Y_{ij}\left({\bar{a}}_{j-1},a_{j}\right)-Y_{ij}\left({\bar{a}}_{j-1},0\right)|H_{ij}=h_{ij},A_{ij}=a_{j}\right\}\;\;=\gamma_{j}^{*}\left(a_{j},h_{ij};\psi \right)$$

for each measurement occasion j=1,…,J, where $\gamma_{j}^{*}\left(a_{j},h_{ij};\psi \right)$, known as “treatment blip,” is a scalar-valued function smooth in $\psi ;h_{ij}$ represents the realized values for $H_{ij}$; and $\psi ={\left(\psi_{0},\psi_{1},\ldots ,\psi_{K-1}\right)}^{⊤}$ is a K-dimensional vector of parameters. The difference in (1) represents the effect of treatment $a_{j}$ versus the reference treatment 0 on the outcome at occasion j, given the history up to occasion j. Our goal is thus to estimate the parameters ψ using the observed data. The following assumptions are required to identify ψ from the observed data (Robins, 1989; Vansteelandt and Joffe, 2014; He et al., 2015).

- Consistency: The observed outcome is equal to the potential outcome at occasion j, for j=1,…,J, if the observed treatment history matches the counterfactual history at occasion j, that is, $Y_{ij}\left({\bar{a}}_{j}\right)=Y_{ij}$, if ${\bar{A}}_{ij}={\bar{a}}_{j}$.
- Sequential ignorability/conditional exchangeability: The potential outcome $Y_{ij}\left({\bar{a}}_{j-1},0\right)$ is independent of $A_{ij}$ conditional on $H_{ij}$, for j=1,…,J.
- Positivity: If the joint density of $H_{ij}$ at $\left\{h_{ij}\right\}$ is greater than 0, then $Pr\left(A_{ij}=a_{j}|H_{ij}=h_{ij}\right)>0$ for all $a_{j},j=1,\ldots ,J$.

**FIGURE 1:**
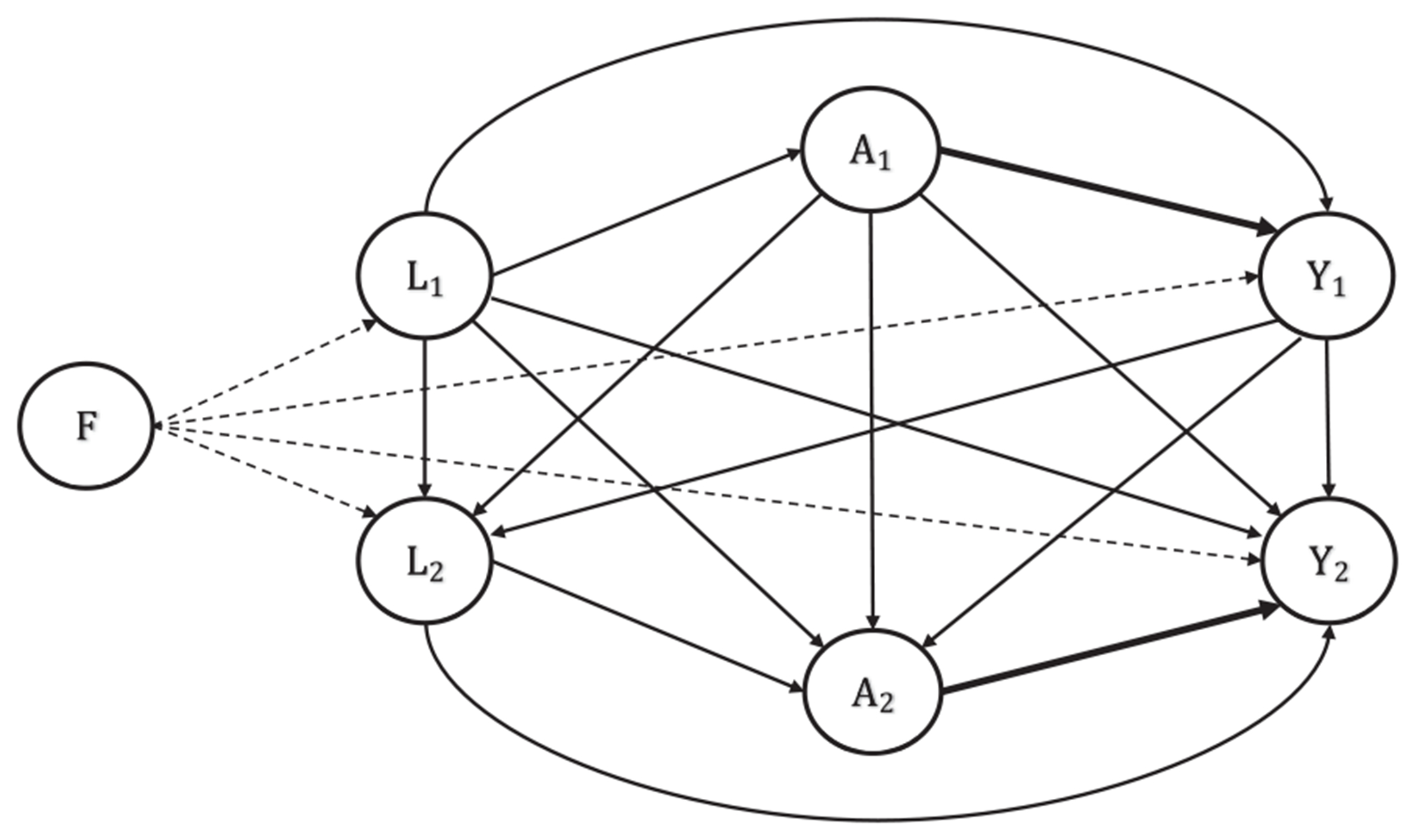
An example causal directed acyclic graph (DAG) representing the relationship among confounders, exposures, and outcomes in our time-varying setup.

To visualize this time-varying setup, the data-generating process for the first 2 measurement occasions is shown in the directed acyclic graph in Figure 1. In the figure, *F* represents the unmeasured variables that are not confounders. The bold arrows in the figure show the proximal effect of the treatment that we want to estimate.

### 2.2 G-estimation with repeated outcomes

G-estimation is used for estimating the parameters in SNMMs (Robins and Hernan, 2008; Vansteelandt and Joffe, 2014). Under the restriction of effect stationarity (ie, having common blip parameters across the measurement occasions), we can parameterize the blip as a simple function of the history (Vansteelandt and Joffe, 2014, Section 5.1) that is $\gamma_{j}^{*}\left(a_{j},h_{ij};\psi \right)=a_{j}h_{ij}^{⊤}\psi$, where $h_{ij}$, contains a one and potential confounders chosen from the histories. In this formulation, each component of ***ψ*** represents the change in the effect of treatment associated with the corresponding covariate. Under this semiparametric approach, the blip model needs to be correctly specified as a function of the history for consistent estimation of ***ψ***. We construct the proximal blipped-down outcome as $U_{ij}=Y_{ij}-\gamma_{j}^{*}\left(A_{ij},H_{ij};\psi \right)$ at the *j*th occasion. These blipped-down outcomes are a transformation of the observed data such that

$$E\left(U_{ij}|H_{ij}=h_{ij},A_{ij}=a_{j}\right)\;\;=E\left\{Y_{ij}\left({\bar{a}}_{j-1},0\right)|H_{ij}=h_{ij},A_{ij}=a_{j}\right\}.$$

This implies that the variable *U_ij_* has the same mean as the potential outcome under the reference treatment level at occasion *j*. Under the above-mentioned assumptions, the efficient score function (Tsiatis, 2006; Chakraborty and Moodie, 2013) for ***ψ*** is given by

(2) $$S^{eff}(\psi )=\sum_{i=1}^{n}{\left[\frac{\partial \gamma^{*}\left(A_{i},H_{i};\psi \right)}{\partial \psi^{⊤}}-E\left\{\frac{\partial \gamma^{*}\left(A_{i},H_{i};\psi \right)}{\partial \psi^{⊤}}|H_{i}\right\}\right]}^{⊤}\;\;\times Var{\left(U_{i}|H_{i}\right)}^{-1}\left\{U_{i}-E\left(U_{i}|H_{i}\right)\right\},$$

where $A_{i}={\left(A_{i1},\ldots ,A_{iJ}\right)}^{⊤},H_{i}={\left(H_{i1},\ldots ,H_{iJ}\right)}^{⊤}$ is a J×K matrix representing the unit-wise history for the *i*th subject, $U_{i}={\left(U_{i1},\ldots ,U_{iJ}\right)}^{⊤},E\left(U_{i}|H_{i}\right)=H_{i}\delta$ is the treatment-free model, and ***δ*** denotes the corresponding parameters.

We define the whole parameter vector as $\theta ={\left(\delta^{⊤},\psi^{⊤}\right)}^{⊤}$ and denote the corresponding efficient score function by $S^{\text{eff}}(\theta )$ (computational form is provided in Section S1 of the Supplementary Materials). We express the covariance matrix of $U_{i}$ as

$$Var\left(U_{i}|H_{i}\right)=Q_{i}^{1/2}R_{i}(\alpha )Q_{i}^{1/2},$$

where $U_{i}=Y_{i}-A_{i}\cdot H_{i}\psi ,Q_{i}=\sigma^{2}I_{\left(J\right)}$, and $R_{i}(\alpha )$ is the J×J matrix representing the correlations among the blipped-down outcomes of a patient and is defined with parameter *α*. The form of Var(**U***_i_* |**H***_i_*) resembles the working covariance structure used in generalized estimating equations (GEE) introduced by Liang and Zeger (1986). Since **Q***_i_* and **R***_i_* (*α*) are unknown, we replace these quantities in $S^{\text{eff}}(\theta )$ by their estimates. We estimate the parameters *σ*^2^ and *α* using the residual-based moment method under a working correlation structure (corstr). We replace **R***_i_* (*α*) by the estimated correlation matrix $\hat{R}$.

We define $D_{ij}={\left[H_{ij}^{⊤},\left\{A_{ij}-E\left(A_{ij}|H_{ij}\right)\right\}\cdot H_{ij}^{⊤}\right]}^{⊤}$ and $D_{i}={\left(D_{i1},\ldots ,D_{ij}\right)}^{⊤}$ of dimension J×2K for i=1,…,n,j=1,…,J. With $Var\left(U_{i}|H_{i}\right)$ replaced with its estimate, we can write the efficient score as $S^{\text{eff}}(\theta )=\sum_{i=1}^{n}S_{i}^{\text{eff}}(\theta )$, where the contribution in the efficient score from the *i*th subject can be expressed as

$$S_{i}^{\text{eff}}(\theta )=D_{i}^{⊤}Var{\left(U_{i}|H_{i}\right)}^{-1}\left\{U_{i}-E\left(U_{i}|H_{i}\right)\right\}\;\;=D_{i}^{⊤}{\hat{Q}}_{i}^{-1/2}{\hat{R}}^{-1}{\hat{Q}}_{i}^{-1/2}\left\{U_{i}-E\left(U_{i}|H_{i}\right)\right\}.$$

The G-estimates ${\hat{\theta }}_{n}$ are obtained by solving the estimating equations $S^{\text{eff}}(\theta )=0$.

### 2.3 Proposed method (penalized G-estimation)

For simultaneous selection of EMs and estimation of the parameters, we propose a penalized efficient score function using a non-convex smoothly clipped absolute deviation (SCAD) penalty (Fan and Li, 2001). The SCAD penalty achieves 3 desirable properties of variable selection: unbiasedness, sparsity, and continuity (Fan and Li, 2001). Under the assumptions for identifiability of the target parameter ***ψ*** given in Section 2.1, we propose the following penalized efficient score function:

(3) $$S^{P}(\psi )=\sum_{i=1}^{n}{\left[\frac{\partial \gamma^{*}\left(A_{i},H_{i};\psi \right)}{\partial \psi^{⊤}}-E\left\{\frac{\partial \gamma^{*}\left(A_{i},H_{i};\psi \right)}{\partial \psi^{⊤}}|H_{i}\right\}\right]}^{⊤}\;\;\times Var{\left(U_{i}|H_{i}\right)}^{-1}\left\{U_{i}-E\left(U_{i}|H_{i}\right)\right\}\;\;-nq_{\lambda_{n}}(|\psi |)sign(\psi ),$$

where $q_{\lambda_{n}}(|\psi |)={\left\{0,q_{\lambda_{n}}\left(\left|\psi_{1}\right|\right),\ldots ,q_{\lambda_{n}}\left(\left|\psi_{K-1}\right|\right)\right\}}^{⊤}$ and q(.) indicates the first derivative of the SCAD penalty function given by

$$q_{\lambda_{n}}(\psi )=\lambda_{n}\left\{I\left(\psi \leq \lambda_{n}\right)+\frac{{\left(b\lambda_{n}-\psi \right)}_{+}}{(b-1)\lambda_{n}}I\left(\psi >\lambda_{n}\right)\right\}$$

for ψ≥0 and some b>2 with $x_{+}=xI(x>0)$. Fan and Li (2001) suggested to use *b* = 3.7. The amount of shrinkage in estimation is determined by the tuning parameter *λ_n_*.

For the whole parameter vector ***θ***, the proposed penalized efficient score function becomes

$$S^{P}(\theta )=S^{\text{eff}}(\theta )-nq_{\lambda_{n}}(|\theta |)sign(\theta ),$$

where $q_{\lambda_{n}}(|\theta |)={\left\{0^{⊤},q_{\lambda_{n}}\left(\left|\psi^{⊤}\right|\right)\right\}}^{⊤}$. The penalized estimates are obtained by solving the following estimating equations:

(4) $$S^{P}(\theta )=0.$$

Note that we do not penalize the parameters ***δ*** of the treatment-free model and the main effect of the treatment (*ψ*_0_), because our goal is only to identify the EMs.

For solving the penalized efficient estimating equations (4), we use the minorization-maximization (MM) algorithm for non-convex penalty discussed by Hunter and Li (2005). We combine the MM algorithm with the iterative procedure of G-estimation. According to the MM algorithm, the penalized G-estimator $\tilde{\theta }$ approximately satisfies

(5) $$S_{m}^{eff}(\tilde{\theta })-nq_{\lambda_{n}}\left(\left|{\tilde{\theta }}_{m}\right|\right)sign\left({\tilde{\theta }}_{m}\right)\frac{\left|{\tilde{\theta }}_{m}\right|}{\epsilon +\left|{\tilde{\theta }}_{m}\right|}=0$$

for m=1,…2K, where $S_{m}^{\text{eff}}(\tilde{\theta })$ denotes the *m*th element of $S^{\text{eff}}(\tilde{\theta })$ and *ϵ* can be a small number, for example, 10^−6^. If we take a closer look, it is easy to understand that Equations (5) are perturbed versions of the original estimating equations (4). For obtaining the penalized G-estimates, the Newton-Raphson type updating formula at the *t*th iteration directly follows from Equations (5) and is given by

(6) $$\theta^{t+1}=\theta^{t}+{\left\{H_{n}\left(\theta^{t}\right)+nE_{n}\left(\theta^{t}\right)\right\}}^{-1}\left\{S^{eff}\left(\theta^{t}\right)-nE_{n}\left(\theta^{t}\right)\theta^{t}\right\},$$

where

(7) $$H_{n}\left(\theta^{t}\right)=-{\left.E\left(\frac{\partial S^{eff}(\theta )}{\partial \theta^{⊤}}\right)\right|}_{\theta =\theta^{t}}$$

and

(8) $$E_{n}\left(\theta^{t}\right)={\left.diag\left\{0^{⊤},\frac{q_{\lambda_{n}}\left(\left|\psi_{1}\right|\right)}{\epsilon +\left|\psi_{1}\right|},\ldots ,\frac{q_{\lambda_{n}}\left(\left|\psi_{K-1}\right|\right)}{\epsilon +\left|\psi_{K-1}\right|}\right\}\right|}_{\psi =\psi^{t}}.$$

In Equation (6), the derivative of the efficient score, $H_{n}\left(\theta^{t}\right)$, is a 2K×2K-dimensional matrix, and $E_{n}\left(\theta^{t}\right)$ is a 2K×1 dimensional vector. The performance of the penalized estimators relies on the proper choice of tuning parameter. We use a doubly robust information criterion (Bian et al., 2023; Moodie et al., 2023) for selecting the tuning parameter (technical details regarding this criterion are provided in Section S3 of the Supplementary Materials). For a specific corstr, the steps for the whole estimation procedure are summarized in Algorithm 1.

In Algorithm 1, $E\left(A_{i}|H_{i}\right)$ in Step 2 is based on a logistic regression on the pooled data, and the initial estimator ***θ***^up^ in Step 3 is the univariate unpenalized G-estimator. The computational formulas for the method of moments estimators *σ^t^* and *α^t^* of Step 8, $S^{\text{eff}}\left(\theta^{t}\right)$ of Step 10, and DRIC_*λ*_ of Step 16 are provided in Sections S2, S1, and S3 of the Supplementary Materials, respectively. We set *κ* = 10^−6^ in Step 14. Details regarding computation of the correlation matrix ${\hat{R}}_{i}(\alpha )$ in ${\hat{V}}_{i}$ under different structures can be found in Sultana et al. (2023). Note that $\hat{V}i=\hat{V}\forall i$ if J is fixed.

### 2.4 Asymptotic properties

In this section, we study the asymptotic properties of the proposed penalized G-estimator, where we assume a constant cluster size (ie, the same number of sessions for patients).

**Algorithm 1:** Penalized G-estimation algorithm

1:	**procedure** penalizedG (**A, H, Y**, λ, corstr, *κ*)
2:	Compute $\text{E}\left(A_{i}|H_{i}\right)$ for i=1,…,n
3:	$\theta^{\text{up}}\leftarrow {\left\{\sum_{i=1}^{n}D_{i}^{⊤}\left(H_{i}A_{i}\cdot H_{i}\right)\right\}}^{-1}\sum_{i=1}^{n}D_{i}^{⊤}Y_{i}$
4:	**for all** $\lambda \in \left(\lambda_{\max},\ldots ,\lambda_{\min}\right)$ **do**
5:	Initialize: $t=0,\theta^{0}\leftarrow \theta^{up}$
6:	**repeat**
7:	$e_{i}\leftarrow Y_{i}-\left(H_{i}A_{i}\cdot H_{i}\right)\theta^{t}$ for i=1,…,n
8:	Compute $\sigma^{t}$ and $\alpha^{t}$ under corstr
9:	Compute ${\hat{V}}_{i}$ according to corstr for i=1,…,n
10:	Compute $S^{\text{eff}}\left(\theta^{t}\right)$
11:	Compute $H_{n}\left(\theta^{t}\right)$ and $E_{n}\left(\theta^{t}\right)$ using (7) and (8).
12:	Update $\theta^{t}$ according to (6) and obtain $\theta^{t+1}$
13:	t←t+1
14:	**until** $\|\theta^{t}-\theta^{t-1}\|<\kappa$
15:	${\tilde{\theta }}_{\lambda }\leftarrow \theta^{t},{\tilde{\sigma }}_{\lambda }\leftarrow \sigma^{t},$ and ${\tilde{\alpha }}_{\lambda }\leftarrow \alpha^{t}$
16:	Compute ${\text{DRIC}}_{\lambda }$
17:	**end for**
18:	$\tilde{\theta }\leftarrow {\tilde{\theta }}_{\lambda }^{*}\;\text{s.t.}\;{\tilde{\theta }}_{\lambda }^{*}$ corresponds to the minimum of $DRIC_{\lambda }$
19:	**return** $\tilde{\theta }$
20:	**end procedure**

Let $\theta_{0}={\left(\delta_{0}^{⊤},\psi_{0}^{⊤}\right)}^{⊤}$ have dimension 2*K*, where ***δ***_0_ is the *K*-dimensional vector of population parameters corresponding to the assumed treatment-free model and ***ψ***_0_ is the *K*-dimensional vector of true coefficients of the blip model. Let $B_{1}=\{1,\ldots ,K+1\}$ be the set representing the indices of the unpenalized main effects (***δ***_0_ and *ψ*_00_) and $B_{2}=\left\{m:\psi_{0m}\neq \right.0;m=K+2,\ldots ,2K\}$ be the index set for the variables that are true EMs of the treatment within this model. Let $B=B_{1}\cup B_{2}$ and *s* denote the cardinality of the set *B*. Then, let *B^c^* represent the indices of the zero coefficients in ***ψ***_0_, that is, the indices of the variables that do not modify the effect of the treatment.

Let $\hat{R}$ introduced in Section 2.2 satisfies $\left\|{\hat{R}}^{-1}-{\bar{R}}^{-1}\right\|=O_{p}(\sqrt{1/n})$, where $\bar{R}=E(\hat{R})$ is a constant positive definite matrix with eigenvalues bounded away from zero and +∞. Note that $\|X\|={\left\{trace\left({XX}^{⊤}\right)\right\}}^{1/2}$ denotes the Frobenius norm of the matrix **X**. Define 2 versions of the expected information as

$$H(\theta )=E\left\{-\partial S_{i}^{\text{eff}}(\theta )/\partial \theta^{⊤}\right\}\;\;=D_{i}^{⊤}Q_{i}^{-1/2}{\bar{R}}^{-1}Q_{i}^{-1/2}\left(H_{i}A_{i}\cdot H_{i}\right),\;I(\theta )=E\left\{S_{i}^{\text{eff}}(\theta )S_{i}^{\text{eff}}(\theta {)}^{⊤}\right\}\;\;=D_{i}^{⊤}Q_{i}^{-1/2}{\bar{R}}^{-1}R_{0}{\bar{R}}^{-1}Q_{i}^{-1/2}D_{i},$$

where **R**_0_ is the true correlation matrix. Details regarding the expressions of these information matrices can be found in Balan and Schiopu-Kratina (2005), where the authors presented a rigorous asymptotic theory for generalized estimating equations.

According to Johnson et al. (2008), for establishing the asymptotic properties, the required conditions on the efficient score function and the penalty function are as follows:

(C.a) There exists a non-singular matrix **H** (***θ***_0_) such that for any given constant *Z*,
$$\underset{\left|\theta -\theta_{0}\right|\leq Zn^{-1/2}}{sup}|n^{-1/2}S^{\text{eff}}(\theta )-n^{-1/2}S^{\text{eff}}\left(\theta_{0}\right)\;-n^{1/2}H\left(\theta_{0}\right)\left(\theta -\theta_{0}\right)|=o_{p}\left(1\right).$$
  Moreover,
$$n^{-1/2}S^{\text{eff}}\left(\theta_{0}\right)\overset{d}{\to }N\left(0,I\left(\theta_{0}\right)\right).$$
(C.b) The derivative *q_λ_n__* (·) of the penalty function has the following properties:
  (C.b.1) For non-zero fixed $\theta ,lim\;n^{1/2}q_{\lambda_{n}}(|\theta |)=0$ and $lim\;q_{\lambda_{n}}^{\prime }(|\theta |)=0$.
  (C.b.2) For any $Z>0,lim\;\sqrt{n}{inf}_{|\theta |\leq Zn^{-1/2}}q_{\lambda_{n}}(|\theta |)\to \infty$,

where $q_{\lambda_{n}}^{\prime }(\cdot )$ denotes the derivative of $q_{\lambda_{n}}(\cdot )$. Under the conditions C.a, C.b, and the regularity conditions C1-C7 (provided in Section S4 of the Supplementary Materials) applicable for GEE, there exists a $\sqrt{n}$-consistent approximate zero crossing of $S^{P}(\theta )$ indicating ${\tilde{\theta }}_{n}=\theta_{0}+O_{p}\left(n^{-1/2}\right)$, such that ${\tilde{\theta }}_{n}$ is an approximate zero crossing of **S***^P^* (***θ***). The proof is analogous to what is presented in Johnson et al. (2008). Also, the proposed estimator enjoys the oracle property, which means that it estimates true zero coefficients as zero with probability approaching one and the true non-zero coefficients as efficiently as if the true model is known in advance. This property is stated in the next 2 theorems.

**Theorem 1** (**Correct Sparsity**) *Let* $\theta^{B}$ *represent the elements of **θ** whose indices belong to B. Under the required conditions (C.a, C.b, and C1-C7), if λ_n_* → 0 *and* $\sqrt{n}\lambda_{n}\to \infty$ *as* n→∞, *then there exists an approximate penalized G-estimator* ${\tilde{\theta }}_{n}=\left\{{\tilde{\theta }}_{n}^{B},{\tilde{\theta }}_{n}^{B^{c}}\right\}$ *such that*

(9) $$P\left({\tilde{\theta }}_{n}^{B^{c}}=0\right)\to 1.$$

This theorem establishes the correct sparsity of the penalized G-estimator. The proof of Theorem 1 is provided in Section S5 of the Supplementary Materials.

**Theorem 2** (**Asymptotic Normality**) *Let* **D***_i, B_ represent the elements of the matrix* **D***_i_ that correspond to the coefficients **θ**^B^. Under the required conditions (C.a, C.b, and C1-C7), if* $\lambda_{n}\to 0$ *and* $\sqrt{n}\lambda_{n}\to \infty$ *as* n→∞, *the penalized G-estimator* ${\tilde{\theta }}_{n}^{B}$ *satisfies*

$$n^{1/2}\left\{H_{B}\left(\theta_{0}\right)+W_{n}\left(\theta_{0}^{B}\right)\right\}\left[{\tilde{\theta }}_{n}^{B}-\theta_{0}^{B}\right.\;\;\left.+{\left\{H_{B}\left(\theta_{0}\right)+W_{n}\left(\theta_{0}^{B}\right)\right\}}^{-1}b_{n}\right]\overset{d}{\to }N\left(0,I_{B}\left(\theta_{0}\right)\right),$$

*where* $H_{B}\left(\theta_{0}\right),W_{n}\left(\theta_{0}^{B}\right)$, *and* $I_{B}\left(\theta_{0}\right)$ *are the s × s submatrices of $H\left(\theta_{0}\right),W_{n}\left(\theta_{0}\right)$, and* $I\left(\theta_{0}\right)$ *that correspond to the indices in B,*

$$W_{n}\left(\theta_{0}\right)=\mathrm{diag}\left\{0^{⊤},-q_{\lambda_{n}}^{\prime }\left(\left|\psi_{01}\right|\right)\mathrm{sign}\left(\psi_{01}\right),\ldots ,\right.\;\;\left.-q_{\lambda_{n}}^{\prime }\left(\left|\psi_{0(K-1)}\right|\right)\mathrm{sign}\left(\psi_{0(K-1)}\right)\right\},$$

*and* $b_{n}=-q_{\lambda_{n}}\left(\left|\theta_{0}^{B}\right|\right)\mathrm{sign}\left(\theta_{0}^{B}\right)$.

This theorem establishes the asymptotic normality of the penalized G-estimator ${\tilde{\theta }}_{n}^{B}$. The proof of Theorem 2 is provided in Section S6 of the Supplementary Materials.

**Corollary 1 (Double Robustness)** *If the consistency and the sequential ignorability assumptions in* Section 2.1 *are correct, the penalized G-estimator* ${\tilde{\psi }}_{n}$ *is doubly robust, that is*, ${\tilde{\psi }}_{n}$ *is a consistent estimator for* ψ *if either the exposure model or the treatment-free model is correctly specified given that the candidate set for the blip function contains the true blip model. It follows from Theorems 1 and 2 that penalized G-estimator inherits the double-robustness property of G-estimator*.

Although the asymptotic properties are derived under the same number of measurements per subject for the ease of proof, our theory and related estimator can handle unequal numbers of measurements. In the simulation study of Section 3.1, we numerically verified that the results of the theorems also hold for non-fixed sessions.

An asymptotic sandwich covariance estimator for ${\tilde{\theta }}_{n}^{B}$ directly follows from Theorem 2 and is given by

(10) $$Cov\left({\tilde{\theta }}_{n}^{B}\right)=n^{-1}{\left\{H_{B}\left(\theta_{0}\right)+W_{n}\left(\theta_{0}^{B}\right)\right\}}^{-1}\;\;\times I_{B}\left(\theta_{0}\right){\left\{H_{B}\left(\theta_{0}\right)+W_{n}\left(\theta_{0}^{B}\right)\right\}}^{-1}.$$

We can consistently estimate the variance in (10) by replacing the quantities in it by their empirical estimates as follows:

$$\hat{Cov}\left({\tilde{\theta }}_{n}^{B}\right)=n^{-1}{\left\{{\hat{H}}_{B}\left({\tilde{\theta }}_{n}\right)+W_{n}\left({\tilde{\theta }}_{n}^{B}\right)\right\}}^{-1}\;\;\times {\hat{I}}_{B}\left({\tilde{\theta }}_{n}\right){\left\{{\hat{H}}_{B}\left({\tilde{\theta }}_{n}\right)+W_{n}\left({\tilde{\theta }}_{n}^{B}\right)\right\}}^{-1},$$

where ${\hat{I}}_{B}\left({\tilde{\theta }}_{n}\right)=n^{-1}\sum_{i=1}^{n}S_{i}^{\text{eff}}\left({\tilde{\theta }}_{n}^{B}\right){\left\{S_{i}^{\text{eff}}\left({\tilde{\theta }}_{n}^{B}\right)\right\}}^{⊤}$ and ${\hat{H}}_{B}\left({\tilde{\theta }}_{n}\right)=-{\left.n^{-1}\sum_{i=1}^{n}\frac{\partial S_{i}^{\text{eff}}(\theta )}{\partial \theta^{⊤}}\right|}_{\theta ={\tilde{\theta }}_{n}^{B}}$.

**Remark 1** *Theorem 2 requires the propensity score (ie, treatment model parameters) to be known. Because the propensity score is unknown and must be estimated using the observed data, the variance of our estimator should be calculated as shown in* Section S7 *of the* Supplementary Materials. *This section shows how we can modify our estimating equation to account for the contribution of the estimated propensity score in the asymptotic variance of our estimator. This approach was adapted from* Robins (1992).

**Remark 2** *The sandwich variance estimator is valid if we have infinitely large samples. However, infinite samples, the uncertainty in the selection of EMs invalidates the post-selection inference based on this sandwich estimator. Under data-driven selection, we tend to favor models with strong effects with an associated cost of inflated type I errors* (Zhao et al., 2022*). Appropriate statistical approach is required for a valid post-selection inference for the penalized G-estimation infinite samples.*

## 3 SIMULATION STUDY

In order to evaluate the performance of the proposed estimator in finite samples, we conducted 2 simulation studies. The first evaluates the oracle property from Theorems 1 and 2; the setup and results are presented in Section 3.1. The second simulation study in Section 3.2 verifies the double-robustness property of our estimator.

### 3.1 Evaluation of the oracle property in finite samples

To generate the data for j th session (j=1,…,J) of each subject, we generated 2 baseline confounders as $L^{\left(1\right)}∼\mathrm{Bernoulli}(0.5)$ and $L^{\left(2\right)}∼N(0,1)$, and the time-varying confounders and noise covariates as $L_{j}^{\left(3\right)},\ldots ,L_{j}^{\left(6\right)},X_{j}^{\left(1\right)},\ldots ,X_{j}^{\left(10\right)}∼MVN_{14}\left({\left(\mu_{L,j}^{⊤},\mu_{X,j}^{⊤}\right)}^{⊤},V\right)$, where $\mu_{L,j}^{\left(k\right)}=0.3l_{j-1}^{\left(k\right)}+0.3a_{j-1}$ for k=3,4,5, and 6, and $\mu_{X,j}^{\left(r\right)}=0.5x_{j-1}^{\left(r\right)}$ for r=1,…,10. Note that we define $l_{0}^{\left(k\right)}=0$ for any $k,a_{0}=0$, and $x_{0}^{\left(r\right)}=0$ for any r. The covariance matrix V has (r,s) th element equal to $\rho^{\left|r-s\right|}$ for r,s=1,…,14. We generated the binary exposure according to the probability

(11) $$P\left(A_{j}=1|H_{j}\right)\;\;=\frac{exp\;\left(\beta_{0}+\beta_{1}l^{\left(1\right)}+\beta_{2}l^{\left(2\right)}+\sum_{m=3}^{6}\beta_{m}l_{j}^{\left(m\right)}+\beta_{7}a_{j-1}\right)}{1+exp\;\left(\beta_{0}+\beta_{1}l^{\left(1\right)}+\beta_{2}l^{\left(2\right)}+\sum_{m=3}^{6}\beta_{m}l_{j}^{\left(m\right)}+\beta_{7}a_{j-1}\right)}$$

We then generated a vector of correlated errors $\epsilon ∼N_{J}(0,\Sigma )$, where $\Sigma =\sigma_{\epsilon }^{2}R$ is the variance-covariance matrix, and **R** is the *J* × *J* correlation matrix defined with parameter *α* according to a “exchangeable” corstr. Finally, we constructed the outcome as

$$y_{j}=\mu_{j}\left(h_{j};\delta \right)+\gamma_{j}^{*}\left(a_{j},h_{j};\psi \right)+\epsilon_{j},$$

where $\mu_{j}\left(h_{j};\delta \right)=\delta_{0}+\delta_{1}l^{\left(1\right)}+\delta_{2}l^{\left(2\right)}+\sum_{m=3}^{6}\delta_{m}l_{j}^{\left(m\right)}+\delta_{7}exp\left(l_{j}^{\left(5\right)}\right)+\delta_{8}a_{j-1}$ and $\gamma_{j}^{*}\left(a_{j},h_{j};\psi \right)=\left(\psi_{0}+\psi_{1}l^{\left(1\right)}+\right.\left.\psi_{2}l^{\left(2\right)}+\sum_{m=3}^{6}\psi_{m}l_{j}^{\left(m\right)}+\psi_{7}a_{j-1}\right)a_{j}$. Let $\beta ={\left(\beta_{0},\ldots ,\beta_{7}\right)}^{⊤}$, $\delta ={\left(\delta_{0},\ldots ,\delta_{8}\right)}^{⊤}$, and $\psi ={\left(\psi_{0},\ldots ,\psi_{7}\right)}^{⊤}$. We fixed $\beta ={\left(0,1,1,1,1,1,1,-0.8\right)}^{⊤},\delta ={\left(1,-1,1,1,1,1,1,1,1\right)}^{⊤}$, and we considered 2 different models for the blip;

- **Setting 1** (stronger effect modification): ***ψ*** = (1, −2.5, 1.5, 1.5, 1.5, 1.5, 0, 2)^⊤^
- **Setting 2** (weaker effect modification): ***ψ*** = (1, −2, 1, 0.75, 0.9, 1.2, 0, 1.8)^⊤^.

Note that none of the *X* ’s were used to generate $\mu_{j}\left(h_{j};\delta \right)$ and $\gamma_{j}^{*}\left(a_{j},h_{j};\psi \right)$. We considered *n* = 200, *ρ* = 0 versus 0.25, $\sigma_{\epsilon }^{2}=1$ versus 4, and *α* = 0.8. All of the confounders and noise covariates were considered candidates for effect modification. We performed the proposed penalized G-estimation using 3 different working corstrs: independent (Indep), exchangeable (Exch), and unstructured (UN). In all the scenarios, we compare the estimates under a misspecified treatment-free model. We consider an EM to be eliminated by the method if |effect modification| < 0.001. For evaluating the model selection performance, we considered the rate of false negatives (FNs) (ie, the proportion of times the method eliminated at least one true EM), the rate of false positives (FPs) (ie, the proportion of times the method selected at least one covariate that is not a true EM), the rate of exact selections (EXACT) (ie, proportion of times the method selected the true set of EMs), and the average number of false positives (AFPs) (ie, the average number of variables selected by the method that do not belong to the set of true EMs). The performance measures for different settings were calculated from 500 independent simulations and are shown in Table 1.

The selection rates of the exact model are above 90% for most cases when the error variance is 1. When the error variance was increased, the exact selection rate decreased and the FN rate increased. When the outcomes were more highly correlated and the candidate variables were autocorrelated among themselves, accounting for correlation among the observed outcomes within a patient produced a clear advantage over the independence assumption. In this case, the FN and FP rates produced by the working independent structure are higher than the other 2 structures.

Simulations were also conducted for a larger sample size by setting *n* = 500 and 2 different values for the correlation parameter by setting α = 0.4 and 0.8 indicating low and high correlation among the repeated outcomes, respectively; the results are presented in Table 2. Under the larger sample size (*n* = 500) with all other parameters being the same, we see an improved performance of the proposed method (compare the second row of Table 1 to the bottom row of Table 2), supporting the result of Theorem 1.

We also compared the performance of the penalized estimator with the oracle estimator (estimator obtained from the true blip model but with the same misspecification in the treatment-free part) under each of the working corstrs. In Table 3, we reported the relative bias, the empirical root-mean-squared error (SE1), and the square root of the average of sandwich variance estimates (SE2) under different working corstrs. From the results, we see that the usage of non-independent corstrs resulted in more efficient estimation of the blip parameters. Moreover, the efficiency of the penalized estimates is comparable to that of the oracle estimates, which also verifies the property stated in Theorem 2.

We performed additional simulations allowing for the number of visits (or measurement occasions) for each subject to be random; the results are provided in Table S2 of the Supplementary Materials. We also considered alternative choices for the exposure-generating model, for example, when the exposure selection probability depends on past outcomes, and for the outcome-generating model where past outcomes affect future outcomes. The results are provided in Tables S3 and S4, respectively, in the Supplementary Materials. We performed additional simulations in a high-dimensional setup considering 2 different dimensions of covariates: *K* = 50 versus 100; the results are provided in Table S5 of the Supplementary Materials. We also compared our penalized G-estimator with an existing alternative, which is the proximal treatment effect estimator of Boruvka et al. (2018), under the full model as Boruvka’s method does not allow for variable selection; the results are presented in Table S6 of the Supplementary Materials.

### 3.2 Verification of the double-robustness property

The double robustness of the proposed penalized G-estimator was verified via a simulation experiment with *n* = 500 and *J* = 6 for all subjects. We considered estimation under 4 different scenarios: **Scenario 1:** The treatment model is correct, and the treatment-free model is misspecified; **Scenario 2:** The treatment model is incorrect, and the treatment-free model is correct; **Scenario 3:** Both models are correct; and **Scenario 4:** Both models are misspecified. For data generation, we set ***β*** = (0, 1, 1, 1, 1, 1, 1, −0.8)^⊤^, ***δ*** = (1, −1, 1, 1, 1, 1, 1, −0.8, 1)^⊤^, and ***ψ*** = (1, −2.5, 1.5, 1.5, 1.5, 1.5, 0, 2)^⊤^. This parameter setup was chosen because, with this setup under Scenario 4, where both models are misspecified, the coefficient estimate of at least one true EM was estimated to be around zero. This lets us verify the double-robustness property of the proposed estimator in terms of EM selection consistency. Data were generated under an exchangeable structure with *α* = 0.8, error variance $\sigma_{\epsilon }^{2}=1$, and autocorrelation coefficient *ρ* = 0.25. For each generated data set, we performed the proposed estimation under each of the 4 scenarios and 3 working corstrs. For estimation under Scenario 1, we estimated the propensity score using the true exposure model but excluded the exp(*L*^(5)^) predictor from the treatment-free model. In Scenario 2, we included the exp(*L*^(5)^) predictor in the treatment-free model but used a null model for estimating the propensity score, that is, we assigned the overall empirical proportion of exposure as the probability of being exposed at each measurement occasion. In Scenario 3, we estimated the propensity score using the true exposure model and also included exp(*L*^(5)^) as a covariate in the treatment-free model. Finally, in Scenario 4, we considered a null model for estimating the propensity score and also excluded exp(*L*^(5)^) as a covariate in the treatment-free part. We report the percentage selection for each of the candidate EMs with rates of FN, FP, and exact selection in Table 4.

When both the treatment and the treatment-free models were incorrect (Scenario 4), the proposed method did not select the true EM, *L*^(5)^, in more than 70% of the simulations irrespective of what the corstr was. Misspecification of the functional form of this EM in the treatment-free part caused a biased estimation of its coefficient in the blip model. The direction of this bias was toward the null, and as a result, we observed the exclusion of this EM in the majority of the simulations. But when at least one of those models was correct (Scenarios 1-3) the proposed method performed well in identifying the true EMs under the non-independent corstrs. When both models were correct (Scenario 3), the FP rates were higher under the independent structure when compared with exchangeable and unstructured corstrs.

## 4 APPLICATION TO THE HEMODIAFILTRATION STUDY

Our study data arise from an open cohort of patients undergoing chronic hemodiafiltration at CHUM and CED. Hemodiafiltration was considered as chronic if there were at least 28 consecutive sessions. The cohort start date for each patient was their first dialysis session on or after March 1, 2017; the cohort end date was December 1, 2021. The primary data include information from a total of 474 patients who underwent 170761 dialysis sessions. The following information was extracted from hospital databases for each session: drugs (BDM), laboratory results (CERNER), procedures related to dialysis venous access (Radimage), and dialysis-related variables and dialysis-related drugs (EuCliD-NephroCare). Comorbidities that are potential confounders were obtained from the *Maintenance et exploitation des données pour l’étude de la clientèle hospitalière* (MEDECHO) database. Confounders and potential EMs that we considered in the analysis are previous outcome (24 L or less = 1 versus more than 24 L = 0), hemoglobin, albumin, dalteparin, access (fistula = 0, catheter = 1), catheter change, age, sex, and the components of the Charlson Comorbidity Index (hypertension, diabetes, peripheral vascular disease, congestive heart failure, cardiac arrhythmia, acute myocardial infarction, chronic pulmonary disease, liver disease, valvular disease, cancer, metastatic cancer, cerebrovascular disease, dementia, hemiplegia, and rheumatic disease). The exposure is the dialysis facility and coded as 1 if the treatment location was CHUM and 0 if the location was CED. We dichotomized the past outcome when used as a confounder because the decision to have the next session at the CHUM is partially based on the success of the previous session, which is typically defined by clinicians as volume > 24 L (Chapdelaine et al., 2015), and when included as a potential EM for the ease of clinical interpretation. Some descriptive statistics of the data are presented in Section S9 of the Supplementary Materials.

We included the first 6 consecutive sessions of post-dilution hemodiafiltration for each of the patients in our analysis. We estimated the propensity scores using a logistic regression of exposure conditional on all of the potential confounders using the pooled data set. We performed the proposed penalized G-estimation considering 4 different corstrs: independent, exchangeable, autoregressive of order one (AR1), and unstructured. For *j*th session, for example, under the AR1 corstr, the selected blip model (with adjustment for all potential confounders in the treatment-free part) is

$$\gamma_{j}\left(a_{j},h_{j};\psi \right)=\left(\psi_{0}+\psi_{1}\times {\text{Cancer}}_{j}\right){\text{CHUM}}_{j}$$

for *j* = 1, 2, …, 6. The estimates of the blip parameters are given in Table 5 with their corresponding sandwich standard error estimates.

Under the AR1 corstr, cancer was selected by the method, indicating that the effect of dialysis facility on the convection volume differs by the cancer status of the patient. If we interpret these results, we can say that among the patients who did not have cancer, the mean convection volume was 1.85 L lower at CHUM when compared with CED at fixed levels of all other confounders. But the mean convection volume was 3.89 – 1.85 = 2.04 L higher at the CHUM for cancer patients indicating that cancer patients with the same measurements on the adjusted confounders had better hemodiafiltration outcomes at the CHUM.

## 5 DISCUSSION

In this paper, we proposed a penalization in the G-estimation for evaluating the causal effect of a time-varying exposure with automatic EM selection with longitudinal observational data and a proximal outcome of interest. We applied this method to investigate if the effect of dialysis facility on the dialysis outcome (convection volume) differs by the demographic and clinical characteristics and comorbidity status of the patients with end-stage renal disease. Our findings suggest that while the CED produced better hemodiafiltration outcomes overall, cancer patients with similar measured characteristics might have had better outcomes at the CHUM compared to the CED.

In the simulation study, our method performed well in selecting the true blip model from a set of candidate models, even when we assumed no correlation between the outcomes from the same patient. However, when we used the correct corstr, the method produced better estimates of the target (blip) parameters than a working independence model. Although we considered different non-independent corstrs in the simulation studies and in the data application, it is possible to choose an appropriate corstr using the data (Jaman et al., 2016; Inan et al., 2019; Sultana et al., 2023). The selection rates of the true blip model were good in the majority of our simulation scenarios. However, these rates depend on the magnitude of effect modification and the signal-to-noise ratio. A challenge still remains in the identification of weak EMs, and this would be an interesting topic for future research. Researchers may wish to study the article by Gunter et al. (2011), where the authors discussed a ranking procedure for selecting EMs that are weaker but important for decision-making.

Other limitations of our method include the computational burden that may arise when subjects contribute a large number of observations. We also did not address informative censoring in this paper, but this is a possibility for a future extension. Although we did not perform variable selection for the treatment model in our hemodiafiltration application, such selection may be necessary in practice, especially in a high-dimensional setup (see the work by Shortreed and Ertefaie (2017) for related method).

## Supplementary Material

Supplementary Material

Codes

Supplementary material is available at *Biometrics* online.

A Web Appendix containing the additional details for Sections 2.2 and 2.3, the regularity conditions, the proofs of Theorems 1 and 2, and the computation of sandwich variance estimator in Section 2.4, the additional simulation results of Section 3.1, the details of hemodiafiltration data of Section 4, and the R-codes for reproducing our results are available with this paper at the Biometrics website on Oxford Academic. An R-package for implementing our method is available at (https://github.com/ajmeryjaman/penalizedG/).

## Figures and Tables

**TABLE 1:** Model selection performance of the penalized G-estimator for data generated with *n* = 200, *J* = 6 for all *i*, an exchangeable correlation structure and *α* = 0. 8 under varying values of the error variance $\sigma_{\epsilon }^{2}$ and varying degrees of autocorrelation between the effect modifiers and noise covariates.

Autocorr coeff (*ρ*)	Working correlation	Setting 1 (stronger EM)				Setting 2 (weaker EM)			

		FN	FP	EXACT	AFP	FN	FP	EXACT	AFP
		$\sigma_{\epsilon }^{2}=1$				$\sigma_{\epsilon }^{2}=1$			
1	Indep	2.0	1.4	96.6	0.01	9.2	3.6	87.8	0.04
	**Exch**	1.6	1.4	**97.0**	0.01	8.8	2.8	**88.8**	0.03
	UN	1.4	1.8	96.8	0.02	10.0	2.6	87.4	0.03
0.25	Indep	2.2	6.0	91.8	0.07	3.6	6.6	90.2	0.07
	**Exch**	1.6	5.6	**92.8**	0.06	2.4	5.8	**92.2**	0.06
	UN	1.0	4.8	94.2	0.06	2.0	3.8	94.4	0.04
		$\sigma_{\epsilon }^{2}=4$				$\sigma_{\epsilon }^{2}=4$			
0	Indep	14.8	2.4	83.0	0.02	44.6	1.4	54.0	0.02
	**Exch**	14.2	1.0	**84.8**	0.01	42.4	0.4	**57.2**	0.00
	UN	14.6	0.0	85.4	0.00	45.8	0.2	54.2	0.00
0.25	Indep	11.8	3.8	84.4	0.04	28.2	3.4	69.0	0.03
	**Exch**	10.6	0.6	**88.8**	0.01	25.4	1.6	**73.2**	0.02
	UN	9.4	0.6	90.0	0.01	24.8	1.6	74.0	0.02

Results are obtained with a misspecified treatment-free model from 500 independent simulations for Setting 1 and Setting 2. The bold values represent results under the true correlation structure used for data generation. FN: % of false negatives, FP: % of false positives, EXACT: % of exact selections, AFP: average false positives, EM: effect modifier, Indep: independent, Exch: exchangeable, UN: unstructured.

**TABLE 2:** Model selection performance of the penalized G-estimator for data generated with *n* = 500, *J* = 6 for all *i*, an exchangeable correlation structure among the repeated outcomes with varying value of correlation parameter *α*, an autocorrelation coefficient *ρ* = 0.25 deciding the correlation among the EM’s and noise covariates, and error variance $\sigma_{\epsilon }^{2}=1$.

*α*	Working correlation	Setting 1 (stronger EM)				Setting 2 (weaker EM)			

		FN	FP	EXACT	AFP	FN	FP	EXACT	AFP
0.4	Indep	0.4	7.2	92.4	0.08	0.0	5.4	94.6	0.05
	**Exch**	0.4	7.2	**92.4**	0.08	0.0	5.8	**94.2**	0.06
	UN	0.4	5.6	94.0	0.06	0.0	4.6	95.4	0.05
0.8	Indep	0.0	5.8	94.2	0.06	0.2	8.8	91.0	0.09
	**Exch**	0.0	5.2	**94.8**	0.06	0.2	6.6	**93.2**	0.07
	UN	0.0	4.6	95.4	0.05	0.0	6.4	93.6	0.07

Results are obtained with a misspecified treatment-free model from 500 independent simulations for Setting 1 and Setting 2. The bold values represent results under the true correlation structure used for data generation. FN: % of false negatives, FP: % of false positives, EXACT: % of exact selections, AFP: average false positives, EM: effect modifier, Indep: independent, Exch: exchangeable, UN: unstructured.

**TABLE 3:** Relative bias (%), empirical (SE1), and sandwich (SE2) estimates of the standard errors for the penalized and the oracle estimators calculated from 500 simulations for *n* = 500, autocorrelation coefficient *ρ* = 0. 25, error variance $\sigma_{\epsilon }^{2}=1$, and *α* = 0. 8 under the 2 settings.

Working model	Penalized								Oracle						

	Stats	*ψ*_0_	*ψ*_1_	*ψ*_2_	*ψ*_3_	*ψ*_4_	*ψ*_5_	*ψ*_7_	*ψ*_0_	*ψ*_1_	*ψ*_2_	*ψ*_3_	*ψ*_4_	*ψ*_5_	*ψ*_7_
Setting 1 (Stronger EM)															
Ind	r.bias	1.32	−1.04	0.05	0.00	0.40	0.06	0.21	1.37	−0.94	0.13	0.18	0.23	0.11	0.04
Ind	SE1	0.16	0.19	0.11	0.11	0.11	0.18	0.19	0.16	0.19	0.11	0.11	0.11	0.18	0.19
Ind	SE2	0.16	0.19	0.11	0.10	0.11	0.18	0.20	0.16	0.19	0.11	0.10	0.11	0.18	0.20
Exch	r.bias	1.13	−1.11	0.01	0.06	0.44	1.30	0.24	1.17	−1.03	0.16	0.21	0.30	1.35	0.03
Exch	SE1	0.14	0.18	0.10	0.10	0.10	0.17	0.17	0.14	0.18	0.10	0.10	0.10	0.17	0.17
Exch	SE2	0.14	0.18	0.10	0.10	0.10	0.17	0.18	0.14	0.18	0.10	0.10	0.10	0.17	0.18
UN	r.bias	1.00	−1.02	0.05	0.01	0.47	1.76	0.10	1.02	−0.95	0.17	0.11	0.35	1.80	0.07
UN	SE1	0.15	0.19	0.11	0.10	0.10	0.17	0.18	0.15	0.19	0.11	0.10	0.10	0.17	0.18
UN	SE2	0.14	0.18	0.10	0.10	0.10	0.17	0.19	0.14	0.18	0.10	0.10	0.10	0.17	0.19
Setting 2 (Weaker EM)															
Ind	r.bias	0.53	−1.01	0.84	1.97	1.59	0.23	2.23	0.47	−0.78	0.44	1.40	1.10	0.31	1.80
Ind	SE1	0.15	0.20	0.11	0.11	0.11	0.19	0.21	0.15	0.20	0.11	0.11	0.11	0.19	0.21
Ind	SE2	0.16	0.19	0.11	0.11	0.11	0.18	0.20	0.16	0.19	0.11	0.11	0.11	0.18	0.20
Exch	r.bias	0.19	−1.09	0.69	1.69	1.17	1.58	1.76	0.15	−0.94	0.39	1.30	0.81	1.62	1.44
Exch	SE1	0.14	0.19	0.11	0.10	0.11	0.18	0.19	0.14	0.19	0.11	0.10	0.10	0.18	0.19
Exch	SE2	0.14	0.18	0.10	0.10	0.10	0.17	0.18	0.14	0.18	0.10	0.10	0.10	0.17	0.18
UN	r.bias	0.61	−0.93	0.85	1.46	1.13	2.28	1.82	0.57	−0.76	0.53	1.04	0.75	2.38	1.49
UN	SE1	0.14	0.20	0.11	0.10	0.11	0.18	0.20	0.14	0.20	0.11	0.10	0.11	0.18	0.20
UN	SE2	0.14	0.18	0.10	0.10	0.10	0.17	0.19	0.14	0.18	0.10	0.10	0.10	0.17	0.19

Relative bias $(\text{r.bias})=100\times ({\tilde{\psi }}_{n}-\psi_{0})/\psi_{0}$, SE1: the empirical root-mean-squared error (MSE), SE2: the square root of the average of sandwich variance estimates, Ind: independent, Exch: exchangeable, UN: unstructured.

**TABLE 4:** Variable selection rates (%) from the penalized G-estimation under different scenarios with different working correlation structures.

	Scenario 1			Scenario 2			Scenario 3			Scenario 4		
	Indep	Exch	UN	Indep	Exch	UN	Indep	Exch	UN	Indep	Exch	UN
*A* × *L*^(1)^	100	100	100	100	100	100	100	100	100	100	100	100
*A* × *L*^(2)^	100	100	100	100	100	100	100	100	100	100	100	100
*A* × *L*^(3)^	100	100	100	100	100	100	100	100	100	100	100	100
*A* × *L*^(4)^	100	100	100	100	100	100	100	100	100	100	100	100
*A* × *L*^(5)^	100	100	100	100	100	100	100	100	100	6.6	7.0	8.2
*A* × *L*^(6)^	5.8	4.4	3.6	0.0	0.0	0.0	2.2	0.0	0.0	0.2	0.0	0.0
*A* × exp (*L*^(5)^)	–	–	–	0.0	0.6	0.4	3.2	0.0	0.0	–	–	–
*A* × *A*_Lag1_	100	100	100	100	100	100	100	100	100	100	100	100
*A* ×*X*^(1)^	0.0	0.2	0.2	0.2	0.0	0.0	2.0	0.0	0.0	0.2	0.0	0.0
*A* × *X*^(2)^	0.4	0.2	0.0	0.0	0.0	0.0	1.2	0.0	0.0	0.0	0.0	0.0
*A* ×*X*^(3)^	0.2	0.4	0.4	0.2	0.0	0.0	2.2	0.0	0.0	0.2	0.0	0.0
*A* × *X*^(4)^	0.2	0.2	0.2	0.0	0.0	0.0	2.8	0.0	0.0	0.4	0.0	0.0
*A* ×*X*^(5)^	0.0	0.2	0.4	0.0	0.0	0.0	1.6	0.0	0.0	0.0	0.0	0.0
*A* ×*X*^(6)^	0.0	0.2	0.4	0.0	0.0	0.0	2.2	0.0	0.0	0.0	0.0	0.0
*A* × *X*^(7)^	0.0	0.0	0.0	0.0	0.0	0.0	2.2	0.0	0.0	0.0	0.0	0.0
*A* ×*X*^(8)^	0.4	0.4	0.4	0.2	0.0	0.0	2.6	0.0	0.0	0.0	0.0	0.0
*A* ×*X*^(9)^	0.0	0.0	0.2	0.0	0.0	0.0	3.0	0.0	0.0	0.2	0.0	0.0
*A* ×*X*^(10)^	0.2	0.2	0.8	0.2	0.0	0.0	2.8	0.0	0.0	0.0	0.0	0.0
FN	0.0	0.0	0.0	0.0	0.0	0.0	0.0	0.0	0.0	93.4	93.0	91.8
FP	6.8	6.0	5.8	0.6	0.6	0.4	20.6	0.0	0.0	1.0	0.0	0.0
EXACT	93.2	94.0	94.2	99.4	99.4	99.6	79.4	100	100	6.2	7.0	8.2

Data were generated with *n* = 500, *J* = 6 for all *i*, an exchangeable correlation structure with *α* = 0.8, error variance $\sigma_{\epsilon }^{2}=1$ and autocorrelation coefficient *ρ* = 0.25 inducing correlation among the effect modifiers and noise covariates. Results are obtained from 500 independent simulations. FN: % of false negatives, FP: % of false positives, EXACT: % of exact selections, AFP: average false positives, EM: effect modifier, Indep: independent, Exch: exchangeable, UN: unstructured, Scenario 1: treatment model correct, Scenario 2: treatment-free model correct, Scenario 3: both correct, Scenario 4: both incorrect.

**TABLE 5:** Estimated blip parameters and corresponding standard errors under different working correlation structures for the hemodiafiltration study.

	Indep		Exch		AR1		UN	
	Est	SE	Est	SE	Est	SE	Est	SE
CHUM	−0.83	0.24	−1.51	0.32	−1.85	0.31	−1.62	0.32
CHUM ×cancer	–	–	–	–	3.89	0.78	–	–

## Data Availability

The data that support the findings in this paper are available on reasonable request to Michèle Bally (email: michele.bally.chum@ssss.gouv.qc.ca) conditional on approval from the ethics committee of the Centre Hospitalier de l’Université de Montréal.
