# Supplementary material for: Penalized G-estimation for effect modifier selection in a structural nested mean model for repeated outcomes
Source: Biometrics. Author manuscript; Available in PMC 2026 Jun 16. (PMC13271025; doi:10.1093/biomtc/ujae165)
Supplement: Supplementary Material [file NIHMS2179184-supplement-Supplementary_Material.pdf]

Supplementary Materials for "Penalized G-estimation for effect modifier selection in a structural nested mean model for repeated outcomes" by Ajmery Jaman, Guanbo Wang, Ashkan Ertefaie, Michèle Bally, Renée Lévesque, Robert W. Platt, and Mireille E. Schnitzer

In this document, we provide the computational form of the efficient score, the method of moment estimators for the variance and the correlation parameters, the details of tuning parameter selection, the required regularity conditions for the asymptotic properties to hold, proofs of the Theorems 1 and 2 of the main manuscript, computational details of the sandwich variance estimate, the additional simulation results, and some descriptive statistics for the data.

S1 Form of efficient score

The efficient score vector is $\mathbf{S}^{\text{eff}}(\boldsymbol{\theta}) = \{\mathbf{S}^{\text{eff}}(\boldsymbol{\delta})^\top, \mathbf{S}^{\text{eff}}(\boldsymbol{\psi})^\top\}^\top$, where

$$\begin{aligned}
\mathbf{S}^{\text{eff}}(\boldsymbol{\delta}) &= \sum_{i=1}^{n} \Big\{\frac{\partial \boldsymbol{\mu}(\mathbf{H}_i;\boldsymbol{\delta})}{\partial \boldsymbol{\delta}^\top}\Big\}^\top Var(\mathbf{U}_i|\mathbf{H}_i)^{-1}\{\mathbf{U}_i - E(\mathbf{U}_i|\mathbf{H}_i)\} \\
&= \sum_{i=1}^{n} \mathbf{H}_i^\top Var(\mathbf{U}_i|\mathbf{H}_i)^{-1}\{\mathbf{U}_i - E(\mathbf{U}_i|\mathbf{H}_i)\} \\
\mathbf{S}^{\text{eff}}(\boldsymbol{\psi}) &= \sum_{i=1}^{n} \Big[\frac{\partial \boldsymbol{\gamma}^*(\mathbf{A}_i,\mathbf{H}_i;\boldsymbol{\psi})}{\partial \boldsymbol{\psi}^\top} - E\Big\{\frac{\partial \boldsymbol{\gamma}^*(\mathbf{A}_i,\mathbf{H}_i;\boldsymbol{\psi})}{\partial \boldsymbol{\psi}^\top}|\mathbf{H}_i\Big\}\Big]^\top Var(\mathbf{U}_i|\mathbf{H}_i)^{-1}\{\mathbf{U}_i - E(\mathbf{U}_i|\mathbf{H}_i)\} \\
&= \sum_{i=1}^{n} \Big[\{\mathbf{A}_i - E(\mathbf{A}_i|\mathbf{H}_i)\}\mathbf{H}_i\Big]^\top Var(\mathbf{U}_i|\mathbf{H}_i)^{-1}\{\mathbf{U}_i - E(\mathbf{U}_i|\mathbf{H}_i)\}
\end{aligned}$$

S2 Method of moments estimators for σ^2 and α

Calculate the variance parameter as

$$\hat{\sigma}^2 = \frac{1}{n}\sum_{i=1}^{n}\frac{1}{J}\sum_{j=1}^{J} e_{ij}^2$$

and the correlation parameter(s) as shown in Table S1.

Table S1: Common choices for working correlation structures and corresponding estimators of the correlation parameter.

Working structure	$\widehat{\text{Corr}}(U_{ij}, U_{ik})$	Estimator
Independence	0	-
Exhangeable	$\hat{\alpha}$	$\hat{\alpha} = \frac{1}{n\hat{\sigma}^2}\sum_{i=1}^{n}\frac{1}{J(J-1)}\sum\sum_{j\neq k} e_{ij}e_{ik}$
AR(1)	$\hat{\alpha}^{\lvert j-k\rvert}$	$\hat{\alpha} = \frac{1}{n\hat{\sigma}^2}\sum_{i=1}^{n}\frac{1}{J-1}\sum_{j\leq J-1} e_{ij}e_{i,(j+1)}$
Unstructured	$\hat{\alpha}_{jk}$	$\hat{\alpha}_{jk} = \frac{1}{n\hat{\sigma}^2}\sum_{i=1}^{n} e_{ij}e_{ik}$

S3 Tuning parameter selection

The oracle properties of the penalized estimators rely on the proper choice of tuning parameter. Johnson et al. (2008) considered the generalized cross-vaidation (GCV) statistic (Wahba, 1985) for selecting the tuning parameter in penalized estimating equations. The use of GCV for such selection was suggested by Tibshirani (1996) and Fan and Li (2001). However, Wang et al. (2007) showed that tuning parameter selection using GCV causes a nonignorable overfitting effect even if the sample size goes to infinity. Considering the number of covariates K as fixed, Wang et al. (2007) proposed the Bayesian information criterion (BIC) for tuning parameter selection which showed consistency in the identification of the true model. Later, a modification

in the BIC criterion was proposed by Wang et al. (2009) in a moderately high-dimensional setup ($K < n$) and by Fan and Tang (2013) in an ultra-high dimensional setup ($K >> n$). All of these criteria have the following general form

$$\text{measure of model fit} + \tau_n \times \text{measure of model complexity}, \tag{S1}$$

where τ_n is a positive sequence.

In the context of variable selection for individualized treatment rules, Bian et al. (2023) and Moodie et al. (2023) used doubly robust information criteria for tuning parameter selection. These criteria are similar to the criterion of Fan and Tang (2013), except that Bian et al. (2023) and Moodie et al. (2023) introduced a weighted measure of model fitting that incorporates overlap weights. To construct the measure of model fitting we follow an approach similar to Wang et al. (2007) and construct the weighted loss function following Moodie et al. (2023). We define the weighted loss function corresponding to the G-estimating equations as

$$L(\tilde{\boldsymbol{\theta}}_{\lambda_n}) = \sum_{i=1}^{n}\sum_{j=1}^{J} |\mathbf{A}_{ij} - E(\mathbf{A}_{ij}|\mathbf{H}_{ij})| \times (\mathbf{Y}_{ij} - \mathbf{H}_{ij}^{\top}\tilde{\boldsymbol{\delta}}_{\lambda_n} - \mathbf{A}_{ij}\mathbf{H}_{ij}^{\top}\tilde{\boldsymbol{\psi}}_{\lambda_n})^2, \tag{S2}$$

where $|\mathbf{A}_{ij} - E(\mathbf{A}_{ij}|\mathbf{H}_{ij})|$ are the overlap weights and $\tilde{\boldsymbol{\theta}}_{\lambda_n}$ denotes the penalized estimate obtained under a fixed value of the tuning parameter λ_n. Since the joint distribution of the data under a non-diagonal correlation structure is unknown/undefined, we construct the loss function associated with the estimating equations under an independence assumption. For the penalized estimating equations, the measure of model complexity can be defined with the following generalized degrees of freedom (Johnson et al., 2008)

$$\text{DF}_{\lambda_n} = trace\left[\left\{\hat{\mathbf{H}}_n(\tilde{\boldsymbol{\theta}}_{\lambda_n}) + n\mathbf{E}_n(\tilde{\boldsymbol{\theta}}_{\lambda_n})\right\}^{-1}\hat{\mathbf{H}}_n(\tilde{\boldsymbol{\theta}}_{\lambda_n})\right].$$

In our setup, we propose the following doubly robust information criterion:

$$\text{DRIC}_{\lambda_n} = \log\left\{\frac{L(\tilde{\boldsymbol{\theta}}_{\lambda_n})}{n \times J}\right\} + \tau_n \times \frac{\text{DF}_{\lambda_n}}{n}. \tag{S3}$$

We set $\tau_n = \log\{\log(n)\}\log(2K)$, a consideration similar to Fan and Tang (2013), where $2K$ represents the dimension of $\boldsymbol{\theta}$. Note that we can even consider $\tau_n = \log(n)$ in the low dimensional setting.

We compute the DRIC for a sequence of values for λ_n which are in decreasing order. Typically, the first element ($\lambda_{\max}$) in this sequence is the lowest positive number such that all the effect modifiers are eliminated by the method. The last element ($\lambda_{\min}$) is a value close to zero for which none of the effect modifiers are eliminated. And we consider around one hundred values in between so that we have a fine grid. The optimal tuning parameter is the value of λ_n that corresponds to the minimum value of DRIC_{λ_n}.

Note that Wang et al. (2012) used cross-validation for selecting the tuning parameter in a penalized

generalized estimating equation, minimizing the prediction error under an independence assumption. We followed a similar idea and constructed the loss function in the DRIC using an independent correlation structure. Although we considered a repeated outcomes setup, in our study, we did not consider the quasi-information criterion (QIC) (Pan, 2001), which extends the AIC in the longitudinal setup. Because the QIC might experience similar overfitting issues as the AIC and since our goal is to select the true effect modifiers in SNMMs, we instead used the DRIC for tuning parameter selection. Structurally, DRIC has resemblance to the BIC.

S4 Regularity conditions

For establishing the asymptotic theory of the penalized G-estimator, we need the following regularity conditions (Wang et al., 2012) to hold, some of which maybe further relaxed. Majority of these conditions are analogous to the regularity conditions for the generalized estimating equations for longitudinal data.

(C1) All variables in $\mathbf{D}_{ij}$, $i = 1, \ldots, n$, $j = 1, \ldots, J$, are uniformly bounded.

(C2) The unknown parameter $\boldsymbol{\theta}_n$ belongs to a compact subset $\boldsymbol{\Theta} \subseteq R^{2K}$ and the true parameter $\boldsymbol{\theta}_0$ lies in the interior of $\boldsymbol{\Theta}$.

(C3) There exists finite positive constants c_1 and c_2 such that

$$c_1 \leq \omega_{min}\left(\frac{\sum_{i=1}^{n} \mathbf{D}_i^\top (\mathbf{H}_i \;\; \mathbf{A}_i \cdot \mathbf{H}_i)}{n}\right) \leq \omega_{max}\left(\frac{\sum_{i=1}^{n} \mathbf{D}_i^\top (\mathbf{H}_i \;\; \mathbf{A}_i \cdot \mathbf{H}_i)}{n}\right) \leq c_2,$$

where $\omega_{min}(\mathbf{D})$ and $\omega_{max}(\mathbf{D})$ denote the minimum and maximum of the eigenvalues, respectively, of the matrix $\mathbf{D}$.

(C4) The common true correlation matrix $\mathbf{R}_0$ for the observed outcomes has eigen values bounded away from zero and $+\infty$. The estimated working correlation matrix $\hat{\mathbf{R}}$ satisfies $||\hat{\mathbf{R}}^{-1} - \overline{\mathbf{R}}^{-1}|| = O_p(\sqrt{1/n})$, where $\overline{\mathbf{R}}$ is a constant positive definite matrix with eigen values bounded away from zero and $+\infty$. Note that $||\mathbf{D}|| = \{trace(\mathbf{D}\mathbf{D}^\top)\}^{1/2}$ denotes the Frobenius norm of the matrix $\mathbf{D}$.

(C5) Let $\boldsymbol{\xi}_i(\boldsymbol{\theta}_n) = (\boldsymbol{\xi}_{i1}(\boldsymbol{\theta}_n), \ldots, \boldsymbol{\xi}_{in_i}(\boldsymbol{\theta}_n))^\top = \mathbf{Q}_i^{-1/2}(\mathbf{Y}_i - \mathbf{g}_i(\boldsymbol{\theta}_n))$. There exists a finite constant $d_1 > 0$ such that $E(||\boldsymbol{\xi}_i(\boldsymbol{\theta}_0)||^{2+\kappa}) \leq d_1$ for all i and some $\kappa > 0$; and there esists positive constants d_2 and d_3 such that $E(\exp(d_2|\xi_{ij}(\boldsymbol{\theta}_0))|\mathbf{D}_i) \leq d_3$, uniformly in $i = 1, \ldots, n$, $j = 1, \ldots, J$.

(C6) Let $T_n = \{\boldsymbol{\theta}_n : ||\boldsymbol{\theta}_n - \boldsymbol{\theta}_0|| \leq \Delta\sqrt{1/n}\}$, then $g'(\mathbf{D}_{ij}\boldsymbol{\theta}_n)$, $i = 1, \ldots, n$, $j = 1, \ldots, J$, are uniformly bounded away from 0 and ∞ on T_n; $g''(\mathbf{D}_{ij}\boldsymbol{\theta}_n)$ and $g'''(\mathbf{D}_{ij}\boldsymbol{\theta}_n)$, $i = 1, \ldots, n$, $j = 1, \ldots, J$, are uniformly bounded by a finite positive constant d_2 on T_n; $g'(.)$, $g''(.)$ and $g'''(.)$ denote the first, second and third derivatives of the function $g(.)$, respectively.

(C7) When s is not fixed, assuming $\min_{m\in B}|\theta_{0m}|/\lambda_n \to \infty$ as $n \to \infty$ and $s_n^3 n^{-1} = o(1)$, $\lambda_n \to 0$, $s_n^2(\log n)^4 = o(n\lambda_n^2)$, $\log(K_n) = o(n\lambda_n^2/(\log n)^2)$, $K_n s_n^4(\log n)^6 = o(n^2\lambda_n^2)$, and $K_n s_n^3(\log n)^8 = o(n^2\lambda_n^4)$. Note that λ_n is the tuning parameter.

S5 Proof of Theorem 1

We consider the sets $F_m = \{\tilde{\theta}_m \neq 0\}$ such that $m \in B^c$. To prove this theorem it is sufficient to show that for any $\epsilon > 0$, $P(F_m) < \epsilon$ as $n \to \infty$. Since $\tilde{\theta}_m = O_p(n^{-1/2})$, there exists some Z such that

$$P(F_m) < \epsilon/2 + P(\tilde{\theta}_m \neq 0, |\tilde{\theta}_m| < Zn^{-1/2}) \text{ as } n \to \infty. \tag{S4}$$

If $\tilde{\boldsymbol{\theta}}$ is an approximate zero crossing, on the set $\{\tilde{\theta}_m \neq 0, |\tilde{\theta}_m| < Zn^{-1/2}\}$ we have,

$$\{n^{-1/2}S_m^{\text{eff}}(\theta_0) + n^{1/2}\mathbf{H}_{(m)}(\boldsymbol{\theta}_0)(\tilde{\theta} - \theta_0) + o_p(1) - n^{1/2}q_{\lambda_n}(|\tilde{\theta}_m|)\text{sign}(\tilde{\theta}_m)\}^2 = o_p(1),$$

where $\mathbf{H}_{(m)}(\boldsymbol{\theta}_0)$ is the m-th row of $\mathbf{H}(\boldsymbol{\theta}_0)$. In the above equation, the first three terms are of order $O_p(1)$. Hence, there exists some Z' such that

$$P(\tilde{\theta}_m \neq 0, |\tilde{\theta}_m| < Zn^{-1/2}, n^{1/2}q_{\lambda_n}(|\tilde{\theta}_m|) > Z') < \epsilon/2 \text{ as } n \to \infty. \tag{S5}$$

If the condition (C.b.2) holds, $\tilde{\theta}_m \neq 0$ and $|\tilde{\theta}_m| < Zn^{-1/2}$ together imply that $n^{1/2}q_{\lambda_n}(|\tilde{\theta}_m|) > Z'$ as $n \to \infty$. Therefore, $P(\tilde{\theta}_m \neq 0, |\tilde{\theta}_m| < Zn^{-1/2}) = P(\tilde{\theta}_m \neq 0, |\tilde{\theta}_m| < Zn^{-1/2}, n^{1/2}q_{\lambda_n}(|\tilde{\theta}_m|) > Z')$ and from equations (S4) and (S5) we obtain that

$$P(F_m) < \epsilon/2 + P(\tilde{\theta}_m \neq 0, |\tilde{\theta}_m| < Zn^{-1/2}, n^{1/2}q_{\lambda_n}(|\tilde{\theta}_m|) > Z') < \epsilon. \tag{S6}$$

S6 Proof of Theorem 2

Let $\mathbf{S}_B^{\text{eff}}(\boldsymbol{\theta})$ represents the vector of elements in $\mathbf{S}^{\text{eff}}(\boldsymbol{\theta})$ corresponding to θ_m's such that $m \in B$ and $\mathbf{H}_B(\boldsymbol{\theta})$ represents the $s \times s$ submatrice of $\mathbf{H}(\boldsymbol{\theta})$. Under conditions (C.a) and (C.b.1)

$$n^{-1/2}\mathbf{S}_B^{\text{eff}}(\boldsymbol{\theta}_0) + n^{1/2}\mathbf{H}_B(\boldsymbol{\theta}_0)(\tilde{\boldsymbol{\theta}}_n^B - \boldsymbol{\theta}_0^B) - n^{1/2}\mathbf{q}_{\lambda_n}(|\tilde{\boldsymbol{\theta}}_n^B|)\text{sign}(\tilde{\boldsymbol{\theta}}_n^B) = \mathbf{o}_p(1). \tag{S7}$$

If we apply the Taylor series approximation of $\mathbf{q}_{\lambda_n}(|\tilde{\boldsymbol{\theta}}_n^B|)\text{sign}(\tilde{\boldsymbol{\theta}}_n^B)$ around $\boldsymbol{\theta}_0^B$ in equation (S7), we obtain

$$
\begin{aligned}
\mathbf{o}_p(1) &\approx n^{-1/2}\mathbf{S}_B^{\text{eff}}(\boldsymbol{\theta}_0) + n^{1/2}\mathbf{H}_B(\boldsymbol{\theta}_0)(\tilde{\boldsymbol{\theta}}_n^B - \boldsymbol{\theta}_0^B) - n^{1/2}\Big\{\mathbf{q}_{\lambda_n}(|\boldsymbol{\theta}_0^B|)\text{sign}(\boldsymbol{\theta}_0^B) + \mathbf{q}'_{\lambda_n}(|\boldsymbol{\theta}_0^B|)\text{sign}(\boldsymbol{\theta}_0^B)(\tilde{\boldsymbol{\theta}}_n^B - \boldsymbol{\theta}_0^B)\Big\} \\
&= n^{-1/2}\mathbf{S}_B^{\text{eff}}(\boldsymbol{\theta}_0) + n^{1/2}\Big\{\mathbf{H}_B(\boldsymbol{\theta}_0) + \mathbf{W}_n(\boldsymbol{\theta}_0^B)\Big\}(\tilde{\boldsymbol{\theta}}_n^B - \boldsymbol{\theta}_0^B) - n^{1/2}\mathbf{q}_{\lambda_n}(|\boldsymbol{\theta}_0^B|)\text{sign}(\boldsymbol{\theta}_0^B) \\
&= n^{-1/2}\mathbf{S}_B^{\text{eff}}(\boldsymbol{\theta}_0) + n^{1/2}\Big\{\mathbf{H}_B(\boldsymbol{\theta}_0) + \mathbf{W}_n(\boldsymbol{\theta}_0^B)\Big\}\Big[\tilde{\boldsymbol{\theta}}_n^B - \boldsymbol{\theta}_0^B - \Big\{\mathbf{H}_B(\boldsymbol{\theta}_0) + \mathbf{W}_n(\boldsymbol{\theta}_0^B)\Big\}^{-1}\mathbf{q}_{\lambda_n}(|\boldsymbol{\theta}_0^B|)\text{sign}(\boldsymbol{\theta}_0^B)\Big] \\
&= n^{-1/2}\mathbf{S}_B^{\text{eff}}(\boldsymbol{\theta}_0) + n^{1/2}\Big\{\mathbf{H}_B(\boldsymbol{\theta}_0) + \mathbf{W}_n(\boldsymbol{\theta}_0^B)\Big\}\Big[\tilde{\boldsymbol{\theta}}_n^B - \boldsymbol{\theta}_0^B + \Big\{\mathbf{H}_B(\boldsymbol{\theta}_0) + \mathbf{W}_n(\boldsymbol{\theta}_0^B)\Big\}^{-1}\mathbf{b}_n\Big].
\end{aligned}
$$

Since by the condition (C.a) $n^{-1/2}\mathbf{S}_B^{\text{eff}}(\boldsymbol{\theta}_0) \xrightarrow{d} N(\mathbf{0}, \mathbf{I}_B(\boldsymbol{\theta}_0))$, we have

$$
\begin{aligned}
& n^{1/2}\Big\{\mathbf{H}_B(\boldsymbol{\theta}_0) + \mathbf{W}_n(\boldsymbol{\theta}_0^B)\Big\}\Big[\tilde{\boldsymbol{\theta}}_n^B - \boldsymbol{\theta}_0^B + \Big\{\mathbf{H}_B(\boldsymbol{\theta}_0) + \mathbf{W}_n(\boldsymbol{\theta}_0^B)\Big\}^{-1}\mathbf{b}_n\Big] \\
&\approx -n^{-1/2}\mathbf{S}_B^{\text{eff}}(\boldsymbol{\theta}_0) + \mathbf{o}_p(1) \xrightarrow{d} N(\mathbf{0}, \mathbf{I}_B(\boldsymbol{\theta}_0)).
\end{aligned}
$$

S7 Computation of the sandwich variance estimate

For binary treatment, we assume the following logistic regression model for the pooled data

$$
\log\left\{\frac{P(A_{ij}=1|\mathbf{H}_{ij})}{1-P(A_{ij}=1|\mathbf{H}_{ij})}\right\} = \mathbf{H}_{ij}\boldsymbol{\beta},
$$

where $\boldsymbol{\beta}$ is the vector of treatment model parameters. Usually, $\boldsymbol{\beta}$ is unknown and needs to be estimated using the data. Hence, for appropriate estimation of the asymptotic variance of $\tilde{\boldsymbol{\psi}}_n$, we need to account for the uncertainty associated with the estimation of $\boldsymbol{\beta}$. The joint score of $\hat{\boldsymbol{\beta}}$ and $\tilde{\boldsymbol{\psi}}_n$ is

$$
S_i = \begin{bmatrix} S_{\boldsymbol{\beta},(i)} \\ S_{\boldsymbol{\psi},(i)} \end{bmatrix} = \begin{bmatrix} \mathbf{H}_i^\top\{\mathbf{A}_i - E(\mathbf{A}_i|\mathbf{H}_i)\} \\ [\{\mathbf{A}_i - E(\mathbf{A}_i|\mathbf{H}_i)\}\cdot\mathbf{H}_i]^\top\,\mathbf{V}_i^{-1}\,(\mathbf{Y}_i - \mathbf{A}_i\cdot\mathbf{H}_i\boldsymbol{\psi} - \mathbf{H}_i\boldsymbol{\delta}) \end{bmatrix}.
$$

Then we calculate

$$
\hat{I}_n(\hat{\boldsymbol{\beta}}, \tilde{\boldsymbol{\psi}}) = \sum_{i=1}^n S_i\, S_i^\top\,|_{\boldsymbol{\beta}=\hat{\boldsymbol{\beta}}, \boldsymbol{\psi}=\tilde{\boldsymbol{\psi}}} = \begin{bmatrix} \hat{I}_{\boldsymbol{\beta\beta}} & \hat{I}_{\boldsymbol{\beta\psi}} \\ \hat{I}_{\boldsymbol{\psi\beta}} & \hat{I}_{\boldsymbol{\psi\psi}} \end{bmatrix}.
$$

By Taylor's expansion and Slutskey's theorem, $I_n(\tilde{\boldsymbol{\psi}})$ can be consistently estimated by

$$
\hat{I}_n(\tilde{\boldsymbol{\psi}}) = \hat{I}_{\boldsymbol{\psi\psi}} - \hat{I}_{\boldsymbol{\psi\beta}}\hat{I}_{\boldsymbol{\beta\beta}}^{-1}\hat{I}_{\boldsymbol{\beta\psi}}.
$$

The estimate of $H_n(\tilde{\boldsymbol{\psi}})$ is

$$\begin{aligned}\hat{H}_n(\tilde{\boldsymbol{\psi}}) &= -\sum_{i=1}^{n} \frac{\partial S_{\boldsymbol{\psi},(i)}}{\partial \boldsymbol{\psi}^\top} \Big|_{\boldsymbol{\beta}=\hat{\boldsymbol{\beta}},\boldsymbol{\psi}=\tilde{\boldsymbol{\psi}}} \\ &= \sum_{i=1}^{n} [\{\mathbf{a}_i - E(\mathbf{A}_i|\mathbf{H}_i)\} \cdot \mathbf{h}_i]^\top \hat{\mathbf{V}}_i^{-1} [\{\mathbf{a}_i - E(\mathbf{A}_i|\mathbf{H}_i)\} \cdot \mathbf{h}_i] \Big|_{\boldsymbol{\beta}=\hat{\boldsymbol{\beta}},\boldsymbol{\psi}=\tilde{\boldsymbol{\psi}}}.\end{aligned}$$

Note that $\mathbf{S}_{\boldsymbol{\psi},(i)} = \mathbf{S}^{\text{eff}}_{\boldsymbol{\psi},(i)}$.

S8 Additional simulation results

S8.1 Setup with unequal number of observations from patients

We chose the number of observations of each subject from the set {4, 5 and 6} with an equal selection probability 1/3. The performance of our estimator did not change (compare Table S2 to the bottom row of Table 2 of the main paper).

Table S2: Model selection performance of the penalized G-estimator for data generated with $n = 500$, unequal clusters having size 4, 5 or 6, an exchangeable correlation structure among the repeated outcomes with correlation parameter $\alpha = 0.8$, an autocorrelation coefficient $\rho = 0.25$ deciding the correlation among the EM's and noise covariates, and error variance $\sigma_\epsilon^2 = 1$. Results are obtained with a misspecified treatment-free model from 500 independent simulations for Setting 1 and Setting 2.

Working	**Setting 1 (Stronger EM)**				**Setting 2 (Weaker EM)**			
correlation	FN	FP	EXACT	AFP	FN	FP	EXACT	AFP
Indep	0.2	6.0	93.8	6.00	0.6	5.6	94.0	6.00
**Exch**	0.4	6.0	**93.6**	6.00	0.8	4.8	**94.6**	4.80
UN	1.4	5.0	93.6	5.00	1.4	5.2	93.6	5.40

FN: % of false negatives, FP: % of false positives, EXACT: % of exact selections, AFP: average false positives, EM: effect modifier, Indep: independent, Exch: exchangeable, UN: unstructured

S8.2 Setup where past outcome affects future exposure and future outcome

We performed additional simulations considering a situation when past outcome is allowed to affect future treatment decision, i.e., we have effects like $Y_{j-1} \to A_j$. We generated the binary exposure according to the probability

$$P(A_j = 1|\mathbf{H}_j) = \frac{\exp\left(\beta_0 + \beta_1 l^{(1)} + \beta_2 l^{(2)} + \sum_{m=3}^{6} \beta_m l_j^{(m)} + \beta_7 a_{j-1} + \beta_8 y_{j-1}\right)}{1 + \exp\left(\beta_0 + \beta_1 l^{(1)} + \beta_2 l^{(2)} + \sum_{m=3}^{6} \beta_m l_j^{(m)} + \beta_7 a_{j-1} + \beta_8 y_{j-1}\right)}, \tag{S8}$$

where the coefficients $\beta_0, \ldots, \beta_7$ were the same as considered in other simulations presented in the main manuscript and we set $\beta_8 = -0.8$. The results regarding the model-selection consistency are shown in Table S3.

Table S3: Model selection performance of the proposed penalized G-estimator under a data generating mechanism when we additionally have $Y_{j-1} \to A_j$. Data were generated with $n = 200$ vs. 500, $n_i = 6$ for all i, an exchangeable correlation structure among the repeated outcomes with $\alpha = 0.8$, an autocorrelation coeffcient $\rho = 0.25$ deciding the correlation among the EM's and noise covariates, and error variance $\sigma_\epsilon^2 = 1$. Results are obtained with a misspecified treatment-free model from 500 independent simulations for Setting 1 and Setting 2.

	Working	**Setting 1 (Stronger EM)**				**Setting 2 (Weaker EM)**			
	correlation	FN	FP	EXACT	AFP	FN	FP	EXACT	AFP
	Indep	11.8	5.4	83.0	0.06	35.8	5.6	61.4	0.07
$n = 200$	**Exch**	12.2	5.2	**82.8**	0.06	36.6	4.4	**61.2**	0.05
	UN	13.2	5.0	81.8	0.05	35.6	3.6	62.0	0.04
	Indep	1.4	4.0	94.6	0.04	1.6	2.6	96.0	0.03
$n = 500$	**Exch**	1.4	3.6	**95.0**	0.04	1.8	2.2	**96.2**	0.02
	UN	1.8	2.8	95.4	0.03	1.8	2.0	96.2	0.02

FN: % of false negatives, FP: % of false positives, EXACT: % of exact selections,
AFP: average false positives, EM: effect modifier,
Indep: independent, Exch: exchangeable, UN: unstructured

We performed another simulation study where the treatment generating mechanism was the same as shown in equation (S8), and additionally, we allowed past outcome to affect future outcome. In this case, the treatment-free model we considered is

$$\mu_j(\mathbf{h}_j; \boldsymbol{\delta}) = \delta_0 + \delta_1 l^{(1)} + \delta_2 l^{(2)} + \sum_{m=3}^{6} \delta_m l_j^{(m)} + \delta_7 \exp(l_j^{(5)}) + \delta_8 a_{j-1} + \delta_9 y_{j-1}, \tag{S9}$$

where the coefficients $\delta_0, \ldots, \delta_8$ were the same as considered in other simulations presented in the main manuscript and we set $\delta_9 = 0.7$. The results regarding the model-selection consistency are shown in Table S4. Under both setups (see Tables S3 and S4), the selection rates of the true effect modifiers were good when we considered a larger sample size, specifically for the setting of weaker effect modification.

Table S4: Model selection performance of the proposed penalized G-estimator under a data generating mechanism when we additionally have $Y_{j-1} \to A_j$ and $Y_{j-1} \to Y_j$. Data were generated with $n = 200$ vs. 500, $n_i = 6$ for all i, an exchangeable correlation structure among the repeated outcomes with $\alpha = 0.8$, an autocorrelation coeffcient $\rho = 0.25$ deciding the correlation among the EM's and noise covariates, and error variance $\sigma_\epsilon^2 = 1$. Results are obtained with a misspecified treatment-free model from 500 independent simulations for Setting 1 and Setting 2.

	Working	**Setting 1 (Stronger EM)**				**Setting 2 (Weaker EM)**			
	correlation	FN	FP	EXACT	AFP	FN	FP	EXACT	AFP
	Indep	14.2	23.2	65.8	0.35	46.2	25.2	41.2	0.34
$n = 200$	**Exch**	13.4	24.4	**65.0**	0.35	42.6	23.8	**45.6**	0.33
	UN	13.8	22.0	66.6	0.31	43.0	24.4	44.6	0.34
	Indep	2.0	16.4	81.6	0.20	7.8	15.2	78.0	0.20
$n = 500$	**Exch**	2.0	17.4	**80.6**	0.22	7.4	16.2	**77.4**	0.21
	UN	0.8	17.0	82.2	0.21	6.4	16.2	78.8	0.20

FN: % of false negatives, FP: % of false positives, EXACT: % of exact selections,
AFP: average false positives, EM: effect modifier,
Indep: independent, Exch: exchangeable, UN: unstructured

S8.3 Simulations in high-dimensional setting

To generate the data for j-th session ($j = 1, \dots, J$) of each subject, we generated one baseline confounder as $L^{(1)} \sim N(0,1)$, and the time varying confounders and noise covariates as $L_j^{(2)}, \dots, L_j^{(5)}, X_j^{(1)}, \dots, X_j^{(K-5)} \sim MVN_{K-1}\Big((\boldsymbol{\mu}_{L,j}^\top, \boldsymbol{\mu}_{X,j}^\top)^\top, \mathbf{V}\Big)$, where $\mu_{L,j}^{(k)} = 0.3\, l_{j-1}^{(k)} + 0.3\, a_{j-1}$ for $k = 2, 3, 4$ and 5, and $\mu_{X,j}^{(r)} = 0.5\, x_{j-1}^{(r)}$ for $r = 1, \dots, K-5$. The covariance matrix $\mathbf{V}$ has (r,s)-th element equal to $\rho^{|r-s|}$ for $r, s = 1, \dots, K-1$. We generated the binary exposure according to the probability

$$P(A_j = 1|\mathbf{H}_j) = \frac{\exp\left(\beta_0 + \beta_1 l^{(1)} + \sum_{m=2}^{5} \beta_m l_j^{(m)} + \beta_6 a_{j-1}\right)}{1 + \exp\left(\beta_0 + \beta_1 l^{(1)} + \sum_{m=2}^{5} \beta_m l_j^{(m)} + \beta_6 a_{j-1}\right)}. \tag{S10}$$

The generation of $\boldsymbol{\epsilon}$ is same as before. We constructed the outcome as $y_j = \mu_j(\mathbf{h}_j; \boldsymbol{\delta}) + \gamma_j^*(a_j, \mathbf{h}_j; \boldsymbol{\psi}) + \epsilon_j$, where

$$\mu_j(\mathbf{h}_j; \boldsymbol{\delta}) = \delta_0 + \delta_1 l^{(1)} + \sum_{m=2}^{5} \delta_m l_j^{(m)} + \delta_6 a_{j-1} + \sum_{m=1}^{20} \delta_{6+m}\, x_j^{(m)} + \sum_{m=21}^{K-5} \delta_{6+m}\, x_j^{(m)} \\ + \delta_{K+2} l^{(1)} l_j^{(4)} + \delta_{K+3} l_j^{(2)} l_j^{(3)} + \delta_{K+4} \sin(l_j^{(3)} - l_j^{(4)}) + \delta_{K+5} \cos(2 l_j^{(5)})$$

and $\gamma_j^*(a_j, \mathbf{h}_j; \boldsymbol{\psi}) = (\psi_0 + \psi_1 l^{(1)} + \sum_{m=2}^{5} \psi_m l_j^{(m)} + \psi_6 a_{j-1} + \sum_{m=1}^{20} \psi_{6+m}\, x_j^{(m)} + \sum_{m=21}^{K-5} \psi_{6+m}\, x_j^{(m)}) a_j$. Let $\boldsymbol{\beta} = (\beta_0, \dots, \beta_6)^\top$, $\boldsymbol{\delta} = (\delta_0, \dots, \delta_{K+5})^\top$ and $\boldsymbol{\psi} = (\psi_0, \dots, \psi_{K+1})^\top$. We set

$$\begin{aligned} \boldsymbol{\beta} &= (0, 1, 1, 1, 1, 1, -0.8)^\top \\ \boldsymbol{\delta} &= (1, 1, 1.2, -1, 1, -1.2, 1, 1, \dots, 1, 0, \dots, 0, -0.8, 1, 1.2, -2)^\top \\ \boldsymbol{\psi} &= (1, 1.5, 1.2, -1.3, 0, 1, 2, 0, \dots, 0, 0, \dots, 0)^\top \end{aligned}$$

Note that $X^{(1)}$ to $X^{(20)}$ have impact on the outcome only and the coefficients of $X^{(21)}$ to $X^{(K-5)}$ were set to zero in $\mu_j(\mathbf{h}_j; \boldsymbol{\delta})$. Though we set the coefficients of all the X's to zero in $\gamma_j^*(a_j, \mathbf{h}_j; \boldsymbol{\psi})$, it is possible to investigate effect heterogeneity by the X variables also. We set $K = 50$ vs. 100, $n = 200$ vs. 500, $\rho = 0.3$, $\sigma_\epsilon^2 = 1$, and $\alpha = 0.8$.

When we performed penalized estimation the outcome model was misspecified, because

- $L^{(1)} \times L^{(4)}$ and $L^{(2)} \times L^{(3)}$ interactions were ignored
- $\sin(L^{(3)} - L^{(4)})$ and $\cos(2L^{(5)})$ terms were ignored
- Covariate $X^{(10)}$ which also affects the outcome was considered unmeasured

The selection performance of the estimator was poor in small samples and increasing the number of variables from 50 to 100 was associated with an increase in the false negative rates (see Table S5). However, the selection performance was good when we increased the sample size from 200 to 500.

Table S5: Model selection performance of the proposed penalized G-estimator in high dimension. Data were generated with $n = 200$ vs. 500, $n_i = 6$ for all i, an exchangeable correlation structure among the repeated outcomes with $\alpha = 0.8$, an autocorrelation coeffcient $\rho = 0.3$ deciding the correlation among the EM's and noise covariates, and error variance $\sigma_\epsilon^2 = 1$. Results are obtained with a misspecified treatment-free model from 500 independent simulations for two different values of dimension K.

	Working correlation	FN	FP	EXACT	AFP	FN	FP	EXACT	AFP
		**n = 200**				**n = 500**			
	Indep	34.0	1.2	64.8	1.20	2.4	2.0	95.6	2.00
**K = 50**	**Exch**	35.4	1.2	**63.4**	1.20	2.6	1.6	**95.8**	1.60
	UN	35.0	1.4	63.6	1.40	2.6	1.8	95.6	1.80
	Indep	44.4	0.0	55.6	0.00	6.2	0.4	93.4	0.40
**K = 100**	**Exch**	48.2	0.2	**51.6**	0.20	6.4	0.0	**93.6**	0.00
	UN	47.2	0.2	52.6	0.20	6.6	0.0	93.4	0.00

FN: % of false negatives, FP: % of false positives, EXACT: % of exact selections,
AFP: average false positives, EM: effect modifier,
Indep: independent, Exch: exchangeable, UN: unstructured

S8.4 Comparison with the method in Boruvka et al. (2018)

Table S6: Performance comparison between our penalized estimates (penalized-G) and the full model estimates obtained using the method in Boruvka (2018). Estimation was performed with data generated using $n = 200$ and 500, autocorrelation coefficient $\rho = 0.25$, error variance $\sigma_\epsilon^2 = 1$, $\alpha = 0.8$ and an exchangeable correlation structure. Statistics were calculated from 500 simulations under the independent working correlation structure.

		**n = 200**						**n = 500**					
	True	**Penalized-G**			**Boruvka (2018)**			**Penalized-G**			**Boruvka (2018)**		
	Coef	Bias*	SE1	SE2	Bias	SE1	SE2	Bias	SE1	SE2	Bias	SE1	SE2
A	1	0.20	0.27	0.24	0.74	0.31	0.51	0.30	0.16	0.16	0.64	0.19	0.31
$A \times L^{(1)}$	-2.5	0.29	0.32	0.30	1.52	0.40	0.61	0.58	0.19	0.19	0.15	0.24	0.37
$A \times L^{(2)}$	1.5	0.03	0.18	0.17	0.07	0.23	0.32	0.02	0.11	0.11	0.17	0.15	0.19
$A \times L^{(3)}$	1.5	0.03	0.18	0.16	0.05	0.24	0.31	0.00	0.11	0.10	0.14	0.14	0.18
$A \times L^{(4)}$	1.5	0.03	0.18	0.17	0.16	0.24	0.32	0.13	0.11	0.11	0.06	0.14	0.19
$A \times L^{(5)}$	1.5	0.16	0.31	0.25	0.82	0.35	0.42	0.02	0.18	0.18	0.14	0.21	0.26
$A \times L^{(6)}$	0	0.22	0.07	0.03	0.17	0.24	0.35	0.22	0.05	0.02	0.46	0.14	0.21
$A \times A_{\text{Lag1}}$	2	0.01	0.38	0.31	1.21	0.41	0.68	0.09	0.19	0.20	0.76	0.23	0.41
$A \times X^{(1)}$	0	0.02	0.03	0.01	0.21	0.21	0.21	0.02	0.02	0.01	0.21	0.13	0.13
$A \times X^{(2)}$	0	0.01	0.01	0.01	0.01	0.20	0.21	0.01	0.01	0.00	0.02	0.13	0.13
$A \times X^{(3)}$	0	0.01	0.02	0.01	0.28	0.21	0.21	0.00	0.00	0.00	0.07	0.12	0.12
$A \times X^{(4)}$	0	0.00	0.00	0.00	0.09	0.20	0.21	0.00	0.00	0.00	0.05	0.12	0.12
$A \times X^{(5)}$	0	0.01	0.02	0.01	0.05	0.20	0.21	0.01	0.01	0.00	0.23	0.11	0.12
$A \times X^{(6)}$	0	0.00	0.00	0.00	0.10	0.20	0.21	0.02	0.03	0.01	0.15	0.12	0.12
$A \times X^{(7)}$	0	0.00	0.00	0.00	0.27	0.21	0.21	0.01	0.01	0.01	0.14	0.12	0.12
$A \times X^{(8)}$	0	0.01	0.03	0.01	0.08	0.21	0.21	0.00	0.00	0.00	0.05	0.12	0.12
$A \times X^{(9)}$	0	0.01	0.02	0.01	0.08	0.22	0.21	0.00	0.00	0.00	0.11	0.12	0.12
$A \times X^{(10)}$	0	0.01	0.02	0.01	0.16	0.19	0.20	0.00	0.00	0.00	0.08	0.12	0.12

*Bias (scaled bias) = $\sqrt{n} \times |\widetilde{\psi}_n - \psi_0|$, SE1: the empirical root-mean squared error (MSE), SE2: the square-root of the average of sandwich variance estimates

Our estimator has higher efficiency than Boruvka's estimator, because our method applies simultaneous elimination of spurious effect modifiers.

S9 Descriptive statistics for hemodiafiltration data

Descriptive statistics for all of the variables (continuous and binary) from Session 1 are presented in Table S7 by the two exposure categories (CED vs. CHUM). For continuous variables, we presented the mean and standard deviation (SD), and for binary variables we presented the count (n) and percentage. Session-specific mean outcomes of all patients from CED and CHUM are presented in Figure S1. The number of patients who switched their dialysis locations are presented in Table S8 by different sessions.

Table S7: Descriptive statistics of data from Session 1.

	Dialysis facility	
**Variables**	CED ($n = 237$)	CHUM ($n = 220$)
**Continuous: Mean (SD)**		
Outcome	27.64 (4.26)	23.41 (6.28)
Hemoglobin	107.31 (14.59)	93.95 (15.78)
Albumin	37.1 (3.75)	33.03 (5.19)
Dalteparin*	47.28 (25.03)	27.36 (21.03)
Age	65.64 (15.02)	68.22 (13.88)
**Binary: n (%)**		
Access catheter	104 (0.44)	171 (0.78)
Alteplase	7 (0.03)	4 (0.02)
Catheter change	1 (0.00)	9 (0.04)
Male	142 (0.60)	141 (0.64)
Hypertension	59 (0.25)	129 (0.59)
Diabetes	40 (0.17)	93 (0.42)
Peripheral vascular disease (pvd)	11 (0.05)	48 (0.22)
Congestive heart failure (chf)	13 (0.05)	45 (0.20)
Cardiac arrhythmia	14 (0.06)	36 (0.16)
Acute myocardial infarction (ami)	11 (0.05)	35 (0.16)
Chronic pulmonary disease (copd)	9 (0.04)	33 (0.15)
Liver disease	11 (0.05)	33 (0.15)
Valvular disease	7 (0.03)	16 (0.07)
Cancer	7 (0.03)	14 (0.06)
Metastatic cancer	1 (0.00)	1 (0.00)
Cerebrovascular disease (cvd)	2 (0.01)	15 (0.07)
Dementia	2 (0.01)	5 (0.02)
Hemiplegia	1 (0.00)	10 (0.05)
Rheumatic disease	4 (0.02)	2 (0.01)

Table S8: Number of patients who switched their locations.

	CHUM to CED	CED to CHUM
Session 2	5	1
Session 3	6	0
Session 4	3	0
Session 5	6	0
Session 6	3	3

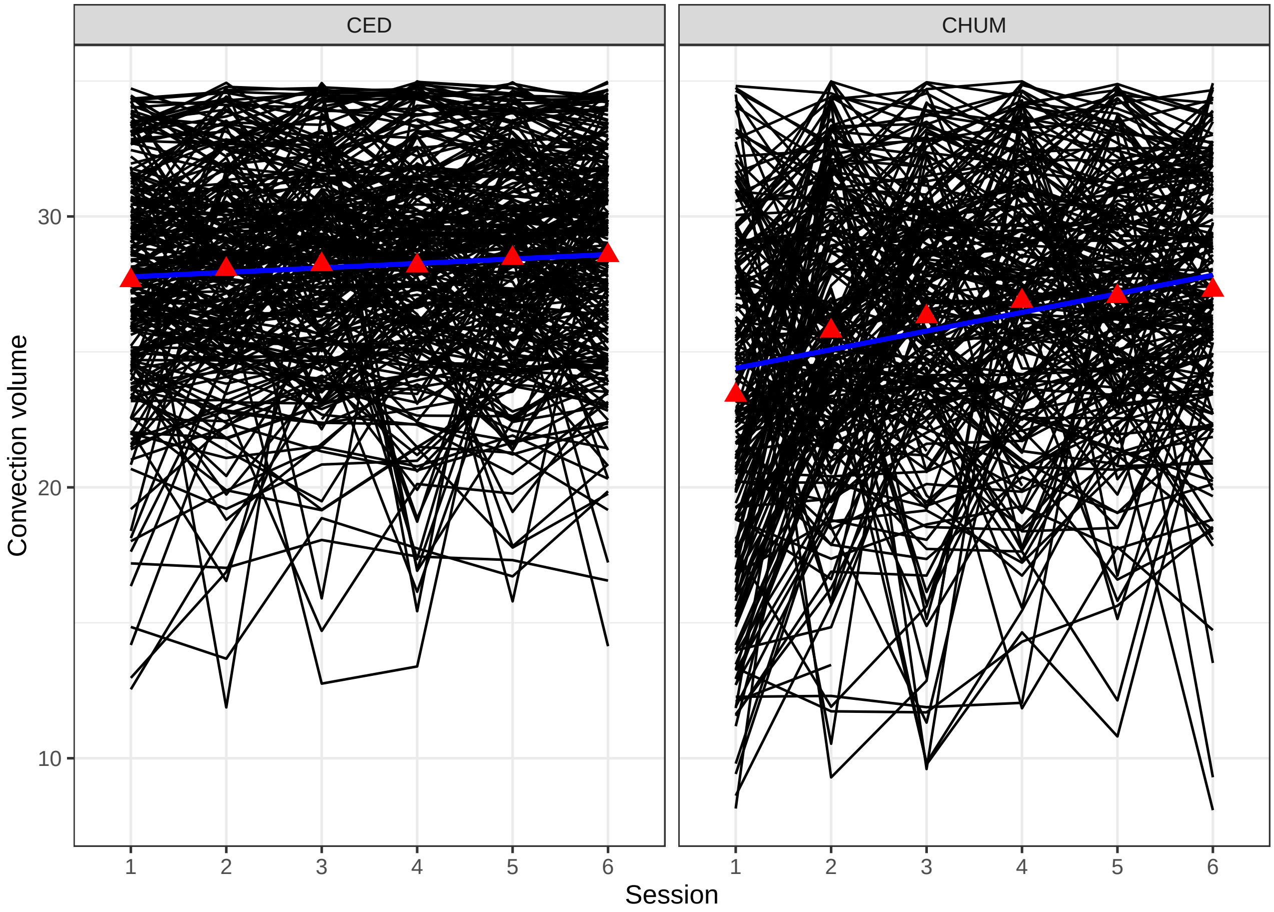

Figure S1: Individual session-specific outcomes (convection volume) of all patients by exposure status (dialysis facility). Red triangles show the session-specific means and the blue lines show the linear fits.

References

Bian, Z., Moodie, E. E., Shortreed, S. M., Lambert, S. D., and Bhatnagar, S. (2023). Variable selection for individualised treatment rules with discrete outcomes. *Journal of the royal statistical society. Series C (Applied statistics)* **qlad096, https://doi.org/10.1093/jrsssc/qlad096,**.

Boruvka, A., Almirall, D., Witkiewitz, K., and Murphy, S. A. (2018). Assessing time-varying causal effect moderation in mobile health. *Journal of the American Statistical Association* **113,** 1112–1121.

Fan, J. and Li, R. (2001). Variable selection via nonconcave penalized likelihood and its oracle properties. *Journal of the American statistical Association* **96,** 1348–1360.

Fan, Y. and Tang, C. Y. (2013). Tuning parameter selection in high dimensional penalized likelihood. *Journal of the Royal Statistical Society Series B: Statistical Methodology* **75,** 531–552.

Johnson, B. A., Lin, D., and Zeng, D. (2008). Penalized estimating functions and variable selection in semiparametric regression models. *Journal of the American Statistical Association* **103,** 672–680.

Moodie, E. E., Bian, Z., Coulombe, J., Lian, Y., Yang, A. Y., and Shortreed, S. M. (2023). Variable selection in high dimensions for discrete-outcome individualized treatment rules: Reducing severity of depression symptoms. *Biostatistics* page kxad022.

Pan, W. (2001). Akaike's information criterion in generalized estimating equations. *Biometrics* **57,** 120–125.

Tibshirani, R. (1996). Regression shrinkage and selection via the lasso. *Journal of the Royal Statistical Society: Series B (Methodological)* **58,** 267–288.

Wahba, G. (1985). A comparison of gcv and gml for choosing the smoothing parameter in the generalized spline smoothing problem. *The annals of statistics* pages 1378–1402.

Wang, H., Li, B., and Leng, C. (2009). Shrinkage tuning parameter selection with a diverging number of parameters. *Journal of the Royal Statistical Society Series B: Statistical Methodology* **71,** 671–683.

Wang, H., Li, R., and Tsai, C.-L. (2007). Tuning parameter selectors for the smoothly clipped absolute deviation method. *Biometrika* **94,** 553–568.

Wang, L., Zhou, J., and Qu, A. (2012). Penalized generalized estimating equations for high-dimensional longitudinal data analysis. *Biometrics* **68,** 353–360.
