# Supplementary material for: Penalized G-estimation for effect modifier selection in a structural nested mean model for repeated outcomes
Source: Biometrics. Author manuscript; Available in PMC 2026 Jun 16. (PMC13271025; doi:10.1093/biomtc/ujae165)
Supplement: Codes [file NIHMS2179184-supplement-Codes.zip › codes_BIOM2024119M/Reproduce simulation tables/Simulations/Boruvka_2018/Assessing Time Varying Causal Effect Moderation in Mobile Health.pdf]

Journal of the American Statistical Association

ISSN: 0162-1459 (Print) 1537-274X (Online) Journal homepage: https://www.tandfonline.com/loi/uasa20

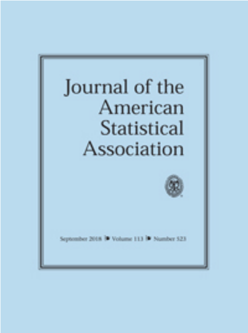

Assessing Time-Varying Causal Effect Moderation in Mobile Health

Audrey Boruvka, Daniel Almirall, Katie Witkiewitz & Susan A. Murphy

To cite this article: Audrey Boruvka, Daniel Almirall, Katie Witkiewitz & Susan A. Murphy (2018) Assessing Time-Varying Causal Effect Moderation in Mobile Health, Journal of the American Statistical Association, 113:523, 1112-1121, DOI: 10.1080/01621459.2017.1305274

To link to this article: https://doi.org/10.1080/01621459.2017.1305274

View supplementary material

Published online: 08 Oct 2018.

Submit your article to this journal

Article views: 2311

View related articles

View Crossmark data

Citing articles: 30 View citing articles

Full Terms & Conditions of access and use can be found at
https://www.tandfonline.com/action/journalInformation?journalCode=uasa20

JOURNAL OF THE AMERICAN STATISTICAL ASSOCIATION
2018, VOL. 113, NO. 523, 1112–1121: Theory and Methods
https://doi.org/10.1080/01621459.2017.1305274

Taylor & Francis
Taylor & Francis Group

Check for updates

Assessing Time-Varying Causal Effect Moderation in Mobile Health

Audrey Boruvka[a], Daniel Almirall[b], Katie Witkiewitz[c], and Susan A. Murphy[a,b]

[a]Department of Statistics, University of Michigan, Ann Arbor, MI; [b]Institute for Social Research, University of Michigan, Ann Arbor, MI; [c]Department of Psychology, University of New Mexico, Albuquerque, NM

ABSTRACT
In mobile health interventions aimed at behavior change and maintenance, treatments are provided in real time to manage current or impending high-risk situations or promote healthy behaviors in near real time. Currently there is great scientific interest in developing data analysis approaches to guide the development of mobile interventions. In particular data from mobile health studies might be used to examine effect moderators—individual characteristics, time-varying context, or past treatment response that moderate the effect of current treatment on a subsequent response. This article introduces a formal definition for moderated effects in terms of potential outcomes, a definition that is particularly suited to mobile interventions, where treatment occasions are numerous, individuals are not always available for treatment, and potential moderators might be influenced by past treatment. Methods for estimating moderated effects are developed and compared. The proposed approach is illustrated using BASICS-Mobile, a smartphone-based intervention designed to curb heavy drinking and smoking among college students. Supplementary materials for this article are available online.

ARTICLE HISTORY
Received January 2016
Revised January 2017

KEYWORDS
Effect modification; mHealth; Structural nested mean model

1. Introduction

Mobile health (mHealth) broadly refers to the practice of healthcare using mobile devices, such as smartphones and wearable sensors both to deliver treatment as well as to sense the current context of the individual. In mobile interventions for behavior maintenance or change, treatments are typically designed to help individuals manage high-risk situations or promote healthy behaviors. Examples include medication reminders, motivational messages, physical activity suggestions, cognitive exercises to help manage stress or other risky situations, and prompts to facilitate activity in support networks.

There is intense interest in data analysis approaches to guide the development of mobile interventions (Free et al. 2013; Muessig et al. 2013) and to test the dynamic behavioral theories on which these interventions are based (Spring et al. 2013; Mohr et al. 2014). Micro-randomized trials (MRTs; Klasnja et al. 2015; Liao et al. 2015; Dempsey et al. 2015) provide data expressly for this purpose, with each participant in an MRT sequentially randomized to treatment numerous times, at possibly 100s to 1000s of occasions. In both MRTs and observational mHealth studies both treatment and measurement occur intensively over time. Measurements on individual characteristics, context, and response to treatments are collected passively through sensors or actively by self-report.

One way in which these data may aid the design of a mobile intervention is through the examination of effect moderation, that is, inference about which factors strengthen or weaken the response to treatments. Consider, for example, an intervention for smoking cessation. Mindfulness-based treatments to help individuals manage their urge to smoke are presumably best delivered at times when there exists an inclination to smoke (e.g., Witkiewitz et al. 2014). However, other factors might influence the effect of these treatments on subsequent smoking rate. For example, it may be that the mindfulness-based approach reduces smoking only when stress levels or self-regulatory demands are low, and has little to no effect otherwise. In general, knowledge about moderators can be used to deliver treatments only in settings where they have proven most efficacious or to identify alternative treatment strategies when the treatment shows little to no benefit. Treatment effects might also evolve over the course of the intervention, so functions of time could also be examined as possible moderators.

This article provides two main contributions in the assessment of treatment effects from longitudinal data in which treatment, response, and potential moderators are time-varying. The first is a definition for treatment effects that is particularly suited for mHealth, where treatment occasions are numerous and potential moderators might be influenced by past treatment. These effects are a marginal generalization of the treatment "blips" in the structural nested mean model (SNMM; Robins 1989, 1994, 1997); the effects are conditional on a few select variables representing potential moderators of interest as opposed to requiring that the effects be conditional on all past observed variables. The second contribution is a centered and weighted least-square method for estimating these treatment effects.

The most common estimation methods used in the analysis of mobile health data are generalized estimating equation (GEE) approaches or related approaches that employ random effects (Schafer 2006; Schwartz and Stone 2007; Bolger and Laurenceau 2013); these methods are frequently used to better understand

CONTACT Daniel Almirall dalmiral@umich.edu Institute for Social Research, University of Michigan, 426 Thompson St., #2204, Ann Arbor, MI 48104.
Supplementary materials for this article are available online. Please go to www.tandfonline.com/r/JASA.
© 2018 American Statistical Association

the time-varying relationship between two variables such as craving and stress. Unfortunately, when the mobile health data includes time-varying treatment, these methods are not guaranteed to consistently estimate causal treatment effects. In this article, we provide a centered and weighted least-square estimation method that provides unbiased estimation.

We begin by defining treatment effects in our setting. The centered and weighted estimation method is derived and its properties are assessed numerically using a variety of simulation scenarios. As an illustration, we apply the proposed method to data from a study of BASICS-Mobile, a mobile intervention to curb heavy drinking and smoking among college students (Witkiewitz et al. 2014).

2. Proximal and Other Lagged Treatment Effects

2.1. Motivating Example

Our motivating example is drawn from BASICS-Mobile, a smartphone-based intervention designed to reduce heavy drinking and smoking among college students. Users are prompted three times per day (morning, afternoon, and evening) to complete a self-report assessing a variety of individual and contextual factors including episodes of drinking or smoking, social settings, affect, and need to self-regulate thoughts. The afternoon and evening self-reports are possibly followed by a treatment module of three to four screens of information and at least one question to confirm that the module was received. Some of the treatment modules address smoking and heavy drinking using mindfulness messages (Bowen and Marlatt 2009). Other modules provide general (primarily health-related) information (Dimeff 1999). In an analysis of data arising from the implementation of BASICS-Mobile, it is natural to estimate the effect of providing the mindfulness messages (vs. providing general health information) on a proximal response, such as the smoking rate between the current and following self-report, and to assess whether or not these effects differ according to the individual's context.

2.2. Notation and Data

For a given individual, let A_t denote the treatment at the tth treatment occasion and Y_{t+1} be the subsequent proximal response ($t = 1, \ldots, T$). Throughout we limit attention to the case where each A_t is binary and Y_{t+1} is continuous. Individual and contextual information at the tth treatment occasion is represented by X_t, which may contain summaries of previous measurements of context, treatment, or response. For example, prior to each treatment occasion, the individual might report their current mood. The vector X_t could then contain this measurement or, with previous measurements, variation or change in mood. Over the course of T treatment occasions, the resulting data from an individual ordered in time is $(X_1, A_1, Y_2, \ldots, X_T, A_T, Y_{T+1})$. The overbar is used to denote a sequence of random variables or realized values through a specific treatment occasion, for example, $\bar{A}_t = (A_1, \ldots, A_t)$. Information accrued up to treatment occasion t is represented by the history $H_t = (\bar{X}_t, \bar{Y}_t, \bar{A}_{t-1})$. Throughout, we represent

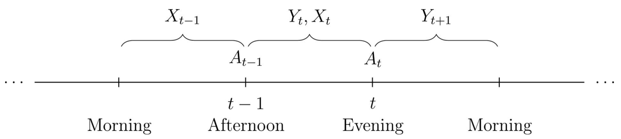

Figure 1. A BASICS-Mobile participant's data for two treatment occasions leading up to Y_{t+1}, depicted in chronological order. Information is primarily collected via self-reports three times per day—morning, afternoon, and evening. Treatment occasions take place after the afternoon and evening self-reports.

random variables or vectors with uppercase letters; lowercase letters denote their realized values.

In BASICS-Mobile (Figure 1), $A_t = 1$ if a mindfulness message is provided at the tth treatment occasion and $A_t = 0$ otherwise, Y_{t+1} is the smoking rate between the occasion t self-report prompt and the following self-report prompt, $T = 28$, and X_t includes the time of day, number of reports recently completed, prior smoking rate, current need to self-regulate, and other summary variables formed from the reports up to and including the tth occasion. For example, from the self-reports at $t-1$ and t, we can examine the change in self-regulation needs and determine whether there was an increased need ($\mathrm{incr_t} = 1$) or not ($\mathrm{incr_t} = 0$).

In the following section, we define the causal effects of interest in terms of the potential outcomes. Then we express the causal effects in terms of the observed data and provide causal assumptions sufficient for these expressions.

2.3. Moderated Treatment Effects

To define treatment effects below, we adopt potential outcomes (Rubin 1974; Robins 1989; Neyman 1990) notation. However, we will deviate slightly from this framework because, as will be seen below in (2), our estimands may involve the treatment distribution in the data. In particular, it will be useful to include in the set of potential outcomes, treatments expressed as potential outcomes of past treatment. That is, the potential outcomes are $\{Y_2(a_1), X_2(a_1), A_2(a_1)\}_{a_1 \in \{0,1\}}, \ldots, \{Y_T(\bar{a}_{T-1}), X_T(\bar{a}_{T-1}), A_T(\bar{a}_{T-1})\}_{\bar{a}_{T-1} \in \{0,1\}^{T-1}}, \{Y_{T+1}(\bar{a}_T)\}_{\bar{a}_T \in \{0,1\}^T}$. In BASICS-Mobile, for example, the smoking rate measured following the second treatment occasion has four potential outcomes: $Y_3(0,0)$, $Y_3(0,1)$, $Y_3(1,0)$, $Y_3(1,1)$. Here, $Y_3(0,0)$ is the smoking rate that would arise for a given individual had that individual received no mindfulness treatments over the first two treatment occasions: $a_1 = a_2 = 0$. This idea can be similarly applied to the measurements X_t, since they might also be influenced by past treatment; $X_{t+1}(\bar{a}_t)$ are the potential measurements had the sequence of treatments $\bar{a}_t$ been allocated. For brevity, we denote $A_2(A_1)$ by A_2 and so on with $A_t(\bar{A}_{t-1})$ denoted by A_t. Then $H_t(\bar{A}_{t-1}) = X_1, A_1, Y_2(A_1), X_2(A_1), A_2, Y_3(\bar{A}_2), X_3(\bar{A}_2), A_3, \ldots, Y_t(\bar{A}_{t-1}), X_t(\bar{A}_{t-1})$.

Many treatments are designed to influence an individual in the short term or proximally in time (Heron and Smyth 2010). For example, instruction in the mindfulness intervention used in BASICS-Mobile, called urge surfing, aims to help the individual to "ride out" urges, by recognizing the urge as it arises and allowing the urge to pass on its own. Questions related to these effects concern the proximal effect of treatment on the response

defined by

$$\mathrm{E}[Y_{t+1}(\bar{A}_{t-1}, 1) - Y_{t+1}(\bar{A}_{t-1}, 0) \mid S_{1t}(\bar{A}_{t-1})], \quad (1)$$

where $S_{1t}(\bar{A}_{t-1})$ is a vector of summary variables chosen from $H_t(\bar{A}_{t-1})$. The difference in (1) represents the effect of $A_t = 1$ versus $A_t = 0$ on the response at $t + 1$, given $S_{1t}(\bar{A}_{t-1})$. In conditioning only on $S_{1t}(\bar{A}_{t-1})$ as opposed to $H_t(\bar{A}_{t-1})$, the effect (1) is marginalized over variables in $H_t(\bar{A}_{t-1})$ that are not in $S_{1t}(\bar{A}_{t-1})$. Different choices of variables in S_{1t} address a variety of scientific questions, each of which is useful for understanding the effect of $A_t = 1$ versus $A_t = 0$ on the response Y_{t+1}. For example, a first analysis may focus on the proximal effect that is marginal over all variables in $H_t(\bar{A}_{t-1})$ (i.e., $S_{1t} = \emptyset$), whereas a second analysis may focus on assessing this effect conditional on particular variables from $H_t(\bar{A}_{t-1})$.

Note that, for any A_u not contained in $S_{1t}(\bar{A}_{t-1})$, the expectation in (1) depends on distribution of A_u. This is a departure from the causal inference literature, where estimands do not depend on the treatment distribution in the data at hand. Nonetheless, for all choices of variables in $S_{1t}(\bar{A}_{t-1})$, the proximal treatment effect is causal, since (1) is the conditional mean of the contrast between the potential proximal response had an individual received ($a_t = 1$) versus not received ($a_t = 0$) treatment at occasion t. Considering the dependence of the proximal effect on the distribution of the treatments, it is best to always present this distribution along with the estimated treatment effect. For further discussion concerning including the treatment distribution as part of the estimand, see Section 8.

Many treatments may have delayed effects. For example, mindfulness messages have a delayed effect when individuals recall and employ mindfulness exercises provided prior to the most recent treatment occasion. In BASICS-Mobile, treatments suggesting alternative activities to smoking and drinking may achieve little to no immediate impact in the afternoon, but the individual might follow these suggestions later on in the evening. So in general both proximal and other lagged effects of treatments on the response variable may be of interest. To define these lagged effects, we denote $A_{t+1}(\bar{A}_{t-1}, a)$ by $A_{t+1}^{a_t=a}$, $A_{t+2}(\bar{A}_{t-1}, a, A_{t+1}^{a_t=a})$ by $A_{t+2}^{a_t=a}$ and so on, with $A_{t+k-1}(\bar{A}_{t-1}, a, A_{t+1}^{a_t}, \ldots, A_{t+k-2}^{a_t=a})$ by $A_{t+k-1}^{a_t=a}$. We define the lag k effect of treatment on the response k treatment occasions into the future Y_{t+k} by

$$\begin{aligned}\mathrm{E}\Big[&Y_{t+k}\left(\bar{A}_{t-1}, 1, A_{t+1}^{a_t=1}, \ldots, A_{t+k-1}^{a_t=1}\right)\\ &-Y_{t+k}\left(\bar{A}_{t-1}, 0, A_{t+1}^{a_t=0}, \ldots, A_{t+k-1}^{a_t=0}\right) \mid S_{kt}(\bar{A}_{t-1})\Big], \quad (2)\end{aligned}$$

where k ranges from 1 up to the number of lags of scientific interest. So the proximal effect (1) corresponds to the lag $k = 1$ treatment effect. Note that both future actions, as well as Y_{t+k}, depend on treatment at occasion t as emphasized by the superscripts $a_t = 1$ or $a_t = 0$. As with (1), $S_{kt}(\bar{A}_{t-1})$ is a vector of variables from the history $H_t(\bar{A}_{t-1})$. S_{kt} is indexed by k to allow for the possibility that scientists may be interested in assessing effect moderation by different variables depending on the lag k. For example, current busyness might be expected to moderate the proximal ($k = 1$) effect of treatment, whereas expected busyness over the remaining day might be expected to moderate more delayed ($k > 1$) effects. The lagged effect is also similarly averaged over the conditional distribution of variables in the history $H_t(\bar{A}_{t-1})$ not represented in $S_{kt}(\bar{A}_{t-1})$, which might include past treatment or underlying moderators. In addition, (2) is averaged over the distribution of treatments after occasion t but before response Y_{t+k}—namely, $A_{t+1}^{a_t=a}, \ldots, A_{t+k-1}^{a_t=a}$ for either $a = 1$ or $a = 0$.

The causal effect in (2) is a generalization of the treatment "blip" in the SNMM. In SNMMs, the tth treatment blip or intermediate effect on Y_{t+k} is usually defined with $S_{kt}(\bar{A}_{t-1}) = H_{kt}(\bar{A}_{t-1})$ and with respect to a prespecified future (after time t) "reference" treatment regime that defines the distribution for $A_{t+1}, \ldots, A_{t+k-1}$. For example, if we were studying treatment discontinuation, we might have chosen the reference regime $A_u = 0$ for $u > t$, with probability one (see Robins 1994, sec. 3a). In this case, the lag k treatment effect (2) represents the impact of one last additional treatment on the proximal response k time units later. The reference treatment regime reflected in (2), however, assigns treatment with probabilities between zero and one and corresponds to the distribution of treatments in the data we have at hand. For further discussion of the connection between the causal effects defined here and the SNMM, see Supplement A.1.

To express the proximal and other lagged effects in terms of the observed data, we assume positivity, consistency, and sequential ignorability (Robins 1994, 1997):

- Consistency: The observed data $(Y_2, X_2, A_2, \ldots, Y_T, X_T, A_T, Y_{T+1})$ are equal to the potential outcomes as follows: $Y_2 = Y_2(A_1), X_2 = X_2(A_1), A_2 = A_2(A_1)$ and for each subsequent $t \le T$, $Y_t = Y_t(\bar{A}_{t-1})$, $X_t = X_t(\bar{A}_{t-1}), A_t = A_t(\bar{A}_{t-1})$ and finally $Y_{T+1} = Y_{T+1}(\bar{A}_T)$.
- Positivity: If the joint density at $\{H_t = h_t, A_t = a_t\}$ is greater than zero, then $\Pr(A_t = a_t \mid H_t = h_t) > 0$, almost everywhere.
- Sequential ignorability: For each $t \le T$, the potential outcomes $\{Y_{t+1}(\bar{a}_t), X_{t+1}(\bar{a}_t), A_{t+1}(\bar{a}_t), \ldots, Y_{T+1}(\bar{a}_T)\}$ are independent of A_t conditional on H_t.

The consistency assumption connects the potential outcomes with the data. When the treatment allocated to one individual may influence the response of others, the observed response Y_{t+1} is generally consistent not with the potential response $Y_{t+1}(\bar{A}_t)$ as above, but possibly with some other group-based conceptualization (e.g., Hong and Raudenbush 2006; Vanderweele et al. 2013). In particular, for a mobile intervention with a social media component, it may be necessary to define the potential outcomes for a given individual as a function of the treatments that are provided to individuals in their social network.

In an MRT, treatment is sequentially randomized according to known treatment probabilities, say $\Pr(A_t = 1 \mid H_t) = p_t(1 \mid H_t)$, $t = 1, \ldots, T$, and thus sequential ignorability is ensured by design. In an observational study, where treatment status is observed rather than randomized, sequential ignorability is often assumed. Here the underlying treatment probabilities $p_t(1 \mid H_t), t = 1, \ldots, T$, are unknown.

In Supplement A.2, we show that, under these assumptions, the lag k treatment effect can be expressed in terms of the

observed data as

$$\mathrm{E}\Bigg[Y_{t+k}\left(\bar{A}_{t-1}, 1, A_{t+1}^{a_t=1}, \ldots, A_{t+k-1}^{a_t=1}\right) - Y_{t+k}\left(\bar{A}_{t-1}, 0, A_{t+1}^{a_t=0}, \ldots, A_{t+k-1}^{a_t=0}\right) \,\Big|\, S_{kt}(\bar{A}_{t-1})\Bigg]$$
$$= \mathrm{E}\left[\mathrm{E}[Y_{t+k} \mid A_t = 1, H_t] - \mathrm{E}[Y_{t+k} \mid A_t = 0, H_t] \mid S_{kt}\right]$$
$$= \mathrm{E}\left[\frac{1(A_t = 1)Y_{t+k}}{p_t(1 \mid H_t)} - \frac{1(A_t = 0)Y_{t+k}}{1 - p_t(1 \mid H_t)} \,\Big|\, S_{kt}\right], \quad (3)$$

for $t = 1, \ldots, T - k + 1$, respectively. Note that if $S_{kt} = H_t$, then the lag k effect simplifies to

$$\mathrm{E}[Y_{t+k} \mid A_t = 1, H_t] - \mathrm{E}[Y_{t+k} \mid A_t = 0, H_t]. \quad (4)$$

3. Estimation

In the following, we assume a linear model for the treatment effects. Fortunately, models for the proximal and other lagged treatment effects can in fact be specified separately, since (2) for differing lags k do not constrain one another (Robins 1994, 1997; see Supplement B). Suppose that the following holds.

A1 Each lag k treatment effect of interest takes the form

$$\mathrm{E}[\mathrm{E}[Y_{t+k} \mid A_t = 1, H_t] - \mathrm{E}[Y_{t+k} \mid A_t = 0, H_t] \mid S_{kt}] = f_{kt}(S_{kt})^{\mathrm{T}}\beta_k, \quad (5)$$

where $f_{kt}(s)$ is a p-dimensional vector function of s and time t.

Note that (5) does not imply that the lag-k effect is the same over time; indeed, the vector $f_{kt}(S_{kt})$ may include a vector of basis functions in time, for example, for modeling time-varying effects. When $S_{kt} \neq H_t$, (5) is a marginal model. For example, if $S_{kt} = \emptyset$, then (5) is $\mathrm{E}[\mathrm{E}[Y_{t+k} \mid A_t = 1, H_t] - \mathrm{E}[Y_{t+k} \mid A_t = 0, H_t]] = f_{kt}^{\mathrm{T}}\beta_k$, which is a model for the lag k treatment effects indexed by t but marginal over H_t.

The rest of this article is devoted to inference on the unknown p-dimensional β_k. Throughout, we denote the true value of β_k by β_k^*, n represents the number of individuals in the data, and $\mathbb{P}_n h(Z) = \sum_{i=1}^n h(Z_i)/n$ for some function h of the random vector Z. Assume the data come from an MRT; in this case sequential ignorability is satisfied. In particular, we assume

A2 Treatment is sequentially randomized with randomization probability $\Pr(A_t = 1 \mid H_t) = p_t(1 \mid H_t)$, for each $t = 1, \ldots, T$.

Inference concerning β_k using data from observational studies in which the treatment is not sequentially randomized can be handled—if the assumption of sequential ignorability holds—by estimating the treatment probability; see Supplement C.

The following, simple, estimation method includes centering of the treatment indicators and weighting of the estimating function. The weights allow us to estimate marginal treatment effects, for example, conditional on S_{kt} instead of H_t. As discussed above this commonly occurs, for example, when interest lies in the treatment effect of A_t for $S_{kt} = \emptyset$. The weights are ratios of probabilities, with the denominator weight equal to the randomization probability; the numerator probability is arbitrary as long as this probability depends on H_t only via S_{kt} (the variables in the treatment effect model, (5)). Denote the numerator probabilities by, $\tilde{p}_t(a|S_{kt})$ for $t = 1, \ldots, T$. The weight at occasion t is $W_t = \frac{\tilde{p}_t(A_t|S_{kt})}{p_t(A_t|H_t)}$.

The centering produces orthogonality between estimation of the β_k parameter in the treatment effect, $f_{kt}(S_{kt})^{\mathrm{T}}\beta_k$ and estimation of the parameters in a nuisance function. That is, the method below will provide a consistent estimator of the lag k effect even when the nuisance function $\mathrm{E}[W_t Y_{t+k} \mid H_t]$ is misspecified. This robustness property is desirable for two reasons. First, the history H_t is usually high dimensional, making it very difficult to model these nuisance functions correctly. Second, even when H_t is not very large, it can be difficult or impossible to specify models that can be correct for both the nuisance function as well as for the delayed treatment effects at lags $j > k$ (see Supplement B for an example). Below we provide results when the working model for $\mathrm{E}[W_t Y_{t+k} \mid H_t]$ is $g_{kt}(H_t)^{\mathrm{T}}\alpha_k$ where $g_{kt}(H_t)$ is a vector of features constructed from H_t and the vector α_k is unknown.

The centered and weighted least-square estimating function is

$$U_{\mathrm{W}}(\alpha_k, \beta_k) = \sum_{t=1}^{T-k+1} \left(Y_{t+k} - g_{kt}(H_t)^{\mathrm{T}}\alpha_k - (A_t - \tilde{p}_t(1 \mid S_{kt}))f_{kt}(S_{kt})^{\mathrm{T}}\beta_k\right) W_t \begin{pmatrix} g_{kt}(H_t) \\ (A_t - \tilde{p}_t(1 \mid S_{kt}))f_{kt}(S_{kt}) \end{pmatrix}, \quad (6)$$

where as before, $W_t = \frac{\tilde{p}_t(A_t|S_{kt})}{p_t(A_t|H_t)}$. Let $\dot{U}_{\mathrm{W}}$ be the derivative of U_{W} with respect to the row vector $(\alpha_k^{\mathrm{T}}, \beta_k^{\mathrm{T}})$. In Supplement C, we prove a more general version of the following result.

Proposition 1. Assume A1 and A2, both defined above. Then, under invertibility and moment conditions, the solution to the estimating equation $\mathbb{P}_n U_{\mathrm{W}}(\alpha_k, \beta_k) = 0$ yields an estimator $(\hat{\alpha}_k, \hat{\beta}_k)$ for which $\sqrt{n}(\hat{\beta}_k - \beta_k^*)$ is asymptotically normal with mean zero and variance-covariance matrix consistently estimated by the lower block diagonal $(p \times p)$ entry of the matrix $(\mathbb{P}_n \dot{U}_{\mathrm{W}}(\hat{\alpha}_k, \hat{\beta}_k))^{-1} \mathbb{P}_n U_{\mathrm{W}}(\hat{\alpha}_k, \hat{\beta}_k)^{\otimes 2} (\mathbb{P}_n \dot{U}_{\mathrm{W}}(\hat{\alpha}_k, \hat{\beta}_k))^{-1^{\mathrm{T}}}$.

Remarks

1. A first look at the estimating function, (6), might lead one to think that the estimating function is unbiased only if $\mathrm{E}[Y_{t+k} \mid A_t, H_t] = g_{kt}(H_t)^{\mathrm{T}}\alpha_k + (A_t - \tilde{p}_t(1 \mid S_{kt}))f_{kt}(S_{kt})^{\mathrm{T}}\beta_k$ for some (α_k, β_k); however this is not the case. Indeed, the primary assumption A1 only concerns a marginal quantity derived from $\mathrm{E}[Y_{t+k} \mid A_t, H_t]$. Furthermore, the working model $g_{kt}(H_t)^{\mathrm{T}}\alpha_k$ for $\mathrm{E}[W_t Y_{t+k} \mid H_t]$ need not be correct in order for $\hat{\beta}_k$ to be consistent and for the large sample results to hold (see the proof in Supplement C).
2. As mentioned above, the choice of the numerator of the weight, $\tilde{p}_t$ is arbitrary as long as $\tilde{p}_t$ depends at most on S_{kt}. One approach to selecting $\tilde{p}_t$ is to recognize that $\tilde{p}_t$ determines the estimand when the model for the treatment effect in (5) is misspecified. See (14, 15) in Supplement C for the projection. In particular, selecting $\tilde{p}_t$ to

be constant in t and S_{kt} results in the usual L_2-projection of underlying treatment effect.

3. It is interesting to note that if the randomization probabilities are constant, ρ, then setting $\tilde{p}_t(1|S_{kt}) = \rho$, simplifies (6) to an unweighted regression with recoded treatment indicators ($A_t \to A_t - \rho$).
4. The weight W_t is reminiscent of inverse probability of treatment weighting in causal inference (Robins 1998). However, in addition to facilitating estimation of marginal treatment effects, here weighting (and centering) is simply used to make the weighted least-square estimator $\hat{\beta}_k$ robust against the case in which the working model $g_{kt}(H_t)^{\mathrm{T}}\alpha_k$ misspecifies $\mathrm{E}[W_t Y_{t+k} \mid H_t]$. Further, this similarity might lead one to use the numerator of the weight to "stabilize" the weights (e.g., sec. 6.1 of Robins, Hernán, and Brumback 2000), that is, to select a $\tilde{p}_t$ to make W_t as close to 1 as possible. There are two caveats to this. First, as mentioned in remark 2 above, the numerator probabilities determine the limit of $\hat{\beta}_k$ when the modeling assumption for the lag k treatment effect (5) is false and thus might be selected with this alternative interpretation for the estimand in mind. Second, bias can result if the numerator of the weight depends on variables that are not in S_{kt}; see the second simulation in Section 6.
5. Centering has been previously employed by Brumback et al. (2003) and Goetgeluk and Vansteelandt (2008) for causal inference. For example, Goetgeluk and Vansteelandt (2008) centered exposure variables by their overall mean to protect against unmeasured baseline confounders. Brumback et al. (2003) centered time-varying exposures by their conditional mean given the history, as we do; they consider treatment effects under a treatment discontinuation reference regime and limit attention to overall effects without interaction terms. In contrast to these articles, our use of centering is similar to that of Liao et al. (2015) and is solely to provide robustness to the working model for $\mathrm{E}[W_t Y_{t+k} \mid H_t]$; centering is not used to adjust for confounding. In Liao et al. (2015), the treatment probabilities are nonstochastic.
6. The similarity of (6) to generalized estimating equations (GEEs, Liang and Zeger 1986) might motivate the inclusion of a nonindependence working correlation matrices such as exchangeable or AR(1) in the estimating function so as to reduce variance of $\hat{\beta}_k$ (e.g., Mancl and Leroux 1996). Similarly, an analyst might wish to use a nonindependence working correlation matrix in our setting for the same reason, but this strategy will generally introduce bias. Such a result is unsurprising given the bias that arises when nonindependence working matrices are used in inverse probability of treatment weighting literature (Vansteelandt 2007; Tchetgen Tchetgen et al. 2012) or in GEEs where a time-varying response is modeled by time-varying covariates (Pepe and Anderson 1994). The simulations in Table 3 in Section 6, and Table 7 in Supplement D illustrate such bias.

4. Availability

Up to this point we have implicitly presumed that at every possible occasion t, the participant is available to engage with the mobile intervention. Consideration of availability is critical since it might be unreasonable, counter-productive, or even unethical to always presume availability. By experimental design, treatment will not be delivered to unavailable individuals. For example, in HeartSteps (Klasnja et al. 2015), smartphone notifications are used to deliver suggestions to disrupt sedentary behavior. Here, the participant is considered unavailable when driving a vehicle (because the notification may be distracting) or walking (as treatment at this time is scientifically inappropriate). Detection of availability can be carried out through sensors (as in the case of HeartSteps) or recent interaction with the mobile device. BASICS-Mobile took the latter approach by presuming that participants were available to receive a treatment only after they fully completed a self-report.

Assume that the measurements X_t just prior to the tth treatment occasion contain the participant's availability status, denoted by I_t, where $I_t = 1$ if the participant is available to engage with the treatment at occasion t and $I_t = 0$ otherwise. To define the treatment effects under limited availability, we use potential outcome notation. The potential outcome notation allows us to not only make explicit the dependence of Y_{t+1} on treatment $\bar{a}_t$ but also make explicit the dependence of I_t on $\bar{a}_{t-1}$. Furthermore, in contrast to Section 2.3, here the potential outcomes are indexed by decision rules because treatment can only be provided when a participant is available. The use of decision rules to index potential outcomes helps make explicit that, by experimental design, treatment A_t is not delivered if the participant is unavailable at the t treatment occasion. In particular, define $d(a, i)$ for $a \in \{0, 1\}$, $i \in \{0, 1\}$ by $d(a, 0) = 0$ and $d(a, 1) = a$ (recall that here $a = 0$ means no treatment). Then for each $a_1 \in \{0, 1\}$, define $D_1(a_1) = d(a_1, I_1)$. The potential proximal responses following treatment occasion 1 are $\{Y_2(D_1(1)), Y_2(D_1(0))\}$. Note that if $I_1 = 0$ then $D_1(1) = D_1(0) = 0$ and thus $\{Y_2(D_1(1)), Y_2(D_1(0))\} = \{Y_2(0), Y_2(0)\}$. That is, the experimental design excludes the possibility to observe $Y_2(1)$ if $I_1 = 0$. Similarly, there are potential outcomes for availability; this emphasizes the fact that previous exposure to treatment can influence subsequent availability. In BASICS-Mobile, for example, repeated provision of treatment might lead to lower engagement with the intervention, and therefore lower availability for further delivery of the treatment. The potential availability indicators at $t = 2$ are $\{I_2(D_1(1)), I_2(D_1(0))\}$. As with the proximal response, if $I_1 = 0$ then $D_1(1) = D_1(0) = 0$ and thus $\{I_2(D_1(1)), I_2(D_1(0))\} = \{I_2(0), I_2(0)\}$.

The decision rules at $t > 1$ are defined iteratively, building on prior decision rules. For each $\bar{a}_2 = (a_1, a_2)$ with $a_1, a_2 \in \{0, 1\}$, define $D_2(\bar{a}_2) = d(a_2, I_2(D_1(a_1)))$ and $\overline{D_2(\bar{a}_2)} = (D_1(a_1), D_2(\bar{a}_2))$. A potential proximal response following occasion $t = 2$ and corresponding to $\bar{a}_2$ is $Y_3(\overline{D_2(\bar{a}_2)})$ and a potential availability indicator at $t = 3$ is $I_3(\overline{D_2(\bar{a}_2)})$. Similarly, for each $\bar{a}_t = (a_1, \ldots, a_t) \in \{0, 1\}^t$, define $D_t(\bar{a}_t) = d(a_t, I_t(\overline{D_{t-1}(\bar{a}_{t-1})}))$ and $\overline{D_t(\bar{a}_t)} = (D_1(a_1), \ldots, D_t(\bar{a}_t))$. For each $\bar{a}_t = (a_1, \ldots, a_t) \in \{0, 1\}^t$, the potential proximal response is $Y_{t+1}(\overline{D_t(\bar{a}_t)})$ and potential availability indicator is $I_{t+1}(\overline{D_t(\bar{a}_t)})$ at occasion $t + 1$.

We now incorporate availability into the definition of the proximal treatment effect; first recall the notation from the end of Section 2.2; similarly denote $A_2(D_1(A_1))$ by A_2 and so on with $A_t(\overline{D_{t-1}(\bar{A}_{t-1})})$ denoted by A_t. The proximal treatment effect is

$$E\left[Y_{t+1}(\overline{D_t(\bar{A}_{t-1},1)}) - Y_{t+1}(\overline{D_t(\bar{A}_{t-1},0)}) \mid I_t(\overline{D_{t-1}(\bar{A}_{t-1})}) = 1, S_{1t}(\overline{D_{t-1}(\bar{A}_{t-1})})\right].$$

Unlike (1), this effect is defined for only individuals available for treatment at time t, that is, $I_t(\overline{D_{t-1}(\bar{A}_{t-1})}) = 1$. This subpopulation is not static; at a given treatment occasion t only certain types of individuals might tend to be available and availability for any given individual may change with t. Conditioning on availability is related to the concept of viable or feasible dynamic treatment regimes (Robins 2004; Wang et al. 2012), in which one assesses only the causal effect of treatments that can actually be provided.

To incorporate availability into the definition of the lagged effects, we use the shorthand notation: denote $A_{t+1}(\overline{D_t(\bar{A}_{t-1},a)})$ by $A_{t+1}^{a_t=a}$, $A_{t+2}(\overline{D_{t+1}(\bar{A}_{t-1},a), A_{t+1}^{a_t=a}})$ by $A_{t+2}^{a_t=a}$, and so on, with $A_{t+k-1}(\overline{D_{t+1}(\bar{A}_{t-1},a), A_{t+1}^{a_t}, \ldots, A_{t+k-2}^{a_t=a}})$ by $A_{t+k-1}^{a_t=a}$. The lag k effect of treatment on the response k treatment occasions into the future Y_{t+k} is defined by

$$E\Big[Y_{t+k}\left(\overline{D_t(\bar{A}_{t-1},1), A_{t+1}^{a_t=1}, \ldots, A_{t+k-1}^{a_t=1}}\right) - Y_{t+k}\left(\overline{D_t(\bar{A}_{t-1},0), A_{t+1}^{a_t=0}, \ldots, A_{t+k-1}^{a_t=0}}\right) \mid S_{kt}(\overline{D_{t-1}(\bar{A}_{t-1})})\Big].$$

Assuming consistency, positivity, and sequential ignorability, the lag k treatment effect under limited availability can be expressed in terms of the data as

$$E\left[E[Y_{t+k} \mid A_t = 1, I_t = 1, H_t] - E[Y_{t+k} \mid A_t = 0, I_t = 1, H_t] \times \mid I_t = 1, S_{kt}\right] = E\left[\frac{1(A_t = 1)Y_{t+1}}{p_t(1 \mid H_t)} - \frac{1(A_t = 0)Y_{t+1}}{1 - p_t(1 \mid H_t)} \mid I_t = 1, S_{kt}\right],$$

where $p_t(1 \mid H_t)$ is now $\Pr(A_t = 1 \mid I_t = 1, H_t)$. Modeling and estimation proceeds following the same approach as with the always-available setting. In particular, for the lag k treatment effect, we assume the linear model

$$E\big[E[Y_{t+k} \mid A_t = 1, I_t = 1, H_t] - E[Y_{t+k} \mid A_t = 0, I_t = 1, H_t] \times \mid I_t = 1, S_{kt}\big] = f_{kt}(S_{kt})^{\mathrm{T}}\beta_k, \tag{7}$$

where, as before, $f_{kt}(S_{kt})$ is a vector of features involving S_{kt} and time t. To form the estimating function for β_k, we replace W_t in (6) by the product $I_t W_t$. The working model and the treatment probability models are conditional on $I_t = 1$. A more general version of the resulting estimating equation is provided in display (12) of Supplement C. Proofs can be found in Supplement C.

5. Implementation

The weighting and centering estimation method can be implemented using standard software for GEEs, provided that we: (i) incorporate $I_t W_t$ as "prior weights" and (ii) employ an independence working correlation matrix. The standard errors provided in Proposition 1 directly correspond to the sandwich variance-covariance estimator provided by GEE software. From existing work on GEEs, it is well understood that the sandwich estimator is nonconservative in small samples. To address this, whenever $n \le 50$, we apply (Mancl and DeRouen 2001) small sample correction to the term $\mathbb{P}_n U_W(\hat{\alpha}_k, \hat{\beta}_k)^{\otimes 2}$ in the estimator of the variance; in particular we premultiply the $(T - k + 1) \times 1$ vector of each person's residuals in U_W by the inverse of the identity matrix minus the leverage for this person. Also, as in Liao et al. (2015), we use critical values from a t distribution or a Hotelling's T-squared distribution. In particular, if we wish to test the null hypothesis for a linear combination of β_k—for example, test $c^{\mathrm{T}}\beta_k = 0$ for a known p-dimensional vector c—then we use the critical value $t^{-1}_{n-p-q}(1 - \alpha_0)$ where, p is the dimension of β_k, q is the dimension of α_k, and α_0 is the significance level. More generally, if we wish to conduct a p'-dimensional multivariate test of β_k—for example, test $z^{\mathrm{T}}\beta_k = 0$ for a known $p \times p'$ matrix z—then the critical value is $F^{-1}_{p',n-q-p}(\frac{(n-q-p')(1-\alpha_0)}{p'(n-q-1)})$.

When either $\tilde{p}_t(1 \mid S_{kt})$ or $p_t(1 \mid H_t)$ is estimated, the sandwich variance-covariance estimator must be adjusted to account for the additional sampling error (see Supplement C). See Supplement E to obtain code that calculates standard errors using R (R Core Team 2015).

6. Simulation Study

Here, we evaluate the proposed centering and weighting method via simulation experiments.

The following, simple, generative model will allow us to illustrate the proposed method and compare it with existing methods. Consider data arising from an MRT (so the randomization probability $p_t(1|H_t)$ is known). The generative model for the response, Y_{t+1}, is a linear model in $(A_t, S_t, A_{t-1}, S_{t-1}, A_{t-2}, A_tS_t, A_{t-1}S_t, A_{t-2}S_{t-1})$, for $S_t \in \{-1, 1\}$. For convenience in reading off the marginal effects, we write this model as $Y_{t+1} = \theta_1(S_t - E[S_t \mid A_{t-1}, H_{t-1}]) + \theta_2(A_{t-1} - p_{t-1}(1 \mid H_{t-1})) + (A_t - p_t(1 \mid H_t))(\beta^*_{10} + \beta^*_{11}S_t) + \epsilon_{t+1}$. Here, the randomization probability is given by $p_t(1 \mid H_t) = \mathrm{expit}(\eta_1 A_{t-1} + \eta_2 S_t)$, $\Pr(S_t = 1 \mid A_{t-1}, H_{t-1}) = \mathrm{expit}(\xi A_{t-1})$ (note $A_0 = 0$), and $\epsilon_t \sim N(0, 1)$ with $\mathrm{Corr}(\epsilon_u, \epsilon_t) = 0.5^{|u-t|/2}$. Throughout, for simplicity, each subject is available at every treatment occasion: $I_t = 1$ ($t = 1, \ldots, T$). In the simulation scenarios below, we fix $\theta_1 = 0.8$ and $\beta^*_{10} = -0.2$ and we vary $(\theta_2, \beta^*_{11}, \eta_1, \eta_2, \xi)$.

The marginal proximal (lag $k = 1$) effect is given by $E[E[Y_{t+1} \mid A_t = 1, H_t] - E[Y_{t+1} \mid A_t = 0, H_t]] = \beta^*_{10} + \beta^*_{11} E[S_t]$. Note that if $\beta^*_{11} = 0$ or $E[S_t] = 0$ (i.e., by setting $\xi = 0$), then the marginal proximal treatment effect is constant in time and is given by $\beta^*_1 = \beta^*_{10} = -0.2$. Throughout, for simplicity, we consider scenarios with $\beta^*_{11} = 0$ or $\xi = 0$; however, as discussed in Section 3, the method does not require treatment effects that are constant in time.

Here, we consider three simulation experiments. All three simulation experiments concern estimation of the marginal proximal treatment effect β^*_1. Thus in all cases when the weighted and centered method is used, $f_{1t}(S_{1t}) = (1)$ in the estimating function (6) (i.e., $S_{1t} = \emptyset$). We report average $\hat{\beta}_1$

Table 1. Comparison of three estimators of the marginal proximal treatment effect, $\hat{\beta}_1$, when an important moderator is omitted.

	Weighted and centered				GEE-IND				GEE-AR(1)			
β_{11}^*	Mean	SD	RMSE	CP	Mean	SD	RMSE	CP	Mean	SD	RMSE	CP
0.2	− 0.20	0.08	0.08	0.96	**− 0.17**	0.07	0.07	0.94	**− 0.16**	0.04	0.06	**0.86**
0.5	− 0.20	0.08	0.08	0.95	**− 0.14**	0.07	0.09	**0.88**	**− 0.13**	0.05	0.09	**0.70**
0.8	− 0.20	0.08	0.08	0.95	**− 0.10**	0.07	0.12	**0.78**	**− 0.10**	0.05	0.12	**0.57**

NOTES: RMSE, root mean squared error and SD, standard deviation of $\hat{\beta}_1$; CP, 95% confidence interval coverage probability for $\beta_1^* = -0.2$. Results are based on 1000 replicates with $n = T = 30$. Boldface indicates whether Mean or CP are significantly different, at the 5% level, from −0.2 or 0.95, respectively. GEE-IND is the same as the proposed method but with $W_t = 1$ and no centering. In GEE-AR(1) includes an AR(1) working correlation matrix.

point estimates, standard deviation, and root mean squared error of $\hat{\beta}_1$, and 95% confidence interval coverage probabilities for $n = T = 30$ across 1000 replicates. Confidence intervals are based on standard errors that are corrected for the estimation of weights and/or small samples (see Section 5). The tables below omit the average estimated standard errors; these are provided in Supplement D and closely correspond to the standard deviations of the point estimates. Supplement D also reports additional results for $n = 30, 60$ with $T = 30, 50$ (results were similar for different T values), and compares the proposed method versus centering but not weighting ($W_t = 1$ for all t) in a fourth simulation experiment.

The first simulation experiment concerns the estimation of β_1^* when an important moderator exists. This experiment illustrates that, when primary interest is in the marginal proximal treatment effect, weighting and centering is preferable over GEE. In the data generative model, we set $\theta_2 = 0$, $\eta_1 = -0.8$, $\eta_2 = 0.8$, and $\xi = 0$ (recall $\xi = 0$ implies that the true marginal proximal treatment effect is $\beta_1^* = -0.2$). Different scenarios were devised by setting β_{11}^* to one of 0.2, 0.5, 0.8, giving, respectively, a small, medium, or large degree of moderation by S_t. Since η_1 and η_2 are nonzero, the treatment A_t is assigned with a probability depending on both S_t and past treatment A_{t-1}, for each t.

In the weighted and centered analysis, we parameterize and estimate $\tilde{p}_t$. In particular, $\tilde{p}_t(a; \hat{\rho}) = \hat{\rho}^a(1-\hat{\rho})^{1-a}$ where $\hat{\rho} = \mathbb{P}_n \sum_{t=1}^T A_t/T$. The weights are set to $W_t = \hat{\rho}^{A_t}(1-\hat{\rho})^{1-A_t}/p_t(A_t \mid H_t)$ and the working model for $E[W_tY_{t+1} \mid H_t]$ is $\alpha_{10} + \alpha_{11}S_t$ (i.e., $g_{1t}(H_t) = (1, S_t)^T$). Thus, the estimating function in (6) is given by

$$\sum_{t=1}^T (Y_{t+1} - (\alpha_{10} + \alpha_{11}S_t) - (A_t - \hat{\rho})\beta_1) W_t \begin{pmatrix} (1, S_t)^T \\ A_t - \hat{\rho} \end{pmatrix}.$$

A common alternative would be a GEE analysis with an independence working correlation matrix. The GEE estimating function with an independence working correlation matrix (GEE-IND) is the above estimating function but with $W_t = 1$ for all t and A_t not centered. A more likely alternate that would be used in the mobile health literature is a GEE with an non-independence working correlation matrix (Schafer 2006); the resulting conditional mean model is the same as when random effects are used (Schwartz and Stone 2007; Bolger and Laurenceau 2013). We also provide a comparison with this alternative, using an AR(1) correlation matrix (GEE-AR(1)). Note that, to guarantee consistency in a GEE analysis, one would assume that the analysis model is correct; since here the analysis model is $Y_{t+1} \sim \alpha_{10} + \alpha_{11}S_t + A_t\beta_1$, the corresponding assumption would be that $E[Y_{t+1} \mid S_t, A_t] = \alpha_{10} + \alpha_{11}S_t + A_t\beta_1$ for some $(\alpha_{10}, \alpha_{11}, \beta_1)$. This assumption is false (no A_tS_t term). The weighting and centering method, on the other hand, does not require a model for the conditional mean. For consistency, the weighting and centering method only uses the assumption that $E[E[Y_{t+1} \mid S_t, A_t = 1] - E[Y_{t+1} \mid S_t, A_t = 0]] = \beta_1$ for some β_1.

Since the treatment effect term does not include S_t, the GEE conditional mean models are misspecified. Furthermore since $\eta_2 = 0.8$, the randomization probability $p_t(1 \mid H_t)$ depends on the underlying moderator S_t. We therefore anticipate the $\hat{\beta}_1$ from the GEE methods to be a biased estimator of the marginal treatment effect of $\beta_1^* = -0.2$ and we expect this bias to increase proportional to β_{11}^*. On the other hand, all of the requirements needed to achieve consistency in the proposed method are satisfied; hence, the $\hat{\beta}_1$ from the weighted and centered method should be unbiased, regardless of the value for β_{11}^*. These conjectures concerning bias are supported by Table 1. In addition, (i) for $\beta_{11}^* = 0.5, 0.8$ the RMSE for GEE is greater than or equal to the RMSE for the proposed method; and (ii) for all β_{11}^* the proposed method achieves nominal 95% coverage, whereas, the GEE methods generally do not (an exception was for $\beta_{11}^* = 0.2$ with GEE-IND). For further results see Table 5 in the Supplement.

The second and third simulation experiments focus on the proposed weighted and centered estimator. The second experiment illustrates that the ability to stabilize the weights is limited, since weighted least squares is prone to bias if the numerator of W_t depends on variables that are not in S_{kt}. In the data generative model, we set $\theta_2 = -0.1$, $\beta_{11}^* = 0.5$, $\eta_1 = -0.8$, $\eta_2 = 0.8$, and $\xi = 0$. Thus as above, the randomization probability for A_t depends on both S_t and past treatment A_{t-1} ($t = 1, \ldots, T = 100$). Here, since $\beta_{11}^* = 0.5$, S_t is a moderator of the proximal effect of treatment and since $\theta_2 = \beta_1^*/2 = -0.1$ there is a lag $k = 2$ treatment effect of A_{t-1} on Y_{t+1}.

In the data analysis using (6), the weighted and centered method, the working model for $E[W_tY_{t+1} \mid H_t]$ is again $\alpha_{10} + \alpha_{11}S_t$; thus, $g_{1t}(H_t) = (1, S_t)$. As before we assume $E[E[Y_{t+1} \mid S_t, A_t = 1] - E[Y_{t+1} \mid S_t, A_t = 0]] = \beta_1$ for some β_1 thus $f_{1t}(S_{1t}) = 1$. The denominator of the weight W_t is the known randomization probability, $p_t(A_t \mid H_t)$. We consider two different choices for $\tilde{p}_t$ (hence, two different choices for centering A_t and for the numerator of W_t): (i) A choice that is constant in t. Here, $\tilde{p}_t(a; \hat{\rho}) = \hat{\rho}^a(1-\hat{\rho})^{1-a}$ where $\tilde{p}_t(1; \hat{\rho}) = \hat{\rho} = \mathbb{P}_n \sum_{t=1}^T A_t/T$. The weights are $W_t(A_t, H_t) = \hat{\rho}^{A_t}(1-\hat{\rho})^{1-A_t}/p_t(A_t \mid H_t)$; (ii) A choice that depends on S_t. Here, instead, $\tilde{p}_t(1 \mid S_t; \hat{\rho}) = \text{expit}(\hat{\rho}_0 + \hat{\rho}_1 S_t)$,

Table 2. Weighted and centered estimator of the marginal proximal treatment effect, $\hat{\beta}_1$, using two choices for $\tilde{p}_t$.

$\tilde{p}_t$	Mean	SD	RMSE	CP
Constant in t (i)	− 0.20	0.08	0.08	0.94
Depends on S_t (ii)	**− 0.14**	0.09	0.11	**0.89**

NOTES: RMSE, root mean squared error and SD, standard deviation of $\hat{\beta}_1$; CP, 95% confidence interval coverage probability for $\beta_1^* = -0.2$. Results are based on 1000 replicates with $n = T = 30$. Boldface indicates whether Mean or CP are significantly different, at the 5% level, from −0.2 or 0.95, respectively.

where $\hat{\rho} = (\hat{\rho}_0, \hat{\rho}_1)$ is the solution to $\mathbb{P}_n \sum_t \exp(\rho_0 + \rho_1 S_t)\{\text{expit}(\rho_0 + \rho_1 S_t)(1 - \text{expit}(\rho_0 + \rho_1 S_t))\}^{-1}(A_t - \text{expit}(\rho_0 + \rho_1 S_t))(1, S_t)^{\mathrm{T}} = 0$. In (i) the probability in the numerator is constant for all W_t $(t = 1, \dots, T = 30)$. In (ii), the probability in the numerator depends on S_t yet interest is in a marginal proximal effect β_1 (S_t is not a part of $f_{1t}(S_{1t})$). Hence, we anticipate bias in $\hat{\beta}_1$ under (ii), but not (i). This is indeed reflected in Table 2, with (ii) exhibiting bias and achieving a coverage probability of 89%. For further results see Table 5 in Supplement.

The third simulation experiment illustrates that employing a nonindependence working correlation structure with the weighted and centered method can result in bias. In the data generative model, we set $\theta_2 = -0.1$, $\beta_{11}^* = 0$, $\eta_1 = \eta_2 = 0$, and $\xi = 0.1$. There is no moderation of the proximal effect, since $\beta_{11}^* = 0$. Unlike the above scenarios, here the predictor S_t is influenced by A_{t-1} (since $\xi = 0.1$), and because $\theta_2 = \beta_1^*/2 = -0.1$, there is a lag $k = 2$ treatment effect of A_{t-1} on Y_{t+1}. Treatment is randomized with fixed probability $p_t(1 \mid H_t) = 0.5$ for each $t = 1, \dots, T = 30$ since $\eta_1 = \eta_2 = 0$.

In the data analysis using (6), the weighted and centered method, the working model for $\mathrm{E}[W_t Y_{t+1} \mid H_t]$ is again $\alpha_{10} + \alpha_{11} S_t$; thus, $g_{1t}(H_t) = (1, S_t)$. In both data analyses, we correctly model $\mathrm{E}[\mathrm{E}[Y_{t+1} \mid S_t, A_t = 1] - \mathrm{E}[Y_{t+1} \mid S_t, A_t = 0]]$ by a constant, here denoted by β_1 thus $f_{1t}(S_{1t}) = 1$. We set $\tilde{p}_t(1) = 0.5$ thus the weights are $W_t = 1$ for all $t = 1, \dots, T = 30$. We compare the use of (i) the estimating function in (6), which corresponds to an independent working correlation structure, versus (ii) using a working AR(1) correlation matrix assuming a correlation of $0.5^{|u-t|/2}$ between times u and t. In the latter case, the estimating function is

$$\sum_{t=1}^{T} \begin{pmatrix} (1, S_t)^{\mathrm{T}} \\ A_t - 0.5 \end{pmatrix} \sum_{u=1}^{T} \upsilon_{tu}(Y_{u+1} - (\alpha_{10} + \alpha_{11} S_u) - (A_u - 0.5)\beta_1),$$

where υ_{tu} is the (t, u) entry of V^{-1}, where the (t, u) entry in V is $0.5^{|u-t|/2}$. While AR(1) might better represent the true correlation matrix than an independence correlation matrix, we expect (ii) to induce bias as this marginal model includes time-varying covariates. Table 3 demonstrates this result, with (ii) exhibiting bias and achieving a coverage probability of 65%. Further results are provided in Table 7 in the Supplement.

7. Application

BASICS-Mobile is a pilot study, with $n = 28$, $T = 28$. The response Y_{t+1} is the smoking rate from the tth occasion to the next self-report, and participants are presumed available only if they completed the preceding self-report. So the availability I_t is the self-report completion status just prior to t and the treatment decision D_t is 1 only if a mindfulness message is provided at t. Otherwise, $D_t = 0$.

Table 3. Weighted and centered estimator of the proximal effect, $\hat{\beta}_1$, with different working correlation structures.

Working correlation	Mean	SD	RMSE	CP
Independent (i)	− 0.20	0.07	0.07	0.96
AR(1) (ii)	**− 0.13**	0.06	0.09	**0.66**

NOTES: RMSE, root mean squared error and SD, standard deviation of $\hat{\beta}_1$; CP, 95% confidence interval coverage probability for $\beta_1^* = -0.2$. Results are based on 1000 replicates with $n = T = 30$. Boldface indicates whether Mean or CP are significantly different, at the 5% level, from −0.2 or 0.95, respectively.

BASICS-Mobile was neither a sequentially randomized trial nor an observational study. Treatment delivery at occasion t was based on a complex decision rule involving primarily a self-reported measure that the user had an urge or inclination to smoke at the preceding self-report (urge_t), an indicator for the first three treatment occasions ($1(t < 4)$), and a combination of other variables. For illustrative purposes, we provide an analysis acting as though the study was observational and assuming sequential ignorability; we estimate (with logistic regression) the treatment probabilities in the denominator of the weights, $p_t(1 \mid H_t)$, based on $(Y_t, \text{urge}_t, 1(\mathrm{t} < 4))$ using

$$p_t(1 \mid H_t; \hat{\eta}) = \text{expit}(0.69 + 0.02 Y_t + 0.17 \text{urge}_t - 0.28\, 1(t < 4) + 0.70 \text{urge}_t\, 1(\mathrm{t} < 4)).$$

We examine proximal ($k = 1$) and lag-2 ($k = 2$) treatment effects. For the proximal effect analysis, we examine one candidate time-varying moderator $S_{1t} = \text{incr}_t$, which indicates whether or not the user reported an increase in need to self-regulate thoughts over the two self-reports preceding t. Thus in the estimating function (6) for the proximal effect analysis, we set $f_{1t}(S_{1t}) = (1, \text{incr}_t)^{\mathrm{T}}$. For the delayed effect analysis, we consider only the marginal lag-2 effect; thus, $f_{2t}(S_{2t}) = (1)$ in the estimating function (6). For both analyses, we centered and estimated the numerator of the weights based on $\tilde{p}_t(a; \hat{\rho}) = \hat{\rho}^a(1 - \hat{\rho})^{1-a}$ where $\hat{\rho} = \mathbb{P}_n \sum_{t=1}^T I_t A_t / \mathbb{P}_n \sum_{t=1}^T I_t = 0.67$. Hence, for both analyses, the weights were set to $W_t = \hat{\rho}^{A_t}(1 - \hat{\rho})^{1-A_t}/p_t(A_t \mid H_t; \hat{\eta})$. In the working model for both analyses, a variety of predictors are incorporated in $g_{kt}(H_t)$ $(k = 1, 2)$, including an intercept term, incr_t, current urge to smoke, Y_{t+1-k}, time of day, the interaction between Y_{t+1-k} and time of day, baseline smoking severity, baseline drinking level, age, and gender.

The data analysis leads to several conclusions. First, the mindfulness message achieved a reduction in the average next-reported smoking rate, but only when the user was experiencing either a stable or decreased need to self-regulate (95% CI −5.45 to −0.15 cigarettes per day; see Table 4). Otherwise no

Table 4. Proximal and lag-2 treatment effects estimated from BASICS-Mobile data.

Treatment effect	Estimate	SE	95% CI	p-Value
Proximal, increase in need to self-regulate	− 0.06	0.95	(−1.27, 1.16)	0.99
Proximal, no increase in need to self-regulate	− 2.80	1.29	(−5.45,−0.15)	0.04
Delayed	− 0.49	0.61	(−1.74,0.76)	0.43

proximal treatment effect is apparent. Second, there is no evidence to support the presence of an overall lag-2 effect, with a 95% CI of -1.74 to 0.76 cigarettes per day for the average reduction achieved by mindfulness treatment at the second-to-last treatment occasion. Estimated standard errors (SEs) take into account sampling error in estimated treatment probabilities (see (13) for the formula), and are corrected for small n (see Section 5 for details on the correction).

8. Discussion

In this article, we define treatment effects suited for mobile interventions that enable frequent measurements and frequent delivery of treatments. As we discussed, the effect definition as provided in (1) and (2) is atypical in the field of causal inference in that the underlying mechanism for the assigned treatment is part of the definition of the causal effect. However, this definition of the causal effects is consistent with the effects defined via most models for intensively collected longitudinal data (see Schafer 2006; Schwartz and Stone 2007; and, more recently, Bolger and Laurenceau 2013). Commonly the model for the conditional mean of a time-varying response given time-varying covariates is a linear model (possibly with the use of covariates defined by flexible basis functions). If treatment indicators as well as interactions between the treatment indicators and time varying covariates are included in the linear model, then the meaning of coefficients of these covariates coincide with the moderated proximal effect defined here. However, estimation of these casual coefficients using most common approaches (Schafer 2006; Schwartz and Stone 2007; Bolger and Laurenceau 2013), that is, either GEE approaches or approaches that employ random effects, can cause bias. Indeed the large sample and simulation results provided here show that straightforward use of GEEs (without weighting) is not guaranteed to consistently estimate β_k^*.

Since the conditional mean functions for models with random intercepts or random coefficients (e.g., Goldstein 2011) are the same as those in GEEs, we expect that likelihood-based methods that use the induced correlation structure in the estimation will generally be biased. This connection is important given the fact that, in the analysis of intensive longitudinal data, there is a preference for including random effects and, when GEE models are used, to use a nonindependence working correlation structure (such as exchangeable, $\mathrm{Corr}(Y_u, Y_t) = r$ $(u \neq t)$, or AR(1), $\mathrm{Corr}(Y_u, Y_t) = r^{|u-t|}$) to improve precision (Schafer 2006, p. 58). Indeed the large sample and simulation results provided here show that GEEs based on a nonindependence working covariance structure is not guaranteed to consistently estimate β_k^*. Future work is needed on whether or how to incorporate random effects in the estimation of proximal and lagged treatment effects.

There are a number of other directions for future work. First, throughout we limited attention to a continuous response and binary treatment decisions. The extension to the multi-category treatment setting (e.g., $A_t \in \{1, 2, \dots, C\}$) is relatively straightforward involving, for example, the selection of a referent category (say, category C) and the use of $C - 1$ centered terms of the form $1(A_t = c) - \tilde{p}_t(c|S_{kt})$ where $c \in \{1, 2, \dots, C - 1\}$. Note that here scientists might select different candidate moderators depending on the contrast. Second, an extension to the binary response setting is more difficult, potentially requiring an extension of the multiplicative or log-linear structural nested mean model (Vansteelandt and Joffe 2014). Such an extension will be nontrivial if one wants to preserve the ability to estimate treatment effects that are only conditional on S_{1t} as opposed to all of the past, H_t. Third, lagged effects $(k > 1)$ were defined similar to proximal effects $(k = 1)$, but in future work one might rather be interested in a lagged effect that quantifies the accumulation of past treatment. Fourth, since small to moderate treatment effects may be difficult to detect, yet potential response predictors that can be used in the working models to reduce error variance are numerous, future work could consider penalized methods for the working model to accommodate and select from the large number of predictors. Fifth, although the primary motivation for this article is to estimate proximal or lagged effects using data arising from micro-randomized trials (Klasnja et al. 2015; Liao et al. 2015; Dempsey et al. 2015), work on how to best generalize and combine the methods here with the current research in causal inference for observational studies is needed. Finally, here we considered analyses that are similar to longitudinal analyses; however, interesting alternative approaches might have more of a "system dynamics" flavor and employ time-series modeling or Markovian process modeling.

Funding

Funding was provided by the National Institute on Drug Abuse (P50DA039838, R01DA039901, R01DA015697), National Institute on Alcohol Abuse and Alcoholism (R01AA023187), National Heart Lung and Blood Institute (R01HL125440), and National Institute of Biomedical Imaging and Bioengineering (U54EB020404).

References

Bolger, N., and Laurenceau, J.-P. (2013), *Intensive Longitudinal Methods: An Introduction to Diary and Experience*, New York, NY: The Guilford Press. [1112,1118,1120]

Bowen, S., and Marlatt, A. (2009), "Surfing the Urge: Brief Mindfulness-Based Intervention for College Student Smokers," *Psychology of Addictive Behaviors*, 23, 666–671. [1113]

Brumback, B., Greenland, S., Redman, M., Kiviat, N., and Diehr, P. (2003), "The Intensity-Score Approach to Adjusting for Confounding," *Biometrics*, 59, 274–285. [1116]

Dempsey, W., Liao, P., Klasnja, P., Nahum-Shani, I., and Murphy, S. A. (2015), "Randomized Trials for the Fitbit Generation," *Significance*, 12, 20–23. [1112,1120]

Dimeff, L. A. (1999), *Brief Alcohol Screening and Intervention for College Students (BASICS): A Harm Reduction Approach*, New York: Guilford Press. [1113]

Free, C., Phillips, G., Galli, L., Watson, L., Felix, L., Edwards, P., Patel, V., and Haines, A. (2013), "The Effectiveness of Mobile-Health Technology-Based Health Behaviour Change or Disease Management Interventions for Health Care Consumers: A Systematic Review," *PLoS Medicine*, 10, e1001362. [1112]

Goetgeluk, S., and Vansteelandt, S. (2008), "Conditional Generalized Estimating Equations for the Analysis of Clustered and Longitudinal Data," *Biometrics*, 64, 772–780. [1116]

Goldstein, H. (2011), *Multilevel Statistical Models*, New York: Wiley. [1120]

Heron, K. E., and Smyth, J. M. (2010), "Ecological Momentary Interventions: Incorporating Mobile Technology into Psychosocial and Health Behaviour Treatments," *British Journal of Health Psychology*, 15, 1–39. [1113]

Hong, G., and Raudenbush, S. W. (2006), "Evaluating Kindergarten Retention Policy," *Journal of the American Statistical Association*, 101, 901–910. [1114]

Klasnja, P., Hekler, E., Shiffman, S., Boruvka, A., Almirall, D., Tewari, A., and Murphy, S. (2015), "Micro-Randomized Trials: An Experimental Design for Developing Just-In-Time Adaptive Interventions," *Health Psychology*, 34, 1220–1228. [1112,1116,1120]

Liang, K.-Y., and Zeger, S. L. (1986), "Longitudinal Data Analysis Using Generalized Linear Models," *Biometrika*, 73, 13–22. [1116]

Liao, P., Klasnja, P., Tewari, A., and Murphy, S. A. (2015), "Sample Size Calculations for Micro-Randomized Trials in mHealth," *Statistics in Medicine*, 35, 1944–1971. [1112,1116,1117,1120]

Mancl, L. A., and DeRouen, T. A. (2001), "A Covariance Estimator for GEE with Improved Small-Sample Properties," *Biometrics*, 57, 126–134. [1117]

Mancl, L. A., and Leroux, B. G. (1996), "Efficiency of Regression Estimates for Clustered Data," *Biometrics*, 52, 500–511. [1116]

Mohr, D. C., Schueller, S. M., Montague, E., Burns, M. N., and Rashidi, P. (2014), "The Behavioral Intervention Technology Model: An Integrated Conceptual and Technological Framework for eHealth and mHealth Interventions," *Journal of Medical Internet Research*, 16, e146. [1112]

Muessig, K. E., Pike, E. C., Legrand, S., and Hightow-Weidman, L. B. (2013), "Mobile Phone Applications for the Care and Prevention of HIV and Other Sexually Transmitted Diseases: A Review," *Journal of Medical Internet Research*, 15, e1. [1112]

Neyman, J. (1990), "On the Application of Probability Theory to Agricultural Experiments: Essay on Principles," *Statistical Science*, 5, 465–472. [1113]

Pepe, M. S., and Anderson, G. L. (1994), "A Cautionary Note on Inference for Marginal Regression Models with Longitudinal Data and General Correlated Response Data," *Communications in Statistics—Simulation and Computation*, 23, 939–951. [1116]

R Core Team (2015), *R: A Language and Environment for Statistical Computing*, Vienna, Austria: R Foundation for Statistical Computing. [1117]

Robins, J. M. (1989), "The Analysis of Randomized and Non-Randomized AIDS Treatment Trials Using a New Approach to Causal Inference in Longitudinal Studies," in *Health Services Research Methodology: A Focus on AIDS*, eds. L. Sechrest, H. Freeman, A. Mulley, and National Center for Health Services Research and Health Care Technology Assessment, Rockville, MD: National Center for Health Services Research and Health Care Technology Assessment, Public Health Service, U.S. Department of Health and Human Services, pp. 113–159. [1112,1113]

——— (1994), "Correcting for Non-Compliance in Randomized Trials Using Structural Nested Mean Models," *Communications in Statistics—Theory and Methods*, 23, 2379–2412. [1112,1114,1115]

——— (1997), "Causal Inference from Complex Longitudinal Data," in *Latent Variable Modeling and Applications to Causality* (*Lecture Notes in Statistics*, Vol. 120), ed. M. Berkane, New York: Springer, pp. 69–117 [1112,1114,1115]

——— (1998), "Marginal Structural Models," in *1997 Proceedings of the Section on Bayesian Statistical Science*, Alexandria, VA: American Statistical Association, pp. 1–10. [1116]

——— (2004), "Optimal Structural Nested Models for Optimal Sequential Decisions," in *Proceedings of the Second Seattle Symposium in Biostatistics* (*Lecture Notes in Statistics*, Vol. 179), eds. D. Y. Lin, and P. J. Heagerty, New York: Springer, pp. 189–326 [1117]

Robins, J. M., Hernán, M. A., and Brumback, B. (2000), "Marginal Structural Models and Causal Inference in Epidemiology," *Epidemiology*, 11, 550–560. [1116]

Rubin, D. B. (1974), "Estimating Causal Effects of Treatments in Randomized and Nonrandomized Studies," *Journal of Educational Psychology*, 66, 688–701. [1113]

Schafer, J. L. (2006), "Marginal Modeling of Intensive Longitudinal Data by Generalized Estimating Equations," in *Models for Intensive Longitudinal Data*, eds. T. A. Walls, and J. L. Schafer, New York: Oxford University Press, pp. 38–62. [1112,1118,1120]

Schwartz, J. E., and Stone, A. A. (2007), "The Analysis of Real-Time Momentary Data: A Practical Guide," in *The Science of Real-Time Data Capture*, eds. A. A. Stone, S. Shiffman, A. A. Atienza, and L. Nebeling, New York: Oxford University Press, pp. 76–113. [1112,1118,1120]

Spring, B., Gotsis, M., Paiva, A., and Spruijt-Metz, D. (2013), "Healthy Apps: Mobile Devices for Continuous Monitoring and Intervention," *IEEE Pulse*, 4, 34–40. [1112]

Tchetgen Tchetgen, E. J., Glymour, M. M., Weuve, J., and Robins, J. (2012), "Specifying the Correlation Structure in Inverse-Probability-Weighting Estimation for Repeated Measures," *Epidemiology*, 23, 644–646. [1116]

Vanderweele, T. J., Hong, G., Jones, S. M., and Brown, J. L. (2013), "Mediation and Spillover Effects in Group-Randomized Trials: A Case Study of the 4Rs Educational Intervention," *Journal of the American Statistical Association*, 108, 469–482. [1114]

Vansteelandt, S. (2007), "On Confounding, Prediction and Efficiency in the Analysis of Longitudinal and Cross-sectional Clustered Data," *Scandinavian Journal of Statistics*, 34, 478–498. [1116]

Vansteelandt, S., and Joffe, M. (2014), "Structural Nested Models and g-Estimation: The Partially Realized Promise," *Statistical Science*, 29, 707–731. [1120]

Wang, L., Rotnitzky, A., Lin, X., Millikan, R. E., and Thall, P. F. (2012), "Evaluation of Viable Dynamic Treatment Regimes in a Sequentially Randomized Trial of Advanced Prostate Cancer," *Journal of the American Statistical Association*, 107, 493–508. [1117]

Witkiewitz, K., Desai, S. A., Bowen, S., Leigh, B. C., Kirouac, M., and Larimer, M. E. (2014), "Development and Evaluation of a Mobile Intervention for Heavy Drinking and Smoking Among College Students," *Psychology of Addictive Behaviors*, 28, 639–650. [1112,1113]
