# Supplementary material for: Penalized G-estimation for effect modifier selection in a structural nested mean model for repeated outcomes
Source: Biometrics. Author manuscript; Available in PMC 2026 Jun 16. (PMC13271025; doi:10.1093/biomtc/ujae165)
Supplement: Codes [file NIHMS2179184-supplement-Codes.zip › codes_BIOM2024119M/Reproduce simulation tables/Simulations/Boruvka_2018/uasa_a_1305274_sm1867.pdf]

Supplementary Material

A Lagged Treatment Effects

A.1 Connection to Treatment Blips in the Structural Nested Mean Model

This Supplement connects a generalization of the structural nested mean model (SNMM; Robins 1989, 1994) to the lag k treatment effect defined in Section 2.3. In particular, consider a causal effect or treatment "blip" as defined by the SNMM framework (Robins 1994, Section 3a), with a minor departure in the choice of the reference treatment regime. We show how these effects are additive on the conditional mean of the potential proximal response. We conclude by connecting this particular SNMM generalization to the lag k moderated effect (2) considered throughout the paper.

The typical reference treatment regime used to define the treatment "blip" functions under the SNMM framework, is a prespecified non-random reference regime; here instead our reference treatment regime is stochastic and will match the conditional distribution of the treatments given history in the data generating distribution. In particular suppose that in the data generating distribution $\Pr(A_t = 1 \mid \bar{X}_t = \bar{x}_t, \bar{Y}_t = \bar{y}_t, \bar{A}_{t-1} = \bar{a}_{t-1}] = p_t(1 \mid h_t)$ for each t and where $h_t = (\bar{x}_t, \bar{y}_t, \bar{a}_{t-1})$. Then the reference treatment regime for the potential treatment is given, for each t, by $\Pr(A_t(\bar{a}_{t-1}) = 1 \mid H_t(\bar{a}_{t-1}) = h_t] = p_t(1 \mid h_t)$ (recall $H_t(\bar{a}_{t-1}) = (\bar{X}_t(\bar{a}_{t-1}), \bar{Y}_t(\bar{a}_{t-1}), \bar{A}_{t-1}(\bar{a}_{t-2})))$.

The treatment blip of fixed $a_t \in \{0, 1\}$ versus stochastic treatment $A_t(\bar{a}_{t-1})$ on the proximal response Y_{t+1} is

$$\mu_{t,t+1}(h_t, \bar{a}_t) = \mathrm{E}[Y_{t+1}(\bar{a}_t) - Y_{t+1}(\bar{a}_{t-1}, A_t(\bar{a}_{t-1})) \mid H_t(\bar{a}_{t-1}) = h_t].$$

The treatment blip of fixed $a_{t-1} \in \{0,1\}$ versus stochastic treatment $A_{t-1}(\bar{a}_{t-2})$ on the response Y_{t+1} is

$$\begin{aligned}
&\mu_{t-1,t+1}(h_{t-1}, \bar{a}_{t-1}) \\
&= \mathrm{E}[Y_{t+1}(\bar{a}_{t-1}, A_t(\bar{a}_{t-1})) - Y_{t+1}(\bar{a}_{t-2}, A_{t-1}(\bar{a}_{t-2}), A_t(\bar{a}_{t-2}, A_{t-1}(\bar{a}_{t-2}))) \mid H_{t-1}(\bar{a}_{t-2}) = h_{t-1}].
\end{aligned}$$

The treatment blip for general $u \leq t$ is defined similarly but with an increase in notation. However notice if we denote $A_2(A_1)$ by A_2 and so on with $A_t(\bar{A}_{t-1})$ denoted by A_t, and we denote $A_{u+1}(\bar{A}_{u-1}, a)$ by $A_{u+1}^{a_u=a}$, $A_{u+2}(\bar{A}_{u-1}, a, A_{u+1}^{a_u=a})$ by $A_{u+2}^{a_u=a}$ and so on with $A_t(\bar{A}_{u-1}, a, A_{u+1}^{a_u=a}, \ldots, A_{t-1}^{a_u=a})$ by $A_t^{a_u=a}$ then we have the compact form

$$\mu_{u,t+1}(H_u(\bar{A}_{u-1}), \bar{A}_{u-1}, a) = \mathrm{E}\big[Y_{t+1}(\bar{A}_{u-1}, a_u, A_{u+1}^{a_u=a}, \ldots, A_t^{a_u=a}) - Y_{t+1}(\bar{A}_t) \mid \bar{H}_u(\bar{A}_{u-1})\big]. \tag{8}$$

Assume consistency and sequential ignorability. Then

$$\begin{aligned}
&\mathrm{E}\big[Y_{t+1}(\bar{A}_{u-1}, a, A_{u+1}^{a_u}, \ldots, A_t^{a_u=a}) \,\big|\, H_u(\bar{A}_{u-1})\big] \\
=\ &\mathrm{E}\big[Y_{t+1}(\bar{A}_{u-1}, a, A_{u+1}^{a_u=a}, \ldots, A_t^{a_u=a}) \,\big|\, H_u(\bar{A}_{u-1}), A_u = a_u\big] \\
=\ &\mathrm{E}\big[Y_{t+1}(\bar{A}_{u-1}, A_u, A_{u+1}^{a_u=A_u}, \ldots, A_t^{a_u=A_u}) \,\big|\, H_u(\bar{A}_{u-1}), A_u = a_u\big] \\
=\ &\mathrm{E}\big[Y_{t+1}(\bar{A}_t) \mid H_u(\bar{A}_{u-1}), A_u = a_u\big]
\end{aligned}$$

where the first equality follows from the consistency and sequential ignorability assumptions (recall that $H_u = H_u(\bar{A}_{u-1})$) and the last two equalities follow by the definitions of $A_j^{a_u}$ and A_j. Thus the treatment blip satisfies

$$\mathrm{E}[\mu_{u,t+1}(H_u(\bar{A}_{u-1}), \bar{A}_u) \mid H_u(\bar{A}_{u-1})] = 0, \tag{9}$$

for each $u = 1, \ldots, t$ and $t = 1, \ldots, T$. The lag k treatment effect (2) can be expressed as the expected contrast of the treatment blips (8):

$$\begin{aligned}
&\mathrm{E}\big[\mu_{t,t+k}(H_t(\bar{A}_{t-1}), \bar{A}_{t-1}, 1) - \mu_{t,t+k}(H_t(\bar{A}_{t-1}), \bar{A}_{t-1}, 0) \mid S_{kt}(\bar{A}_{t-1})\big] \\
&= \mathrm{E}\big[Y_{t+k}(\bar{A}_{t-1}, 1, A_{t+1}^{a_u=1}, \ldots, A_{t+k-1}^{a_u=1}) - Y_{t+k}(\bar{A}_{t-1}, 0, A_{t+1}^{a_u=0}, \ldots, A_{t+k-1}^{a_u=0}) \,\big|\, S_{kt}(\bar{A}_{t-1})\big], \quad (10)
\end{aligned}$$

given the candidate moderators $S_{kt}(A_{t-1})$.

As in Robins (1989, 1994) the SNMM treatment blips are related to the conditional mean of $Y_{t+1}(\bar{a}_t)$ given $H_t(\bar{a}_{t-1})$ by way of a telescoping sum. For clarity we first provide the sum for $t = 3$.

$$\begin{aligned}
&\mathrm{E}[Y_4(\bar{a}_3) \mid H_3(\bar{a}_2) = h_3] \\
= \;&\mathrm{E}[Y_4(\bar{a}_3) - Y_4(\bar{a}_2, A_3(\bar{a}_2)) \mid H_3(\bar{a}_2) = h_3] \\
&+ \mathrm{E}[Y_4(\bar{a}_2, A_3(\bar{a}_2)) \mid H_3(\bar{a}_2) = h_3] - \mathrm{E}[Y_4(\bar{a}_2, A_3(\bar{a}_2)) \mid H_2(\bar{a}_1) = h_2] \\
&+ \mathrm{E}[Y_4(\bar{a}_2, A_3(\bar{a}_2)) - Y_4(a_1, A_2(a_1), A_3(a_1, A_2(a_1))) \mid H_2(\bar{a}_1) = h_2] \\
&+ \mathrm{E}[Y_4(a_1, A_2(a_1), A_3(a_1, A_2(a_1))) \mid H_2(\bar{a}_1) = h_2] - \mathrm{E}[Y_4(a_1, A_2(a_1), A_3(a_1, A_2(a_1))) \mid H_1 = h_1] \\
&+ \mathrm{E}\big[Y_4(a_1, A_2(a_1), A_3(a_1, A_2(a_1))) - Y_4(\bar{A}_3) \mid H_1 = h_1\big] \\
&+ \mathrm{E}\big[Y_4(\bar{A}_3) \mid H_1 = h_1\big] - \mathrm{E}\big[Y_4(\bar{A}_3)\big] \\
&+ \mathrm{E}\big[Y_4(\bar{A}_3)\big].
\end{aligned}$$

Denote $A_{u+1}(\bar{a}_{u-1}, A_u(\bar{a}_{u-1}))$ by $A_{u+1}^{\bar{a}_{u-1}}$, $A_{u+2}(\bar{a}_{u-1}, A_u(\bar{a}_{u-1}), A_{u+1}^{\bar{a}_{u-1}})$ by $A_{u+2}^{\bar{a}_{u-1}}$ and so on with $A_t(\bar{a}_{u-1}, A_u(\bar{a}_{u-1}), A_{u+1}^{a_{u-1}}, \ldots, A_{t-1}^{\bar{a}_{u-1}})$ by $A_t^{\bar{a}_{u-1}}$. Using this compact notation the treatment blips in (8) can be rewritten as

$$\mu_{u,t+1}(h_u, \bar{a}_u) = \mathrm{E}\big[Y_{t+1}(\bar{a}_u, A_{u+1}^{\bar{a}_u}, \ldots, A_t^{\bar{a}_u}) - Y_{t+1}(\bar{a}_{u-1}, A_u^{\bar{a}_{u-1}}, \ldots, A_t^{\bar{a}_{u-1}}) \mid \bar{H}_u(\bar{a}_{u-1}) = h_u\big].$$

The telescoping sum for general t using this compact notation is

$$
\begin{aligned}
&\mathrm{E}[Y_{t+1}(\bar{a}_t) \mid H_t(\bar{a}_{t-1}) = h_t] \\
= \ &\mathrm{E}\big[Y_{t+1}(\bar{a}_t) - Y_{t+1}(\bar{a}_{t-1}, A_t^{\bar{a}_{t-1}}) \mid H_t(\bar{a}_{t-1}) = h_t\big] \\
&+ \mathrm{E}[Y_{t+1}(\bar{a}_{t-1}, A_t^{\bar{a}_{t-1}}) \mid H_t(\bar{a}_{t-1}) = h_t] - \mathrm{E}[Y_{t+1}(\bar{a}_{t-1}, A_t^{\bar{a}_{t-1}}) \mid H_{t-1}(\bar{a}_{t-2}) = h_{t-1}] \\
&+ \mathrm{E}[Y_{t+1}(\bar{a}_{t-1}, A_t^{\bar{a}_{t-1}}) - Y_{t+1}(\bar{a}_{t-2}, A_{t-1}^{\bar{a}_{t-2}}, A_t^{\bar{a}_{t-2}}) \mid H_{t-1}(\bar{a}_{t-2}) = h_{t-1}] \\
&+ \mathrm{E}[Y_{t+1}(\bar{a}_{t-2}, A_{t-1}^{\bar{a}_{t-2}}, A_t^{\bar{a}_{t-2}}) \mid H_{t-1}(\bar{a}_{t-2}) = h_{t-1}] - \mathrm{E}[Y_{t+1}(\bar{a}_{t-2}, A_{t-1}^{\bar{a}_{t-2}}, A_t^{\bar{a}_{t-2}}) \mid H_{t-2}(\bar{a}_{t-3}) = h_{t-2}] \\
&\cdots \\
&+ \mathrm{E}[Y_{t+1}(a_1, A_2^{a_1}, \cdots, A_t^{a_1}) - Y_{t+1}(\bar{A}_t) \mid H_1 = h_1] \\
&+ \mathrm{E}[Y_{t+1}(\bar{A}_t) \mid H_1 = h_1] - \mathrm{E}[Y_{t+1}(\bar{A}_t)] \\
&+ \mathrm{E}[Y_{t+1}(\bar{A}_t)] \\
= \ &\mathrm{E}[Y_{t+1}(\bar{A}_t)] + \sum_{u=1}^{t} \mu_{u,t+1}(h_u, \bar{a}_u) + \sum_{u=1}^{t} \epsilon_{u,t+1}(h_u, \bar{a}_{u-1}),
\end{aligned}
\tag{11}
$$

where

$$
\begin{aligned}
\epsilon_{u,t+1}(h_u, \bar{a}_{u-1}) = \ &\mathrm{E}[Y_{t+1}(\bar{a}_{u-1}, A_u^{\bar{a}_{u-1}}, \ldots, A_t^{\bar{a}_{u-1}}) \mid H_u(\bar{a}_{u-1}) = h_u] \\
&- \mathrm{E}[Y_{t+1}(\bar{a}_{u-1}, A_u^{\bar{a}_{u-1}}, \ldots, A_t^{\bar{a}_{u-1}}) \mid H_{u-1}(\bar{a}_{u-2}) = h_{u-1}],
\end{aligned}
$$

are nuisance functions that satisfy the constraint $\mathrm{E}[\epsilon_{u,t+1}(H_u(\bar{a}_{u-1}), \bar{a}_{u-1}) \mid H_{u-1}(\bar{a}_{u-2})] = 0$, for each $\bar{a}_{u-1} \in \mathcal{A}_{u-1}$, $u = 1, \ldots, t$ and $t = 1, \ldots, T$.

A.2 Identification from Data

Here we derive the expression (3) of the lag k treatment effect (2). This is done under the consistency, positivity and sequential ignorability conditions described in Section 2.3.

To derive expression (3) for the lag k treatment effect (2), we show that

$$\mathrm{E}\big[Y_{t+k}(\bar{A}_{t-1}, a, A_{t+1}^{a_t=a}, \dots, A_{t+k-1}^{a_t=a}) \,\big|\, S_{kt}(\bar{A}_{t-1})\big] = \mathrm{E}\big[\mathrm{E}[Y_{t+k} \mid A_t = a, H_t] \,\big|\, S_{kt}\big]$$

and

$$\mathrm{E}\big[Y_{t+k}(\bar{A}_{t-1}, a, A_{t+1}^{a_t=a}, \dots, A_{t+k-1}^{a_t=a}) \mid S_{kt}(\bar{A}_{t-1})\big] = \mathrm{E}\left[\frac{1(A_t = a)}{p_t(a \mid H_t)} Y_{t+k} \,\middle|\, S_{kt}\right]$$

for $a \in \{0, 1\}$.

First recall that by consistency, $H_t = H_t(\bar{A}_{t-1})$ and $S_{kt} = S_{kt}(\bar{A}_{t-1})$. Second recall the definition of $A_{t+j}^{a_t=a}$, where in particular $A_{t+1}^{a_t=a}$ denotes $A_{t+1}(\bar{A}_{t-1}, a)$, $A_{t+2}^{a_t=a}$ denotes $A_{t+2}(\bar{A}_{t-1}, a, A_{t+1}^{a_t=a})$ and so on, with $A_{t+k-1}(\bar{A}_{t-1}, a, A_{t+1}^{a_t}, \dots, A_{t+k-2}^{a_t=a})$ denoted by $A_{t+k-1}^{a_t=a}$). So for each $j = 1, \dots, T - t + 1$, sequential ignorability implies that $A_{t+j}^{a_t=a}, a \in \{0, 1\}$ is independent of A_t given H_t. We have

$$\begin{aligned}
& \mathrm{E}[Y_{t+k}(\bar{A}_{t-1}, a, A_{t+1}^{a_t=a}, \dots, A_{t+k-1}^{a_t=a}) \mid S_{kt}(\bar{A}_{t-1})] \\
= \ & \mathrm{E}\big[\mathrm{E}\big[Y_{t+k}(\bar{A}_{t-1}, a, A_{t+1}^{a_t=a}, \dots, A_{t+k-1}^{a_t=a}) \mid H_t(\bar{A}_{t-1})\big] \,\big|\, S_{kt}(\bar{A}_{t-1})\big] \\
= \ & \mathrm{E}\big[\mathrm{E}\big[Y_{t+k}(\bar{A}_{t-1}, a, A_{t+1}^{a_t=a}, \dots, A_{t+k-1}^{a_t=a}) \mid H_t\big] \,\big|\, S_{kt}\big] \\
= \ & \mathrm{E}\big[\mathrm{E}\big[Y_{t+k}(\bar{A}_{t-1}, a, A_{t+1}^{a_t=a}, \dots, A_{t+k-1}^{a_t=a}) \mid H_t, A_t = a\big] \,\big|\, S_{kt}\big] \\
= \ & \mathrm{E}\big[\mathrm{E}\big[Y_{t+k}(\bar{A}_{t-1}, A_t, A_{t+1}^{a_t=A_t}, \dots, A_{t+k-1}^{a_t=A_t}) \mid H_t, A_t = a\big] \,\big|\, S_{kt}\big] \\
= \ & \mathrm{E}\big[\mathrm{E}\big[Y_{t+k}(\bar{A}_{t-1}, A_t, A_{t+1}, \dots, A_{t+k-1}) \mid H_t, A_t = a\big] \,\big|\, S_{kt}\big] \\
= \ & \mathrm{E}\big[\mathrm{E}[Y_{t+k} \mid H_t, A_t = a] \,\big|\, S_{kt}\big],
\end{aligned}$$

where the second equality holds by consistency, the third by sequential ignorability and the fifth follows from the definition of $A_{t+j}^{a_t=a}$ implying that $A_{t+j}^{a_t=A_t} = A_{t+j}$.

Next note that, by sequential ignorability, $\mathrm{E}[Y_{t+k}(\bar{A}_{t-1}, a, A_{t+1}^{a_t=a}, \dots, A_{t+k-1}^{a_t=a}) \mid H_t]\, \mathrm{E}[1(A_t =$

$a) \mid H_t]$ is equal to $\mathrm{E}[Y_{t+k}(\bar{A}_{t-1}, a, A_{t+1}^{a_t=a}, \ldots, A_{t+k-1}^{a_t=a})\, 1(A_t = a) \mid H_t]$. We have

$$
\begin{aligned}
&\mathrm{E}\big[Y_{t+k}(\bar{A}_{t-1}, a, A_{t+1}^{a_t=a}, \ldots, A_{t+k-1}^{a_t=a}) \bigm| S_{kt}(\bar{A}_{t-1})\big] \\
&= \mathrm{E}\Big[\mathrm{E}\big[Y_{t+k}(\bar{A}_{t-1}, a, A_{t+1}^{a_t=a}, \ldots, A_{t+k-1}^{a_t=a}) \bigm| H_t\big] \Bigm| S_{kt}\Big] \\
&= \mathrm{E}\left[\mathrm{E}\big[Y_{t+k}(\bar{A}_{t-1}, a, A_{t+1}^{a_t=a}, \ldots, A_{t+k-1}^{a_t=a}) \bigm| H_t\big] \frac{\mathrm{E}[1(A_t = a) \mid H_t]}{p_t(a \mid H_t)} \,\middle|\, S_{kt}\right] \\
&= \mathrm{E}\left[\mathrm{E}\left[Y_{t+k}(\bar{A}_{t-1}, a, A_{t+1}^{a_t=A_t}, \ldots, A_{t+k-1}^{a_t=A_t}) \frac{1(A_t = a)}{p_t(a \mid H_t)} \,\middle|\, H_t\right] \,\middle|\, S_{kt}\right] \\
&= \mathrm{E}\left[\mathrm{E}\left[Y_{t+k} \frac{1(A_t = a)}{p_t(a \mid H_t)} \,\middle|\, H_t\right] \,\middle|\, S_{kt}\right] \\
&= \mathrm{E}\left[Y_{t+k} \frac{1(A_t = a)}{p_t(a \mid H_t)} \,\middle|\, S_{kt}\right]
\end{aligned}
$$

B Model Specification

This supplement discusses why the treatment effect at a given lag can be modeled without consideration of treatment effect models at other lags. We also provide a simple example of how models for $\mathrm{E}[W_t Y_{t+k} \mid H_t]$ at different lags k constrain one another and are constrained by and constrain the treatment effect models. These considerations lead us to avoid assumptions concerning the correctness of models for $\mathrm{E}[W_t Y_{t+k} \mid H_t]$. For clarity we consider the case in which $W_t = 1$ for all t and thus illustrate why we avoid assumptions concerning the correctness of models for $\mathrm{E}[Y_{t+k} \mid H_t]$.

From (10), we know that the lag k effect depends on only one of the SNMM treatment blips (8). From (11) these blips are in turn additive on the conditional mean of the potential response. Provided that this conditional mean is not *a priori* restricted to certain values in $(-\infty, \infty)$, the treatment blips do not constrain one another (Robins et al. 2000, Theorem 8.6). This implies the same result for the lag k effect; that is, the treatment effects at different lags can be specified separately, with each lag-specific model imposing no constraints on the models chosen for the treatment effects at the remaining lags.

As an example, here we provide an illustration of how a model chosen for the lag 1 conditional mean response $\mathrm{E}[Y_{t+1} \mid H_t]$ constrains the form of the treatment effects at lag 2. Consider the simple example in which the treatments are binary, randomized with probability 0.5. Suppose we model the conditional mean of the response, $\mathrm{E}[Y_{t+1} \mid H_t]$ by $\alpha_{10} + \alpha_{11} Z_t + \alpha_{12} A_{t-1}$, where Z_t is an binary variable influenced by A_{t-1}. Further suppose that we model the lag 2 treatment effect, $\mathrm{E}[Y_{t+1} \mid A_{t-1} = 1, H_{t-1}] - \mathrm{E}[Y_{t+1} \mid A_{t-1} = 0, H_{t-1}]$ by a linear model $H_{t-1}^{\intercal} \beta_2$. Unfortunately in general these two models are inconsistent; they cannot both be correct. To see this, suppose that unbeknownst to us, $\Pr[Z_t = 1 \mid H_{t-1}] = 1/(1 + \exp(Y_{t-1} + A_{t-1}))$. Now if the first model is correct then the true lag-2 treatment effect should satisfy

$$
\begin{aligned}
& \mathrm{E}[Y_{t+1} \mid A_{t-1} = 1, H_{t-1}] - \mathrm{E}[Y_{t+1} \mid A_{t-1} = 0, H_{t-1}] \\
= \ & \mathrm{E}[\mathrm{E}[Y_{t+1} \mid H_t] \mid A_{t-1} = 1, H_{t-1}] - \mathrm{E}[\mathrm{E}[Y_{t+1} \mid H_t] \mid A_{t-1} = 0, H_{t-1}] \\
= \ & \alpha_{11} \{ \Pr[Z_t = 1 \mid A_{t-1} = 1, H_{t-1}] - \Pr(Z_t = 1 \mid A_{t-1} = 0, H_{t-1}) \} + \alpha_{12} \\
= \ & \alpha_{11} \left\{ \frac{1}{1 + e^{Y_{t-1}+1}} - \frac{1}{1 + e^{Y_{t-1}}} \right\} + \alpha_{12}.
\end{aligned}
$$

In general since the conditional probability of $Z_t = 1$ is constrained to $[0, 1]$, this expression will be non-linear in H_{t-1}. So these lag 2 treatment effect and the lag 1 conditional mean response models cannot both be true.

This example shows that both parsimony in the treatment effect models and correctness in the models for the conditional mean response is difficult to achieve in the presence of binary (or more generally non-continuous) response predictors. Two special scenarios in which models with main effect of the form $g_{kt}(H_t)^{\intercal} \alpha_k$ might be coherent across different k arise when all variables in $g_{kt}(H_t)$ are either (1) multivariate normal, or (2) centered by their conditional mean—i.e., $g_{kt}(H_t)$ is replaced by $g_{kt}(H_t) - \mathrm{E}[g_{kt}(H_t) \mid H_{t-1}]$—since $E[g_{kt}(H_t) - \mathrm{E}[g_{kt}(H_t) \mid H_{t-1}]] = 0$. Both of these settings require strong restrictions or

additional assumptions about the distribution of covariates. So in general we should prefer estimation methods where $g_{kt}(H_t)^\intercal\alpha_k$ need only be a working model for $\mathrm{E}[W_tY_{t+k} \mid H_t]$.

C Large Sample Properties

In this supplement we derive the large sample properties stated in Section 3. Throughout we allow for the setting in which individuals are not always available as discussed in Section 4. For completeness we provide results for a more general estimating function which can be used with observational (non-randomized A_t) treatments, under the assumption of sequential ignorability and assuming the data analyst is able to correctly model and estimate the treatment probability, $P[A_t = 1 \mid H_t]$. We indicate how the results are simplified by use of data from an MRT.

Denote the parameterized treatment probability by $p_t(1 \mid H_t;\eta)$ (with parameter η); note η is known in an MRT. Denote the parameterized numerator of the weights by $\tilde{p}_t(1 \mid S_{kt};\rho)$ (with parameter ρ); below in (14) we will see that the numerator of the weights defines the estimand for $\hat{\beta}_k$ when our modeling assumption (7) is incorrect. In this case, the estimator $\hat{\beta}_k$ converges to the weights on a projection defined by $\tilde{p}_t$. The proof below allows the data analyst to use a $\tilde{p}_t$ with an estimated parameter, $\hat{\rho}$ or to pre-specify ρ as desired. We use a superscript of $*$ to denote limiting values of estimated parameters (e.g. η^*, ρ^*). Then the more general version of the estimating equation Section 3 is

$$\begin{aligned} &U_{\mathrm{W}}(\alpha_k,\beta_k;\hat{\eta},\hat{\rho}) \\ &\quad= \sum_{t=1}^{T-k+1} (Y_{t+k} - g_{kt}(H_t)^\intercal\alpha_k - (A_t - \tilde{p}_t(1|S_{kt};\hat{\rho}))f_{kt}(S_{kt})^\intercal\beta_k) I_t W_t(A_t,H_t;\hat{\eta},\hat{\rho}) \\ &\qquad\qquad \begin{pmatrix} g_{kt}(H_t) \\ (A_t - \tilde{p}_t(1|S_{kt};\hat{\rho}))f_{kt}(S_{kt}) \end{pmatrix} \qquad (12) \end{aligned}$$

where $W_t(A_t, H_t; \eta, \rho) = \tilde{p}_t(A_t \mid S_{kt}; \rho)/p_t(A_t \mid H_t; \eta)$ and $\hat{\eta}, \hat{\rho}$ are estimators. Note W_t in the body of the paper is replaced here by $W_t(A_t, H_t; \hat{\eta}, \hat{\rho})$.

Note that because moments of unavailability ($I_t = 0$) do not contribute to (12) and because (un)availability may change over time (including due to prior treatment; see Section 4), it is not necessarily the case that larger T implies more information for purposes of estimating β_k.

Throughout we assume the model, (7), and sequential ignorability. Assume the following for the k lags of interest.

W1 All entries in $\{Y_{t+k}, g_{kt}(H_t)\}_{t=1}^{t=T-k+1}$ have finite fourth moments.

W2 The matrices $\mathrm{E}\big[\sum_t I_t S_{kt}^{\otimes 2}\big]$ and

$$\mathrm{E}\,\dot{U}_{\mathrm{W}}(\eta^*, \rho^*) = E\sum_t \sum_a I_t \tilde{p}_t(a|S_{kt}; \rho^*) \begin{pmatrix} g_{kt}(H_t) \\ (a - \tilde{p}_t(1|S_{kt}; \rho^*)) f_{kt}(S_{kt}) \end{pmatrix}^{\otimes 2}$$

are invertible.

If the data is observational then we assume:

A3 Treatment Probability Model: $p_t(1 \mid H_t; \eta)$ is a correctly specified model for $\Pr(A_t = 1 \mid I_t = 1, H_t)$. Let η^* be the true value of η; that is, $\Pr(A_t = 1 \mid I_t = 1, H_t) = p_t(1 \mid H_t; \eta^*)$. Assume that the estimator of η, say $\hat{\eta}$, satisfies $\mathbb{P}_n\, U_{\mathrm{D}}(\hat{\eta}) = 0$ and $\sqrt{n}(\hat{\eta} - \eta^*) = \mathrm{E}[\dot{U}_{\mathrm{D}}(\eta^*)]^{-1}\, \mathbb{P}_n\, U_{\mathrm{D}}(\eta^*) + o_P(1)$. Thus $\sqrt{n}(\hat{\eta} - \eta^*)$ converges in distribution to a mean zero, Normal random vector with variance-covariance matrix given by $\mathrm{E}[\dot{U}_{\mathrm{D}}(\eta^*)]^{-1}\, \mathrm{E}[U_{\mathrm{D}}(\eta^*)^{\otimes 2}](\mathrm{E}[\dot{U}_{\mathrm{D}}(\eta^*)]^{-1})^{\intercal}$ which has finite entries. Assume that $\mathbb{P}_n\, \dot{U}_{\mathrm{D}}(\hat{\eta})$ is a consistent estimator of $\mathrm{E}[\dot{U}_{\mathrm{D}}(\eta^*)]$. Assume there exists finite constants, $b_{\mathrm{D}} > 0$ and $B_{\mathrm{D}} < 1$ such that each $b_{\mathrm{D}} < p_t(1 \mid H_t; \eta^*) < B_{\mathrm{D}}$ a.s.

If the data analyst elects to use a parameterized and estimated $\tilde{p}_t(1|S_{kt}, \hat{\rho})$, then we assume:

A4 Numerator of Weights Probability Model: Suppose the estimator $\hat{\rho}$ solves an estimating equation: $\mathbb{P}_n U_{\mathrm{N}}(\rho) = 0$. Assume that, for a finite value of ρ, say ρ^* and $\sqrt{n}(\hat{\rho} - \rho^*) = \mathrm{E}[\dot{U}_{\mathrm{N}}(\rho^*)]^{-1}\sqrt{n}(\mathbb{P}_n - P)U_{\mathrm{N}}(\rho^*) + o_P(1)$ where the matrix, $\mathrm{E}[\dot{U}_{\mathrm{N}}(\rho^*)]$ is positive definite. Assume $\sqrt{n}(\mathbb{P}_n - P)U_{\mathrm{N}}(\rho^*)$ converges in distribution to a mean zero, Normal random vector with variance-covariance matrix given by $\mathrm{E}[U_{\mathrm{N}}(\rho^*)^{\otimes 2}]$ which has finite entries. Assume that $\mathbb{P}_n \dot{U}_{\mathrm{N}}(\hat{\rho})$ is a consistent estimator of $\mathrm{E}[\dot{U}_{\mathrm{N}}(\rho^*)]$. Assume $0 < \rho^* < 1$.

The solution to $\mathbb{P}_n U_{\mathrm{W}}(\alpha_k, \beta_k; \hat{\eta}, \hat{\rho}) = 0$ gives the estimator

$$\begin{pmatrix} \hat{\alpha}_k \\ \hat{\beta}_k \end{pmatrix} = \left\{\mathbb{P}_n \dot{U}_{\mathrm{W}}(\hat{\eta}, \hat{\rho})\right\}^{-1} \mathbb{P}_n \sum_t I_t W_t(A_t, H_t; \hat{\eta}, \hat{\rho}) Y_{t+k} \begin{pmatrix} g_{kt}(H_t) \\ (A_t - \tilde{p}_t(1|S_{kt}; \hat{\rho})) f_{kt}(S_{kt}) \end{pmatrix}$$

where

$$\dot{U}_{\mathrm{W}}(\eta, \rho) = \sum_t I_t W_t(A_t, H_t; \eta, \rho) \begin{pmatrix} g_{kt}(H_t) \\ (A_t - \tilde{p}_t(1|S_{kt}; \rho)) f_{kt}(S_{kt}) \end{pmatrix}^{\otimes 2}.$$

Define

$$\begin{pmatrix} \alpha'_k \\ \beta'_k \end{pmatrix} = \left\{\mathrm{E}\left[\dot{U}_{\mathrm{W}}(\eta^*, \rho^*)\right]\right\}^{-1} \mathrm{E}\left[\sum_t I_t W_t(A_t, H_t; \eta^*, \rho^*) Y_{t+k} \begin{pmatrix} g_{kt}(H_t) \\ (A_t - \tilde{p}_t(1|S_{kt}; \rho^*)) f_{kt}(S_{kt}) \end{pmatrix}\right].$$

Then standard statistical arguments can be used to show that $\sqrt{n}(\hat{\alpha}_k - \alpha'_k, \hat{\beta}_k - \beta'_k)$ converges in distribution to a normal, mean zero, random vector with variance-covariance matrix given by

$$\left\{\mathrm{E}\left[\dot{U}_{\mathrm{W}}(\eta^*, \rho^*)\right]\right\}^{-1} \Sigma_{\mathrm{W}}(\alpha'_k, \beta'_k; \eta^*, \rho^*) \left\{\mathrm{E}\left[\dot{U}_{\mathrm{W}}(\eta^*, \rho^*)\right]\right\}^{-1},$$

where

$$\Sigma_{\mathrm{W}}(\alpha_k, \beta_k; \eta, \rho) = \mathrm{E}\Bigg[\Bigg(U_{\mathrm{W}}(\alpha_k, \beta_k; \eta, \rho) + \Sigma_{\mathrm{W,D}}(\alpha_k, \beta_k; \eta, \rho)\{\mathrm{E}[\dot{U}_{\mathrm{D}}(\eta)]\}^{-1} U_{\mathrm{D}}(\eta)$$

$$+ \Sigma_{\mathrm{W,N}}(\alpha_k, \beta_k; \eta, \rho)\{\mathrm{E}[\dot{U}_{\mathrm{N}}(\rho)]\}^{-1} U_{\mathrm{N}}(\rho)\Big)^{\otimes 2}\Bigg],$$

with

$$\begin{aligned}
&\Sigma_{\mathrm{W,D}}(\alpha_k, \beta_k; \eta, \rho)\\
&\quad = \mathrm{E}\Bigg[\sum_{t=1}^{T-k+1} (Y_{t+k} - g_{kt}(H_t)^\intercal \alpha_k - (A_t - \tilde{p}_t(1|S_{kt};\rho)) f_{kt}(S_{kt})^\intercal \beta_k) I_t W_t(A_t, H_t; \eta, \rho)\\
&\qquad \begin{pmatrix} g_{kt}(H_t) \\ (A_t - \tilde{p}_t(1|S_{kt};\rho)) f_{kt}(S_{kt}) \end{pmatrix} \left(\frac{d \log p_t(A_t \mid H_t; \eta)}{d\eta}\right)^\intercal\Bigg],
\end{aligned}$$

and

$$\begin{aligned}
&\Sigma_{\mathrm{W,N}}(\alpha_k, \beta_k; \eta, \rho)\\
&\quad = \mathrm{E}\Bigg[\sum_{t=1}^{T-k+1} (Y_{t+k} - g_{kt}(H_t)^\intercal \alpha_k - (A_t - \tilde{p}_t(1|S_{kt};\rho)) f_{kt}(S_{kt})^\intercal \beta_k) I_t W_t(A_t, H_t; \eta, \rho)\\
&\qquad \begin{pmatrix} g_{kt}(H_t) \\ (A_t - \tilde{p}_t(1|S_{kt};\rho)) f_{kt}(S_{kt}) \end{pmatrix} \left(\frac{d \log \tilde{p}_t(A_t \mid S_{kt}; \rho)}{d\rho}\right)^\intercal\Bigg]\\
&\quad + \mathrm{E}\Bigg[\sum_{t=1}^{T-k+1} (Y_{t+k} - g_{kt}(H_t)^\intercal \alpha_k - (A_t - \tilde{p}_t(1|S_{kt};\rho)) f_{kt}(S_{kt})^\intercal \beta_k) I_t W_t(A_t, H_t; \eta, \rho)\\
&\qquad \begin{pmatrix} 0_{q\times 1} \\ -\tilde{p}_t(1|S_{kt};\rho) f_{kt}(S_{kt}) \end{pmatrix} \left(\frac{d \log \tilde{p}_t(1 \mid S_{kt}; \rho)}{d\rho}\right)^\intercal\Bigg]\\
&\quad + \mathrm{E}\Bigg[\sum_{t=1}^{T-k+1} \tilde{p}_t(1|S_{kt};\rho) f_{kt}(S_{kt})^\intercal \beta_k I_t W_t(A_t, H_t; \eta, \rho) \begin{pmatrix} g_{kt}(H_t) \\ (A_t - \tilde{p}_t(1|S_{kt};\rho)) f_{kt}(S_{kt}) \end{pmatrix}\\
&\qquad \left(\frac{d \log \tilde{p}_t(1 \mid S_{kt}; \rho)}{d\rho}\right)^\intercal\Bigg]
\end{aligned}$$

where q is the dimension of α_k. Note that if the data is from a MRT (we know p_t) and

we pre-specify (not estimate) $\tilde{p}_t$ then $\Sigma_{\mathrm{W}}(\alpha_k, \beta_k; \eta, \rho) = \mathrm{E}\left[\left(U_{\mathrm{W}}(\alpha_k, \beta_k; \eta, \rho)\right)^{\otimes 2}\right]$ greatly simplifying the variance-covariance matrix.

A consistent estimator of the variance-covariance matrix is given by

$$\left\{\mathbb{P}_n \dot{U}_{\mathrm{W}}(\hat{\eta}, \hat{\rho})\right\}^{-1} \hat{\Sigma}_{\mathrm{W}}(\hat{\alpha}_k, \hat{\beta}_k; \hat{\eta}, \hat{\rho}) \left\{\mathbb{P}_n \dot{U}_{\mathrm{W}}(\hat{\eta}, \hat{\rho})\right\}^{-1}, \tag{13}$$

where

$$\begin{aligned}\hat{\Sigma}_{\mathrm{W}}(\alpha_k, \beta_k; \eta, \rho) = \mathbb{P}_n \Bigg[\Bigg(& U_{\mathrm{W}}(\alpha_k, \beta_k; \eta, \rho) + \hat{\Sigma}_{\mathrm{W,D}}(\alpha_k, \beta_k; \eta, \rho)\{\mathbb{P}_n \dot{U}_{\mathrm{D}}(\eta)\}^{-1} U_{\mathrm{D}}(\eta) \\ & + \hat{\Sigma}_{\mathrm{W,N}}(\alpha_k, \beta_k; \eta, \rho)\{\mathbb{P}_n \dot{U}_{\mathrm{N}}(\rho)\}^{-1} U_{\mathrm{N}}(\rho)\Bigg)^{\otimes 2}\Bigg],\end{aligned}$$

with

$$\begin{aligned}&\hat{\Sigma}_{\mathrm{W,D}}(\alpha_k, \beta_k; \gamma, \eta, \rho) \\ &\quad = \mathbb{P}_n \Bigg[\sum_{t=1}^{T-k+1} (Y_{t+k} - g_{kt}(H_t)^{\intercal}\alpha_k - (A_t - \tilde{p}_t(1|S_{kt}; \rho)) f_{kt}(S_{kt})^{\intercal}\beta_k) I_t W_t(A_t, H_t; \eta, \rho) \\ &\qquad \begin{pmatrix} g_{kt}(H_t) \\ (A_t - \tilde{p}_t(1|S_{kt}; \rho)) f_{kt}(S_{kt}) \end{pmatrix} \left(\frac{d \log p_t(A_t \mid H_t; \eta)}{d\eta}\right)^{\intercal}\Bigg]\end{aligned}$$

and $\hat{\Sigma}_{\mathrm{W,N}}(\alpha_k, \beta_k; \gamma, \eta, \rho) =$

$$\begin{aligned}&\mathbb{P}_n \Bigg[\sum_{t=1}^{T-k+1} (Y_{t+k} - g_{kt}(H_t)^{\intercal}\alpha_k - (A_t - \tilde{p}_t(1|S_{kt}; \rho)) f_{kt}(S_{kt})^{\intercal}\beta_k) I_t W_t(A_t, H_t; \eta, \rho) \\ &\qquad \begin{pmatrix} g_{kt}(H_t) \\ (A_t - \tilde{p}_t(1|S_{kt}; \rho)) f_{kt}(S_{kt}) \end{pmatrix} \left(\frac{d \log \tilde{p}_t(A_t \mid S_{kt}; \rho)}{d\rho}\right)^{\intercal}\Bigg] \\ &\quad + \mathbb{P}_n \Bigg[\sum_{t=1}^{T-k+1} (Y_{t+k} - g_{kt}(H_t)^{\intercal}\alpha_k - (A_t - \tilde{p}_t(1|S_{kt}; \rho)) f_{kt}(S_{kt})^{\intercal}\beta_k) I_t W_t(A_t, H_t; \eta, \rho)\end{aligned}$$

$$\begin{pmatrix} 0_{q\times 1} \\ -\tilde{p}_t(1|S_{kt};\rho) f_{kt}(S_{kt}) \end{pmatrix} \left(\frac{d \log \tilde{p}_t(1 \mid S_{kt};\rho)}{d\rho}\right)^{\intercal}\Bigg]$$

$$+ \mathbb{P}_n \Bigg[\sum_{t=1}^{T-k+1} \tilde{p}_t(1|S_{kt};\rho) f_{kt}(S_{kt})^{\intercal} \beta_k I_t W_t(A_t, H_t; \eta, \rho) \begin{pmatrix} g_{kt}(H_t) \\ (A_t - \tilde{p}_t(1|S_{kt};\rho)) f_{kt}(S_{kt}) \end{pmatrix}$$

$$\left(\frac{d \log \tilde{p}_t(1 \mid S_{kt};\rho)}{d\rho}\right)^{\intercal}\Bigg].$$

It remains to show that $\beta_k' = \beta_k^*$. Since $\mathrm{E}[U_{\mathrm{W}}(\alpha_k', \beta_k'; \gamma^*, \eta^*, \rho^*)] = 0$,

$$\begin{aligned}
0 &= \mathrm{E} \sum_{t=1}^{T-k+1} (Y_{t+k} - g_{kt}(H_t)^{\intercal}\alpha_k' - (A_t - \tilde{p}_t(1|S_{kt};\rho^*)) f_{kt}(S_{kt})^{\intercal}\beta_k') \\
&\qquad I_t w_t(A_t, H_t; \eta^*, \rho^*)(A_t - \tilde{p}_t(1|S_{kt};\rho^*)) f_{kt}(S_{kt}) \\
&= \mathrm{E} \sum_{t=1}^{T-k+1} (\mathrm{E}[Y_{t+k} \mid A_t, H_t, I_t = 1] - g_{kt}(H_t)^{\intercal}\alpha_k' - (A_t - \tilde{p}_t(1|S_{kt};\rho^*)) f_{kt}(S_{kt})^{\intercal}\beta_k') \\
&\qquad I_t w_t(A_t, H_t; \eta^*, \rho^*)(A_t - \tilde{p}_t(1|S_{kt};\rho^*)) f_{kt}(S_{kt}) \\
&= \mathrm{E} \sum_{t=1}^{T-k+1} \sum_{a\in\{0,1\}} (\mathrm{E}[Y_{t+k} \mid A_t = a, H_t, I_t = 1] - g_{kt}(H_t)^{\intercal}\alpha_k' - (a - \tilde{p}_t(1|S_{kt};\rho^*)) f_{kt}(S_{kt})^{\intercal}\beta_k') \\
&\qquad I_t \tilde{p}_t(a|S_{kt};\rho^*)(a - \tilde{p}_t(1|S_{kt};\rho^*)) f_{kt}(S_{kt})
\end{aligned}$$

where the last equality averages out over A_t. The above simplifies to,

$$\begin{aligned}
0 &= \mathrm{E} \sum_{t=1}^{T-k+1} \sum_{a\in\{0,1\}} (\mathrm{E}[Y_{t+k} \mid A_t = a, H_t, I_t = 1] - g_{kt}(H_t)^{\intercal}\alpha_k' - (a - \tilde{p}_t(1|S_{kt};\rho^*)) f_{kt}(S_{kt})^{\intercal}\beta_k') \\
&\qquad I_t \tilde{p}_t(a|S_{kt};\rho^*)(a - \tilde{p}_t(1|S_{kt};\rho^*)) f_{kt}(S_{kt}) \\
&= \mathrm{E} \sum_{t=1}^{T-k+1} (\mathrm{E}[Y_{t+k} \mid A_t = 1, H_t, I_t = 1] - g_{kt}(H_t)^{\intercal}\alpha_k' - (1 - \tilde{p}_t(1|S_{kt};\rho^*)) f_{kt}(S_{kt})^{\intercal}\beta_k') \\
&\qquad I_t \tilde{p}_t(1|S_{kt};\rho^*)(1 - \tilde{p}_t(1|S_{kt};\rho^*)) f_{kt}(S_{kt}) \\
&\quad + (\mathrm{E}[Y_{t+k} \mid A_t = 0, H_t, I_t = 1] - g_{kt}(H_t)^{\intercal}\alpha_k' - (-\tilde{p}_t(1|S_{kt};\rho^*)) f_{kt}(S_{kt})^{\intercal}\beta_k') \\
&\qquad I_t (1 - \tilde{p}_t(1|S_{kt};\rho^*))(-\tilde{p}_t(1|S_{kt};\rho^*)) f_{kt}(S_{kt})
\end{aligned}$$

$$= \mathrm{E} \sum_{t=1}^{T-k+1} f_{kt}(S_{kt})(1 - \tilde{p}_t(1|S_{kt};\rho^*))\tilde{p}_t(1|S_{kt};\rho^*) I_t$$
$$\big(\mathrm{E}[Y_{t+k} \mid A_t = 1, H_t, I_t = 1] - \mathrm{E}[Y_{t+k} \mid A_t = 0, H_t, I_t = 1] - f_{kt}(S_{kt})^\intercal \beta'_k\big).$$

From this we obtain,

$$0 = \mathrm{E} \sum_{t=1}^{T-k+1} f_{kt}(S_{kt})(1 - \tilde{p}_t(1|S_{kt};\rho^*))\tilde{p}_t(1|S_{kt};\rho^*) I_t$$
$$\Big(\mathrm{E}\left[\mathrm{E}[Y_{t+k} \mid A_t = 1, H_t, I_t = 1] - \mathrm{E}[Y_{t+k} \mid A_t = 0, H_t, I_t = 1] \mid S_{kt}, I_t = 1\right]$$
$$- f_{kt}(S_{kt})^\intercal \beta'_k\Big).$$

Thus $\beta'_k =$

$$\left[\mathrm{E}\,\dot{U}_{\mathrm{W}}(\eta^*, \rho^*)\right]^{-1}_{(2,2)} \mathrm{E}\left[\sum_{t=1}^{T-k+1} f_{kt}(S_{kt})(1 - \tilde{p}_t(1|S_{kt};\rho^*))\tilde{p}_t(1|S_{kt};\rho^*) I_t\right.$$
$$\left.\mathrm{E}\left[\mathrm{E}[Y_{t+k} \mid A_t = 1, H_t, I_t = 1] - \mathrm{E}[Y_{t+k} \mid A_t = 0, H_t, I_t = 1] \mid S_{kt}, I_t = 1\right]\right]. \tag{14}$$

where

$$\left[\mathrm{E}\,\dot{U}_{\mathrm{W}}(\eta^*, \rho^*)\right]_{(2,2)} = \mathrm{E} \sum_{t=1}^{T-k+1} f_{kt}(S_{kt}) f_{kt}(S_{kt})^\intercal (1 - \tilde{p}_t(1|S_{kt};\rho^*))\tilde{p}_t(1|S_{kt};\rho^*) I_t.$$

Recall that modeling assumption (7) is,

$$\mathrm{E}\left[\mathrm{E}[Y_{t+k} \mid A_t = 1, H_t, I_t = 1] - \mathrm{E}[Y_{t+k} \mid A_t = 0, H_t, I_t = 1] \mid S_{kt}, I_t = 1\right] = f_{kt}(S_{kt})^\intercal \beta^*_k.$$

From (14), we see that when modeling assumption (7) is incorrect then the data analyst's choice of $\tilde{p}_t(1|S_{kt};\rho^*)$ determines the estimand. Indeed if the data analyst chooses $\tilde{p}_t(1|S_{kt};\rho^*)$

to be a constant then, the limit in probability of $\hat{\beta}_k$ is given by

$$\beta'_k = \left[\mathrm{E} \sum_{t=1}^{T-k+1} f_{kt}(S_{kt}) f_{kt}(S_{kt})^\intercal I_t \right]^{-1} \mathrm{E} \left[\sum_{t=1}^{T-k+1} f_{kt}(S_{kt}) I_t \right. \tag{15}$$

$$\left. \mathrm{E} \Big[\mathrm{E}[Y_{t+k} \mid A_t = 1, H_t, I_t = 1] - \mathrm{E}[Y_{t+k} \mid A_t = 0, H_t, I_t = 1] \mid S_{kt}, I_t = 1 \Big] \right].$$

In the case in which $f_{kt}(S_{kt}) = 1$ (i.e., $S_{kt} = \emptyset$) then the scalar estimand, β'_k, is simply an average (weighted by availability) of proximal treatment effects:

$$\frac{\sum_{t=1}^{T-k+1} \mathrm{E}[I_t] \, \mathrm{E} \Big[\mathrm{E}[Y_{t+k} \mid A_t = 1, H_t, I_t = 1] - \mathrm{E}[Y_{t+k} \mid A_t = 0, H_t, I_t = 1] \mid I_t = 1 \Big]}{\sum_{t=1}^{T-k+1} \mathrm{E}[I_t]}. \tag{16}$$

D Additional simulation results

This section extends the three simulation experiments considered in Section 6 (which focused on $n = T = 30$) to different sample sizes n and number of time points T. Specifically, Table 5, Table 6, and Table 7 below are extensions of Table 1, Table 2, and Table 3, respectively, for the different combinations of $n = 30, 60$ with $T = 30, 50$. In addition, in order to examine the performance of our estimator of the standard error, we provide the Monte Carlo standard deviation of the point estimates (SD) and the Monte Carlo average standard error estimates (SE) for the weighted and centered estimator for all scenarios considered (the SE statistic was not provided in Section 6).

For the first simulation experiment concerning the estimation of a marginal proximal effect when an important moderator exists, see Table 5: In terms of bias, results were similar to those reported in Section 6 for different values of n and T. As before, the weighted and centered estimator was unbiased for all values of β^*_{11}, whereas the bias of the GEE-IND and GEE-AR(1) estimators increased as the magnitude of the underlying effect moderator β^*_{11} increased. In terms of 95% confidence intervals, we note that in these simulations coverage

probabilites for the GEE-IND and GEE-AR(1) estimators generally worsen for larger values of n and T. Finally, in all cases, the average of the standard errors of the proposed weighted and centered estimator closely approximated the Monte Carlo SD.

Table 5: Comparison of three estimators of the marginal proximal treatment effect, $\hat{\beta}_1$, when an important moderator is omitted.

	Weighted and Centered					GEE-IND				GEE-AR(1)			
β_{11}^*	Mean	SD	SE	Root MSE	CP	Mean	SD	Root MSE	CP	Mean	SD	Root MSE	CP
						$n = T = 30$							
0.2	–0.20	0.08	0.08	0.08	0.96	**–0.17**	0.07	0.07	0.94	**–0.16**	0.04	0.06	**0.86**
0.5	–0.20	0.08	0.08	0.08	0.95	**–0.14**	0.07	0.09	**0.88**	**–0.13**	0.05	0.09	**0.70**
0.8	–0.20	0.08	0.08	0.08	0.95	**–0.10**	0.07	0.12	**0.78**	**–0.10**	0.05	0.12	**0.57**
						$n = 30, T = 50$							
0.2	–0.20	0.06	0.06	0.06	0.95	**–0.17**	0.05	0.06	**0.92**	**–0.16**	0.03	0.05	**0.73**
0.5	–0.20	0.06	0.06	0.06	0.95	**–0.14**	0.06	0.08	**0.80**	**–0.13**	0.04	0.08	**0.49**
0.8	–0.20	0.07	0.07	0.07	0.94	**–0.11**	0.06	0.11	**0.64**	**–0.10**	0.04	0.11	**0.32**
						$n = 60, T = 30$							
0.2	–0.20	0.06	0.05	0.06	0.95	**–0.17**	0.05	0.06	**0.90**	**–0.16**	0.03	0.05	**0.72**
0.5	–0.20	0.06	0.06	0.06	0.95	**–0.14**	0.05	0.08	**0.76**	**–0.13**	0.03	0.08	**0.41**
0.8	–0.20	0.06	0.06	0.06	0.94	**–0.11**	0.06	0.11	**0.56**	**–0.10**	0.04	0.11	**0.25**
						$n = 60, T = 50$							
0.2	–0.20	0.04	0.04	0.04	0.94	**–0.17**	0.04	0.05	**0.87**	**–0.16**	0.02	0.05	**0.55**
0.5	–0.20	0.04	0.04	0.04	0.95	**–0.14**	0.04	0.07	**0.59**	**–0.13**	0.02	0.08	**0.19**
0.8	–0.20	0.05	0.05	0.05	0.95	**–0.10**	0.04	0.11	**0.33**	**–0.10**	0.03	0.11	**0.06**

MSE, mean squared error, and SD, standard deviation of $\hat{\beta}_1$; SE, average of the standard errors for the weighted and centered estimator; CP, 95% confidence interval coverage probability for $\beta_1^* = -0.2$. Results are based on 1000 replicates. Boldface indicates whether Mean or CP are significantly different, at the 5% level, from -0.2 or 0.95, respectively.

For the second simulation experiment concerning the stabilization of the weights W_t in the proposed approach, see Table 6: results were similar to those reported in Section 6. In all cases, the average of the standard errors of the proposed weighted and centered estimator closely approximated the Monte Carlo SD.

Table 6: Weighted and centered estimator of the marginal proximal treatment effect, $\hat{\beta}_1$, using two choices for $\tilde{p}_t$.

		$\tilde{p}_t$ is constant (i)					$\tilde{p}_t$ depends on S_t (ii)				
n	T	Mean	SD	SE	RMSE	CP	Mean	SD	SE	RMSE	CP
30	30	–0.20	0.08	0.08	0.08	0.94	**–0.14**	0.09	0.09	0.11	**0.89**
	50	–0.20	0.06	0.06	0.06	0.95	**–0.14**	0.07	0.07	0.09	**0.86**
60	30	–0.20	0.06	0.06	0.06	0.95	**–0.14**	0.06	0.06	0.09	**0.83**
	50	–0.20	0.04	0.04	0.04	0.94	**–0.14**	0.05	0.05	0.08	**0.72**

RMSE, root mean squared error, and SD, standard deviation of $\hat{\beta}_1$; SE, average of the standard errors for the proposed estimator with appropriate $\tilde{p}_t$; CP, 95% confidence interval coverage probability for $\beta_1^* = -0.2$. Results are based on 1000 replicates. Boldface indicates whether Mean or CP are significantly different, at the 5% level, from -0.2 or 0.95, respectively.

For the third simulation experiment concerning the use of a non-independent working correlation structure in the proposed approach, see Table 7: results were similar to those in Section 6, with worsening CP under the non-independent working correlation for larger n. In all cases, the average of the standard errors of the proposed weighted and centered estimator (with an independent working correlation) closely approximated the Monte Carlo SD.

Table 7: Weighted and centered estimator of the marginal proximal effect, $\hat{\beta}_1$, with different working correlation structures.

		Independent working correlation (i)					AR(1) working correlation (ii)			
n	T	Mean	SD	SE	RMSE	CP	Mean	SD	RMSE	CP
30	30	–0.20	0.07	0.07	0.07	0.96	**–0.13**	0.06	0.09	**0.66**
	50	–0.20	0.05	0.05	0.05	0.96	**–0.13**	0.03	0.07	**0.47**
60	30	–0.20	0.05	0.05	0.05	0.94	**–0.14**	0.03	0.07	**0.42**
	50	–0.20	0.04	0.04	0.04	0.95	**–0.13**	0.02	0.07	**0.16**

RMSE, root mean squared error, and SD, standard deviation of $\hat{\beta}_1$; SE, average of the standard errors for the proposed estimator with independent working correlation; CP, 95% confidence interval coverage probability for $\beta_1^* = -0.2$. Results are based on 1000 replicates. Boldface indicates whether Mean or CP are significantly different, at the 5% level, from -0.2 or 0.95, respectively.

Recall that, for simplicity, the simulation experiments focus on a simplistic setting where individuals are always available (i.e., $I_t = 1$ for all t) and the marginal proximal effect is constant over time. In this special case, we observe that $\text{Var}(\hat{\beta}_k)$ decreases as T gets large; however, this result is not expected to be true, in general.

E Code to Generate Simulation Results

The R code used to generate the simulation experiment results in this paper can be obtained from https://github.com/dalmiral/mHealthModeration. This includes the additional calculations necessary to correct standard errors for small samples and for estimated weights (i.e., when either $\tilde{p}_t(1 \mid S_{kt})$ or $p_t(1 \mid H_t)$ is estimated).
